# Characterization of *Aspergillus fumigatus* Isolates from Air and Surfaces of the International Space Station

**DOI:** 10.1128/mSphere.00227-16

**Published:** 2016-10-26

**Authors:** Benjamin P. Knox, Adriana Blachowicz, Jonathan M. Palmer, Jillian Romsdahl, Anna Huttenlocher, Clay C. C. Wang, Nancy P. Keller, Kasthuri Venkateswaran

**Affiliations:** a Microbiology Doctoral Training Program, University of Wisconsin—Madison, Madison, Wisconsin, USA; b Department of Medical Microbiology and Immunology, University of Wisconsin—Madison, Madison, Wisconsin, USA; c Biotechnology and Planetary Protection Group, Jet Propulsion Laboratory, California Institute of Technology, Pasadena, California, USA; d Department of Pharmacology and Pharmaceutical Sciences, School of Pharmacy, University of Southern California, Los Angeles, California, USA; e Center for Forest Mycology Research, Northern Research Station, U.S. Forest Service, Madison, Wisconsin, USA; f Department of Pediatrics, University of Wisconsin—Madison, Madison, Wisconsin, USA; g Department of Chemistry, Dornsife College of Letters, Arts and Sciences, University of Southern California, Los Angeles, California, USA; h Department of Bacteriology, University of Wisconsin—Madison, Madison, Wisconsin, USA; Yonsei University

**Keywords:** *Aspergillus fumigatus*, International Space Station, SNP analysis, secondary metabolites, virulence

## Abstract

One mission of the Microbial Observatory Experiments on the International Space Station (ISS) is to examine the traits and diversity of fungal isolates to gain a better understanding of how fungi may adapt to microgravity environments and how this may affect interactions with humans in a closed habitat. Here, we report an initial characterization of two isolates, ISSFT-021 and IF1SW-F4, of *Aspergillus fumigatus* collected from the ISS and a comparison to the experimentally established clinical isolates Af293 and CEA10. Whole-genome sequencing of ISSFT-021 and IF1SW-F4 showed 54,960 and 52,129 single nucleotide polymorphisms, respectively, compared to Af293, which is consistent with observed genetic heterogeneity among sequenced *A. fumigatus* isolates from diverse clinical and environmental sources. Assessment of *in vitro* growth characteristics, secondary metabolite production, and susceptibility to chemical stresses revealed no outstanding differences between ISS and clinical strains that would suggest special adaptation to life aboard the ISS. Virulence assessment in a neutrophil-deficient larval zebrafish model of invasive aspergillosis revealed that both ISSFT-021 and IF1SW-F4 were significantly more lethal than Af293 and CEA10. Taken together, these genomic, *in vitro*, and *in vivo* analyses of two *A. fumigatus* strains isolated from the ISS provide a benchmark for future investigations of these strains and for continuing research on specific microbial isolates from manned space environments.

**IMPORTANCE** As durations of manned space missions increase, it is imperative to understand the long-term consequence of microbial exposure on human health in a closed human habitat. To date, studies aimed at bacterial and fungal contamination of space vessels have highlighted species compositions biased toward hardy, persistent organisms capable of withstanding harsh conditions. In the current study, we assessed traits of two independent *Aspergillus fumigatus* strains isolated from the International Space Station. Ubiquitously found in terrestrial soil and atmospheric environments, *A. fumigatus* is a significant opportunistic fungal threat to human health, particularly among the immunocompromised. Using two well-known clinical isolates of *A. fumigatus* as comparators, we found that both ISS isolates exhibited normal *in vitro* growth and chemical stress tolerance yet caused higher lethality in a vertebrate model of invasive disease. These findings substantiate the need for additional studies of physical traits and biological activities of microbes adapted to microgravity and other extreme extraterrestrial conditions.

## Abstract (precis)

As durations of manned space missions increase, it is imperative to understand the long-term consequence of microbial exposure on human health in a closed human habitat. To date, studies aimed at bacterial and fungal contamination of space vessels have highlighted species compositions biased toward hardy, persistent organisms capable of withstanding harsh conditions. In the current study, we assessed traits of two independent *Aspergillus fumigatus* strains isolated from the International Space Station. Ubiquitously found in terrestrial soil and atmospheric environments, *A. fumigatus* is a significant opportunistic fungal threat to human health, particularly among the immunocompromised. Using two well-known clinical isolates of *A. fumigatus* as comparators, we found that both ISS isolates exhibited normal *in vitro* growth and chemical stress tolerance yet caused higher lethality in a vertebrate model of invasive disease. These findings substantiate the need for additional studies of physical traits and biological activities of microbes adapted to microgravity and other extreme extraterrestrial conditions.

## INTRODUCTION

Microorganisms are unavoidable inhabitants of human-made structures in space due to anthropogenic sources, including human and cargo movement (1). Our understanding of how the stressors of such environments, which include microgravity and increased exposure to irradiation, influence microbial biology over time remains in its infancy (2). Changes in microbial community composition and microbial species characteristics have the potential to affect human health and safety, particularly in light of the fact that extended periods of time in space have been shown to alter vertebrate and human immunity (3, 4). Furthermore, as durations of manned space missions increase, such as going to Mars, it becomes of heightened importance to understand the breadth and potential consequences of host-microbe interactions in crew habitation. There exists an unmet need for studies characterizing individual microbial species isolated directly from space environments, as sampling experiments to date have aimed at understanding changes in microbial community composition at the species level (4–6), and it has been documented that simulated space environments provide a poor comparison for what is actually observed in orbit (7), highlighting a need for additional experimentation with samples derived from space environments.

While, unsurprisingly, most of the sampled bacterial diversity from space environments aligns with commensal organisms (8), many fungi represent a unique component of microbial communities in space environments, as their populations are not replenished by virtue of human presence, suggesting they have exploited or adapted to a proliferative niche aboard these human-made structures. Fungal colonization of space vessels is nothing new, as various species have been isolated from the Skylab, Mir, and various modules of the International Space Station (ISS) (United States, Japan’s KIBO, and the Russian segments) (5, 6, 9–13). Fungi have been reported to cause damage to electrical and structural components through the decomposition of wire insulation and window gaskets (13). The most commonly sampled fungal genera from space environments include the terrestrially ubiquitous sporulating molds *Cladosporium*, *Penicillium*, and *Aspergillus*. Airborne spores, also known as conidia, are ubiquitous in terrestrial environments and can exacerbate pulmonary allergic reactions (14) and cause life-threatening invasive infections after germinating in immunocompromised individuals (15, 16). Among airborne fungi, *Aspergillus fumigatus* is the most frequently encountered agent of pulmonary complications and invasive infections, as infections can result in invasive aspergillosis (IA) in immunocompromised populations, with average mortality rates of 50% even with proper diagnosis and treatment (17).

Globally encountered in soil and air, *A. fumigatus* is well adapted to colonizing diverse environments through its metabolic diversity, broad stress and thermal tolerances, and easily dispersed conidia (18–20). Likely underlying the ubiquity and pathogenic capacity of *A. fumigatus* is a great degree of genetic diversity observed among strains from diverse environmental and clinical sources (21–23). While many factors may contribute to the ubiquity of *A. fumigatus*, the production of small bioactive molecules, or secondary metabolites (SMs), has become of particular interest, as these compounds have been shown to play central roles in niche exploitation, stress tolerance, and virulence (17, 24, 25). Considering *A. fumigatus* as a ubiquitously encountered opportunistic pathogen with great metabolic and genetic diversity, it is an organism of particular interest to monitor and examine as a contaminant of human space vessels.

Here, we report an initial characterization of two *A. fumigatus* strains isolated from different sources of the ISS. Our experimental approach aimed to investigate each strain’s genetic origins and characteristics, *in vitro* growth and stress tolerance, secondary metabolite production, and virulence. Given the importance of *A. fumigatus* as an opportunistic pathogen, both ISS strains were studied in comparison to two well-known clinical isolates to assess pathogenic traits that may be of consequence to human health.

## RESULTS

### Identification of *A. fumigatus* sampled from the ISS.

**FIG 1:**
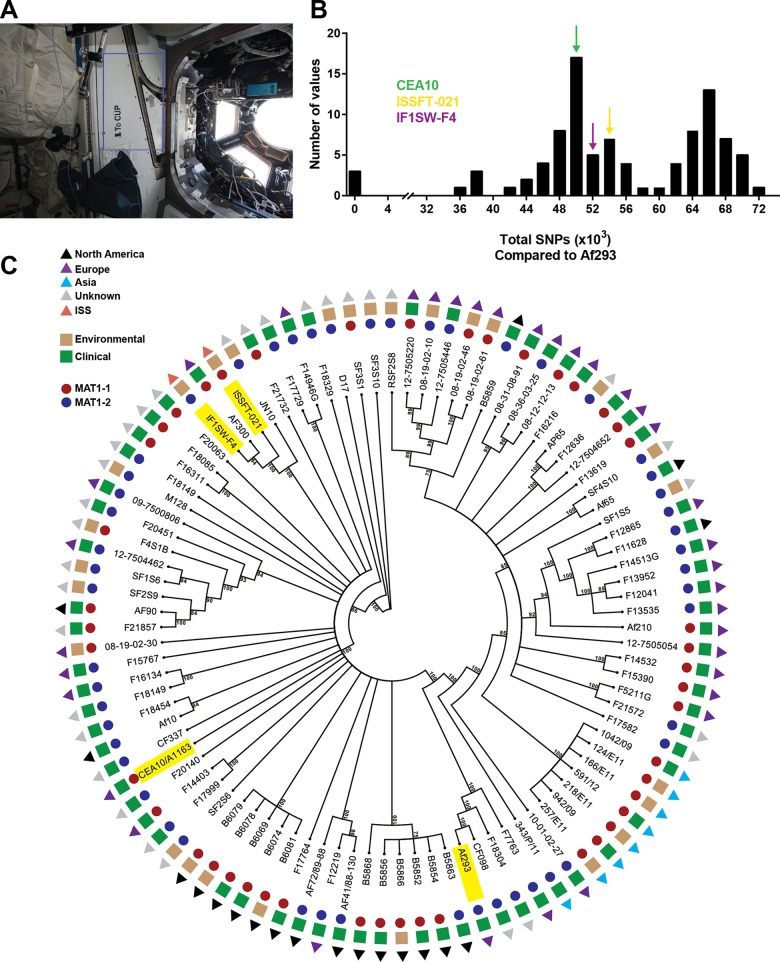
Isolation and phylogenetic characterization of ISS strains. (A) IF1SW-F4 was isolated from the wall area outlined in blue adjacent to the cupola window aboard the ISS. ISSFT-021 was independently isolated from a HEPA filter (not shown). (B) Frequency distribution of total SNPs found in 94 sequenced clinical and environmental isolates of *A. fumigatus* in comparison to a reference genome (Af293). Colored arrows designate the bin groups to which each strain included in this study belongs. (C) Phylogenetic tree of 95 sequenced isolates of *A. fumigatus*, showing mating type (MAT1-1 or MAT1-2), clinical or environmental origin, and geographical sampling location. Strains of interest used in this study are highlighted in yellow.

Air and surface sampling during the Microbial Observatory Experiments on the ISS identified numerous bacterial and fungal isolates (7). For this study, we chose to focus on two independently sampled strains of *A. fumigatus* which were initially identified by morphological characteristics and later verified by internal transcribed spacer (ITS) region sequencing (ITS sequences for ISSFT-021 and IF1SW-F4 are available under GenBank accession numbers KT832787 and KX675260, respectively). Strain ISSFT-021 was sampled from a high-efficiency particulate arrestance (HEPA) filter, and strain IF1SW-F4 was obtained from a hard surface adjacent to the cupola window (Fig. 1A) via wiping surface materials. By nature of this sampling method, it is impossible to know exact residence times aboard the ISS for each strain.

Whole-genome sequencing (WGS) of ISSFT-021 and IF1SW-F4 (26) reveal 54,960 and 52,129 single nucleotide polymorphisms (SNPs) compared to the clinical isolate and model laboratory strain Af293, respectively, which is not outside the genetic diversity observed among 95 sequenced *A. fumigatus* isolates (Fig. 1B). Figure 1C shows a phylogeny of these isolates of *A. fumigatus* inferred using maximum likelihood from SNP sequences covered in every genome (147,792 total SNP positions). The tree also includes information on individual isolate mating type, clinical or environmental origin, and also geographical location. This analysis illustrates the genomic variation that exists with *A. fumigatus* and, moreover, suggests that we are unable to predict phenotypic characteristics (i.e., virulence) based on clinical/environmental origin, mating type, or geographical isolation source. With regard to mating type, strains ISSFT-021 and CEA10 (another clinical isolate and model laboratory strain) are MAT1-1, while IF1SW-F4 and Af293 are MAT1-2. Interestingly, ISSFT-021 and IF1SW-F4 show a close relationship to the patient isolate Af300 (27), suggesting these three strains may have arisen from a common origin. Figure S1 in the supplemental material shows the same phylogenic data represented in a phylogram. As it has been suggested previously that genetic consequences resulting from irradiation exposure during time in space may manifest as insertions and deletions (indels) over point mutations (28–30), we analyzed ISSFT-021, IF1SW-F4, and CEA10 and averages from all sequenced isolates included in this study against the reference genome (Af293) and found no obvious enrichment for indels in the ISS isolates (see Fig. S2 in the supplemental material).

10.1128/mSphere.00227-16.1 Figure S1  Phylogram of 95 sequenced *A. fumigatus* isolates. The phylogram represents the same data used to create the cladogram in Fig. 1C. All strains included in the present study are highlighted in red. Download Figure S1, PDF file, 0.7 MB. Copyright © 2016 Knox et al. 2016 Knox et al. This content is distributed under the terms of the Creative Commons Attribution 4.0 International license.

10.1128/mSphere.00227-16.2 Figure S2  Insertion and deletion size range and frequency distribution. Indel sizes and frequencies of occurrence in relation to that in the reference genome (Af293) are shown for CEA10, ISSFT-021, and IF1SW-F4, along with averages for all isolates included in the genomic analyses. Download Figure S2, PDF file, 0.3 MB. Copyright © 2016 Knox et al. 2016 Knox et al. This content is distributed under the terms of the Creative Commons Attribution 4.0 International license.

### Visual characterization and growth rates of ISS strains *in vitro*.

**FIG 2:**
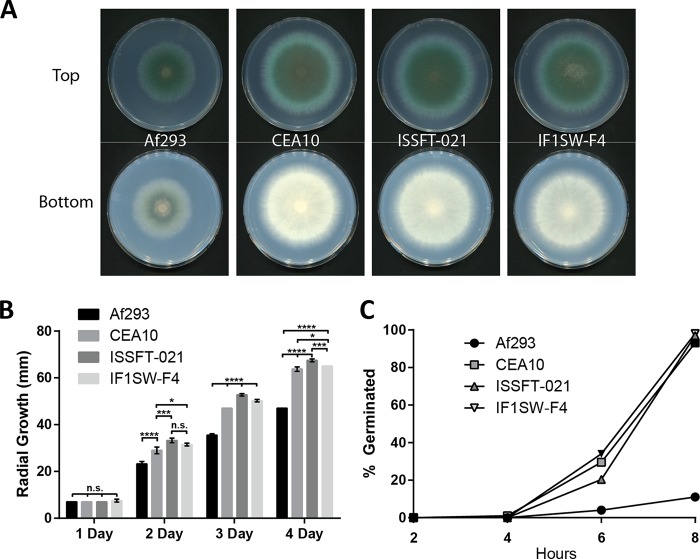
*In vitro* growth of ISS isolates compared to growth of the clinical isolates Af293 and CEA10. (A) Growth on GMM at 37°C, showing colony morphology and color. (B) Radial growth at 37°C on GMM. Statistical analyses were performed by one-way ANOVA. (C) Germination rates in liquid GMM at 37°C, 250 rpm. Spores were considered germinated after germ tube lengths were observed to be greater than or equal to the swollen spore base.

To assess basic physiological phenotypes of strains ISSFT-021 and IF1SW-F4, growth characteristics of the ISS strains were investigated on defined glucose minimal medium (GMM) (31). Gross visual assessment of point-inoculated GMM plates revealed slight differences in colony diameter and pigment production after a 5-day incubation (Fig. 2A), indicating that each strain possesses unique physical and chemical properties under these conditions. An examination of radial growth rates revealed that both ISS strains significantly outgrew both Af293 and CEA10 at all time points investigated (Fig. 2B), which is consistent with previous reports of strain-dependent variations in growth rates of *A. fumigatus* isolates (21) as well as increased biomass production for some microbes during exposure to space environments (32). As early spore germination rates may favor niche exploitation and establishment of robust growth (21), we next sought to determine whether ISS isolates exhibited different germination dynamics *in vitro*. Figure 2C shows there were no differences in germination rates between ISSFT-021, IF1SW-F4, and CEA10 in liquid GMM, as nearly all spores germinated after an 8-h incubation, while Af293 showed a marked delay in germination.

### ISS strains show no enhanced resistance to chemical stresses *in vitro.*

**FIG 3:**
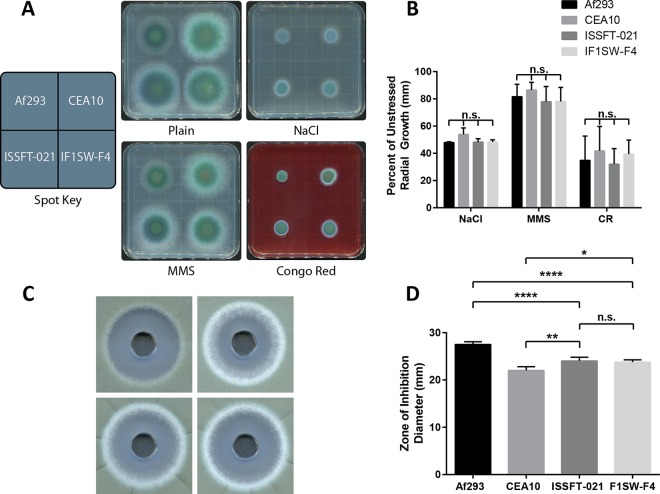
ISS isolates showed no enhanced resistance to chemical stresses *in vitro*. (A) Colony appearance after point inoculations of 10 µl containing 1 × 10^4^ conidia on solid GMM supplemented with the following stressors: 1.0 M NaCl, 0.02% MMS, and 25 mg/ml Congo red (CR). (B) Quantification of growth inhibition was measured by colony diameters after a 72-h incubation at 37°C. Data shown are the radial growth versus that in controls. (C) Hydrogen peroxide sensitivity was assayed by diffusion assay with 1-cm holes filled with 100 µl of 4% H_2_O_2_ in plates containing 5 × 10^7^ spores suspended in top agar. (D) Zones of inhibition were measured as diameters after 48 h of growth, and significance determined via one-way analysis of variance.

While unstressed growth (Fig. 2) revealed several differences between ISS and patient isolate strains, it is reasonable to posit that resistance to stressful conditions may play a greater role in colonization and propagation aboard harsh space vessel environments. Therefore, we challenged all strains to a variety of classical chemical stresses on GMM to assay susceptibility to osmotic stress (sodium chloride [NaCl]), DNA damage stress (methyl methanesulfonate [MMS]), cell wall stress (Congo red), and oxidative stress (hydrogen peroxide [H_2_O_2_]) (Fig. 3). Figure 3A shows colony appearance and growth reduction on supplemented media compared to that under unstressed growth conditions. No significant differences in growth reduction were observed for either ISS strain in comparison to Af293 or CEA10 (Fig. 3B). For oxidative stress tests, we chose a diffusion assay (33) to reduce experimental variation in working with this chemical (unpublished observations). Zones of inhibition (Fig. 3C) were measured to infer sensitivity to hydrogen peroxide. No significant differences were observed between ISS strains regarding hydrogen peroxide sensitivity (Fig. 3D); however, both were significantly more resistant than Af293 and less resistant than CEA10, demonstrating an intermediate phenotype of ISS isolates between the two clinical strains.

### Secondary metabolite analysis among ISS and clinical isolates.

**FIG 4:**
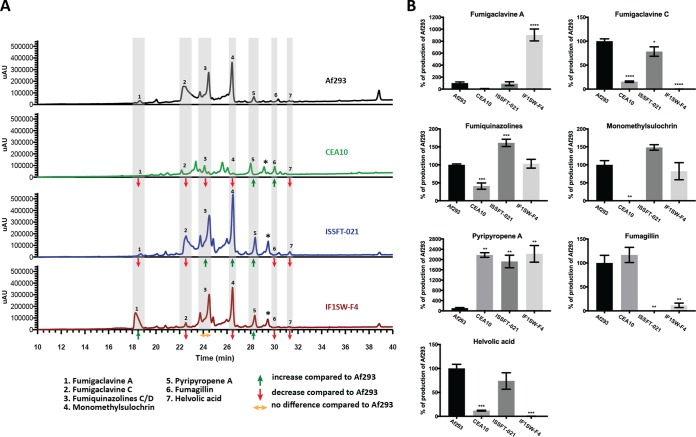
Secondary metabolite production of ISS strains. (A) Secondary metabolite profiles of ISSFT-021, IF1SW-F4, CEA10, and Af293 when grown on GMM. Individual metabolite production is reported as either increased, decreased, or no difference. compared to that of Af293. (B) Metabolite quantification, showing the percent change for each metabolite in relation to Af293; significance was determined using a one-way ANOVA.

SM profiles of ISSFT-021, IF1SW-F4, CEA10, and Af293 were examined after culture on solid GMM by using high-performance liquid chromatography–photodiode array detection–mass spectroscopy (HPL-DAD-MS) analysis. Examination of the SM profiles revealed a distinct chemical signature for each strain under the condition tested (Fig. 4A). Detailed yield analysis of each SM produced was carried out (Fig. 4B). Compared to Af293, an increase in fumigaclavine A production was observed with IF1SW-F4 (*P* = 0.0001) but not with ISSFT-021, whereas a significant decrease in fumigaclavine C production was noticed in both strains (*P* = 0.04 and 0.0001 in ISSFT-021 and IF1SW-F4, respectively). Fumiquinazoline production increased in ISSFT-021 (*P* = 0.0004) but not in IF1SW-F4 compared to the two controls. Pyripyropene A production increased in both ISSFT-021 and IF1SW-F4 (*P* = 0.0063 and 0.0018, respectively). Observed SM yields for CEA10 were lower than for any other strain, with an observed decrease in production of all but two compounds (pyripyropene A and fumagillin) (Fig. 4A). Nevertheless, as production of SMs in media does not necessarily replicate the pattern of SM production during infections, the potential *in vivo* SM profiles of the ISS strains remain unclear.

While a detailed analysis of SNPs with potential consequence on SM regulation and production was beyond the scope of the present study, Data Set S1 in the supplemental material shows polymorphisms within 26 SM gene clusters that produce either known SMs or have strong bioinformatic support to produce a likely SM (34). The combined analysis of CEA10, ISSFT-021, and IF1SW-F4 identified 1,578 variants in comparison with Af293 within the boundaries of the 26 SM gene clusters, and 504 of these variants were predicted to result in nonsynonymous substitution in an SM cluster gene (see Data Set S1). Importantly, our analysis corroborated the previously published point mutation R202L in FtmD (Afu8g00200), which results in loss of fumitremorgin production in Af293 (35), as well as the previously described frameshift mutation in TpcC in the CEA10 trypacidin cluster that leads to loss of function of the trypacidin polyketide synthase (36). A metabolite between pyripyropene A (peak no. 5) and fumagillin (peak no. 6) (Fig. 4A, starred) was observed via liquid chromatography-mass spectrometry (LC/MS) in the CEA10, ISSFT-021, and IF1SW-F4 strains (see Fig. S3 in the supplemental material). We used high-resolution mass spectrometry (494.2276 positive mode) to obtain a proposed molecular formula of C_27_H_31_O_6_N_3_ for the compound (see Fig. S4 in the supplemental material). A chemical database (Reaxys) search using the proposed formula revealed no known *A. fumigatus* metabolite that matched this formula. However, the metabolites versicamide F from *Aspergillus versicolor* (37) and taichunamides C and F from *Aspergillus taichungensis* (38) matched the proposed formula. These findings might indicate that the compound is a previously uncharacterized prenylated indole alkaloid produced by *A. fumigatus*.

10.1128/mSphere.00227-16.3 Figure S3  Secondary metabolite production of ISS strains, with a focus on an unknown *A. fumigatus* compound. Secondary metabolite profiles shown are for ISSFT-021, IF1SW-F4, and CEA10 when grown on glucose minimal medium. Traces present PDA, total, and extracted ion counts and ESIMS (positive mode) data for the unknown compound analyzed by LC/MS. Download Figure S3, PDF file, 1.2 MB. Copyright © 2016 Knox et al. 2016 Knox et al. This content is distributed under the terms of the Creative Commons Attribution 4.0 International license.

10.1128/mSphere.00227-16.4 Figure S4  High-resolution mass spectrometry of unknown compound. ESIMS (positive mode) spectrum of a new, unknown compound, with its proposed molecular formula. Download Figure S4, PDF file, 0.7 MB. Copyright © 2016 Knox et al. 2016 Knox et al. This content is distributed under the terms of the Creative Commons Attribution 4.0 International license.

10.1128/mSphere.00227-16.5 Data Set S1  Polymorphisms in 26 *A. fumigatus* SM biosynthetic clusters. Using Af293 as the reference genome, polymorphisms within 26 supported SM biosynthetic clusters found in CEA10, ISSFT-021, and IF1SW-F4 are detailed as deletions, insertions, multiple nucleotide variants (MNV), replacements, or single nucleotide variants (SNV). Download Data Set S1, PDF file, 1.6 MB. Copyright © 2016 Knox et al. 2016 Knox et al. This content is distributed under the terms of the Creative Commons Attribution 4.0 International license.

### ISS strains exhibit increased virulence in a vertebrate model of invasive aspergillosis.

**FIG 5:**
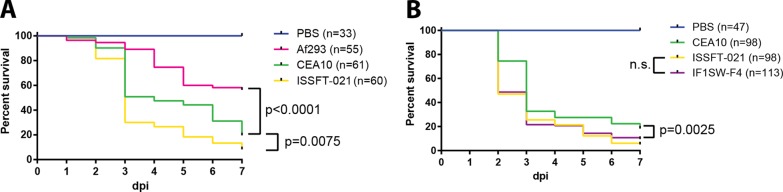
Virulence assessment in a larval zebrafish model of invasive aspergillosis. (A) Survival outcome through 7 days postinfection (dpi) of neutrophil-deficient *mpx:mCherry-2A-Rac2D57N* larvae, in which neutrophils specifically are unable to reach the site of infection. (B) Survival outcome of the second ISS isolate, IF1SW-F4, compared to that of CEA10 and ISSFT-021 in *mpx:mCherry-2A-Rac2D57N* larvae. Shown are data pooled from three (A) or four (B) independent experimental replicates. Statistical analyses were performed using the Cox proportional hazard regression analysis.

Given the ISS’s intimate environment and potential for frequent exposure between astronauts and *A. fumigatus*, it is of high importance to investigate the virulence potential of these isolates. Therefore, we tested the virulence of both ISS strains against CEA10 and Af293 in a larval zebrafish model of IA, which has been shown to recapitulate key aspects of disease observed in murine models and human disease (39, 40). As immunocompetent zebrafish larvae, like humans and mice, do not succumb to lethal infection following *A. fumigatus* challenge (39), we utilized neutrophil-deficient larvae [*Tg*(*mpx*:*mCherry-2A-Rac2D57N*)] (41) to investigate virulence differences between strains. Recapitulating previous reports in a murine infection model (27), we found that CEA10 was more virulent than Af293 (*P* < 0.0001), while ISSFT-021 caused significantly more lethality than CEA10 (*P* = 0.0075) (Fig. 5A). Furthermore, no significant difference was observed in virulence between ISSFT-021 and IF1SW-F4 (*P* > 0.1), while IF1SW-F4, like ISSFT-021 (Fig. 5A), was significantly more virulent than CEA10 (*P* = 0.0025) (Fig. 5B).

## DISCUSSION

In the present study, we investigated two independently isolated strains of *A. fumigatus* from the ISS for differences in genetic makeup, growth characterizations, stress tolerance, secondary metabolite production, and virulence. While microbial colonization of space vessels is unavoidable and is well-established in the literature (1, 8), less understood is how the unique stresses found aboard space vessels, such as increased irradiation and microgravity, affect microorganisms and their biology. Therefore, as *A. fumigatus* is the most significant airborne opportunistic mold pathogen of humans (42), it became prudent to investigate the ISS *A. fumigatus* strains for factors that may affect health-related interactions with astronauts aboard the ISS and initiate lines of investigation into how space flight may influence this particular pathogen.

Samples from HEPA filters, 16 surface locations, 12 air samples, and debris collected using vacuum cleaners across multiple spaceflights during the Microbial Observatory Experiments (7) revealed the presence of many microbial species aboard the ISS. Among ~200 bacterial and fungal isolates identified using molecular methods (7), fungal strains were particularly enriched with members of the genera *Penicillium*, *Aspergillus*, and *Rodoturulla*. However, only two isolates were identified as *A. fumigatus* from these samples; one each from a 40-month-old HEPA filter (ISSFT-021) and a surface location adjacent to the cupola window (IF1SW-F4) (Fig. 1A). Interestingly, since the inception of the ISS, ~20 years of environmental monitoring did not reveal the presence of *A. fumigatus* (43). Considering the environmental ubiquity of this species, this anomaly might be partly due to collections from small surface areas (25 cm^2^), compared to the larger 1-m^2^ sampling areas adapted during this study. In addition, regular ISS operation and environmental monitoring protocols utilized conventional methods, in which the isolation of *Aspergillus* was reported but not identified to the species level (43). Unfortunately, ISS operations did not archive any fungal strains isolated during these 20 years of operation; therefore, we could not address whether or not any of the *Aspergillus* isolates were *A. fumigatus*. Hence, even though *A. fumigatus* is ubiquitous in the terrestrial atmospheres and would thus be an unsurprising contaminant of the ISS, this is the first report about the isolation of *A. fumigatus* from the ISS.

Following initial culture and ITS sequence-based identification of the isolates as *A. fumigatus*, we undertook WGS to facilitate the current and future studies of ISSFT-021 and IF1SW-F4 (26). Analysis of SNPs among all publicly available sequenced isolates (>100 total; 95 unique isolates included in the present study) across a global range of clinical and environmental sources shows considerable genetic diversity, consistent with previous reports of genetic variance in *A. fumigatus* (22). Given that we are unable to know how, when, and where ISSFT-021 and IF1SW-F4 initially colonized the ISS, it is curious that both strains bare the closest relationship to the strains Af300 and JN10 (27) (Fig. 1C; see also Fig. S1 in the supplemental material). Af300 was isolated in 1995 in Manchester, United Kingdom, from a leukemia patient (David Denning, personal communication), and JN10 is an environmental isolate of unknown sampling origin. Further clouding the origins of these strains is that cargo shipments to the ISS are manufactured and launched from all over the world.

Notably, both ISS isolates show no enhanced accumulation of SNPs (Fig. 1B; see also Fig. S2 in the supplemental material), suggesting that life aboard the space station was not accompanied by an accumulation of mutations presumably from enhanced exposure to irradiation and microgravity; however, since the proper terrestrial control strains do not exist for ISSFT-021 and IF1SW-F4, we cannot determine/quantify mutations that may have accumulated during time aboard the ISS. Interestingly, previous data suggest that DNA damage resulting from time aboard the ISS may favor chromosomal aberrations and large deletions over point mutations (28–30), a conclusion that may have been fueled by studies that reported finding no detectable mutations from time in space, although these experiments utilized experimental setups that would favor detection of point mutations over large genetic lesions, or the studies were possibly too short in duration for mutations to accumulate (44). Sequence analysis with ISS *A. fumigatus* strains suggested that there is no enrichment for any type of mutation we could identify through our resequencing-based mapping approach, namely, indels, in comparison with Af293 (see Fig. S2), yet it remains to be determined whether *A. fumigatus* possesses inherent characteristics that would facilitate maintenance of genomic integrity over other biological systems in space (including humans and other eukaryotes) from which mutation data have been inferred.

Our reported *in vitro* analysis showing increased radial growth rates for space isolates versus clinical isolates (Fig. 2) is not without precedent, as enhanced fungal growth from *Aspergillus* and *Penicillium* species recovered from Mir, compared to terrestrial reference strains, has previously been reported (8). However, there is a considerable difference among growth rates for terrestrial strains of *A. fumigatus*, and so whether or not the increased growth rate observed in this report is a consequence of life in space (i.e., mutation) or simply due to natural variation is not possible to determine without comparison to original terrestrial isolates. While a detailed analysis of specific SNPs in the ISS isolates was beyond the scope of our present study, the number total SNP differences of ISSFT-021 and IF1SW-F4 compared to Af293 were within the range of documented variance among existing sequences of environmental *A. fumigatus* strains (Fig. 1), suggesting that, without a more detailed analysis, the ISS strains likely reflect genetic diversity already found on Earth (22).

While it is conceivable that differential growth rates among *A. fumigatus* isolates (21) may favor more rapidly growing strains in gaining a niche foothold, the stresses aboard the ISS likely influence microbial colonization to a greater extent than *de novo* growth rates. As such, stress tolerance may play a more dominant role in the ability of *A. fumigatus* to colonize harsh niches. Given that the stress response pathways of *A. fumigatus* are unarguably involved in this organism’s environmental ubiquity and its leading position as an opportunistic pathogen (18, 45, 46), it is still unclear how existing genetic heterogeneity plays a role in stress tolerance. While limited in scope, we found no enhanced ability of the ISS strains over Af293 and CEA10 to tolerate a diversity of chemical stresses (Fig. 3), suggesting that stress tolerance may not play a dominant role in *A. fumigatus* persistence aboard the ISS. Future studies aimed at guiding enhanced disinfectant protocols aboard space vessels should consider these data and pursue effective methods for clearing surfaces of *A. fumigatus*. However, it was observed that both ISSFT-021 and IF1SW-F4 were significantly more resistant to UV irradiation than clinical isolates (26), and current studies are under way that will leverage sequence data and molecular approaches to elucidate these mechanisms. It is critical to note that care must be taken in interpreting these results, as terrestrial equivalents or reference strains, even if identified as the same species, do not represent experimental controls. Future studies aimed at determining the effects of microgravity and space irradiation on *A. fumigatus* biology will require direct comparison to terrestrial clones not subjected to time in space.

Examination of secondary metabolite profiles of clinical and ISS strains revealed differences in SM production (Fig. 4). Whether these changes are due to adaptations to unfavorable environmental conditions, such as low nutrient availability, enhanced irradiation, and microgravity, remains to be determined and will be the focus of future studies. One clear example linking genomic data to SM production was found with strain IF1SW-F4, with which increased levels of fumigaclavine A (47) were detected, whereas fumigaclavine C (48) production was significantly decreased (Fig. 4). The prenyl transferase FgaPT1 in *A. fumigatus* is known to be responsible for the prenylation of fumigaclavine A to form fumigaclavine C (49), and in the IF1SW-F4 strain there exists a frameshift mutation in *fgaPT1* (see Data Set S1 in the supplemental material) that might be responsible for the observed accumulation of fumigaclavine A. Additional differences in metabolite production between the two ISS strains compared to Af293 could be due to SNPs found within regions of the secondary metabolite gene clusters (see Data Set S1). Fumagillin, a toxic SM (50), is produced by both Af293 and CEA10, while production was significantly decreased in both of the ISS strains (Fig. 4). While fumagillin can alter neutrophil responses to pathogenic stimuli (51, 52), among other elements of host defense (17), it has been proposed as a potential virulence factor. Interestingly, the more virulent ISS strains showed lower fumagillin production, suggesting that *in vitro* fumagillin production profiles may not be accurate predictors of virulence potential, based on our *in vivo* findings with the neutrophil-deficient larval zebrafish model. Monomethylsulochrin, a proposed precursor in the trypacidin pathway, is produced by both ISS strains and Af293 but not by CEA10, despite the ability of this strain to produce trypacidin (36). However, our study was insufficient to pinpoint which, if any, of the metabolites has influence on the increased virulence observed in the two ISS strains, and additional experiments will be necessary to identify the connection between increased virulence and specific metabolite production.

As *A. fumigatus* is a ubiquitous environmental organism and multifaceted opportunistic pathogen (19), many studies have aimed at understanding traits underlying pathogenesis and whether a strain’s virulence potential can be inferred preemptively and indirectly from isolation source, physical traits, or genetic data. Interestingly, studies have been unable to consistently support the hypothesis that clinical strains have undergone a selective bottleneck and are therefore more virulent than environmental isolates (21, 22, 53). As both ISSFT-021 and IF1SW-F4 are environmental isolates, albeit from a highly unusual environment, our finding that both ISS strains are more virulent than the two clinical strains reinforces the idea that isolation source is not predictive of pathogenic potential and also reinforces the conclusion that all strains of *A. fumigatus*, regardless of origin, possess an infective potential (54). Alternatively, previous reports have suggested a correlation between *in vitro* growth rates and virulence (21, 55). While we did observe higher growth and germination rates of ISS isolates *in vitro* (Fig. 2), the greatest difference was the slower growth and germination of Af293 compared to all others, which may contribute to the lower virulence of this strain; however, the identical germination rates and slightly reduced radial growth of CEA10 are less suggestive of an underlying influence on virulence potential. Additionally, the growing body of literature characterizing *A. fumigatus* strain-dependent phenotypes has revealed broad phenotypes, including toxin production (54, 56) and host immune responses (27), that suggest a complex milieu of factors involved in predicting the outcome of host-*A. fumigatus* interactions. Furthermore, there has been speculation that mating type (MAT1-1, MAT1-2) may offer insight into the pathogenic potential of *A. fumigatus* isolates (53, 57), yet this supposition has been challenged by more recent studies using isogenic mating-type pairs (58). Both ISS strains are closely related (Fig. 1C; see also Fig. S1 in the supplemental material) but possess opposite mating types (Fig. 1C) and were found to be more virulent than either Af293 (MAT1-2) or CEA10 (MAT1-1) and equally virulent as each other (Fig. 5). Regardless of these strains coming from uncontrolled genetic backgrounds, our finding supports current data that suggest there is no link between mating type and virulence. Despite the ISS isolates coming from an extreme environment and possessing opposite mating types, different growth rates *in vitro*, and distinct SM profiles between themselves and Af293 and CEA10, our cumulative data are unable to offer a predictive potential of enhanced virulence for these strains.

This study has shown the existence of distinct *A. fumigatus* isolates on the ISS. The origin of these strains remains unknown, yet analysis of genome sequence data showed their relationship to 93 other sequenced genomes of this species and revealed a close relationship to a known patient isolate, Af300. Considering the genetic diversity and environmental ubiquity of *A. fumigatus*, it is not surprising to observe significant phenotypic variation among isolates, including growth rates, stress tolerance, SM production, and virulence, with virulence being a multifaceted phenotype in this opportunistic pathogen and likely a culmination of traits that render the pathogenic potential of any given isolate difficult to predict. Altogether, the present study reinforces the idea that all *A. fumigatus* strains, regardless of isolation source or genetic origin, represent potential pathogens and should serve to guide current and future sampling and maintenance regimens for space vessels.

## MATERIALS AND METHODS

### Isolation and verification of *A. fumigatus* isolates from the ISS.

Particulates associated with HEPA filters were scraped, and approximately 1 mg of material was resuspended in sterile phosphate-buffered saline (PBS; pH 7.4) before being spread onto potato dextrose agar (PDA) plates (7). To collect samples from cupola surfaces, sterile polyester wipes were used. The sampling wipes were assembled and manifested in the Jet Propulsion Laboratory (JPL, Pasadena, CA) prior to space flight. Briefly, each polyester wipe (9 by 9 in.; ITW Texwipe, Mahwah, NJ) was folded two times and soaked in 15 ml of sterile molecular-grade water (Sigma-Aldrich, St. Louis, MO) for 30 min, followed by transfer to a sterile zip lock bag (59). The wipes were packed along with the other kit elements at Ames Research Center (ARC; Moffett Field, CA) and included TC (total count, tryptic soy agar) and SDA (Sabouraud dextrose agar) contact slides (Hycon, EMD Millipore, Billerica, MA), Opsite adhesive tape (Smith & Nephew, Inc., London, United Kingdom), and an air sampling device with gelatin filters (Sartorius, Göttingen, Germany). Each sampling kit was sent to the ISS as a part of the Space-X cargo and was returned to Earth on Soyuz TM-14 or the Dragon capsule. The kits were delivered to JPL immediately after returning to Earth. During each sampling session on the ISS, only one astronaut collected samples from eight different locations, using wipes and contact slides. Each wipe was used to collect a sample 1 m^2^. The control wipe (environmental control) was only taken out from the zip lock bag, unfolded, and packed back into the zip lock. The samples were stored at 4°C until the return trip to Earth and subsequent processing.

The sample processing took place in a class 10K cleanroom at JPL immediately upon delivery of the return kits. Each wipe was aseptically taken out from the zip lock bag and transferred to a 500-ml sterile bottle containing 200 ml of sterile PBS. The bottle with the wipe was shaken for 2 min followed by concentration with a concentrating pipette (InnovaPrep, Drexel, MO) using 0.45-µm hollow fiber polysulfone tips and PBS elution fluid. The environmental control and each sample were concentrated to 4 ml. A 200-µl aliquot was serially diluted in PBS to estimate the cultivable population.

Concentrated samples were diluted in PBS (up to 10^−6^ of each original sample), plated on the media (100 µl; in duplicates) Reasoner’s 2A agar (R2A) for environmental bacteria and PDA for fungi, and incubated at 25°C for 7 days; CFU were then counted. A minimum of five isolates of distinct morphologies was picked up for each location from each type of medium. The isolates were archived in the semisolid R2A or PDA slants (agar media diluted 1:10) and stored at room temperature. For identification purposes, each fungal isolate was revived on PDA medium. Once a culture was confirmed to be pure, DNA extraction was performed by colony PCR (UltraClean DNA kit [Mo Bio, Carlsbad, CA] or Maxwell automated system [Promega, Madison, WI]). Concurrently, two cryobead stocks (Copán Diagnostics, Murrieta, CA) were prepared for each isolate. Fungal DNA was used for PCR to amplify ITS regions with primers ITS1F (5′ TTGGTCATTTAGAGGAAGTAA 3′) and Tw13 (5′ GGTCCGTGTTTCAAGACG 3′) (60) following an established protocol (61). The fungal sequences were searched against the UNITE database and identified based on the closest similarity to ITS sequences of fungal type strains (62).

Publicly available raw sequencing reads of all *A. fumigatus* strains used in this study were downloaded from either the NCBI Sequence Read Archive (SRA) or the EBI European Nucleotide Archive (ENA) for comparison to the ISS isolates. SNPs were called against the Af293 genome reference (NCBI accession number GCA_000002655.1) using the Snippy pipeline and default settings (63). Briefly, the Snippy program aligns reads to the genome reference sequence by using BWA v0.7.12-r1044 (64), and variants are called using the FreeBayes program v0.9.21-7-g7dd41db (65). The variants are then quality filtered, and a “core” set of SNP variants (defined as those SNPs with sufficient sequencing coverage for the genomic location for all isolates) are extracted from the data using Snippy. A custom python script (https://github.com/nextgenusfs/genome_scripts/blob/master/snippy2tree.py) was used to convert the SNP data to binary format, and a maximum likelihood phylogeny was inferred using RAxML v8.2.8 (66) with 1,000 bootstrap replicates. Snippy variant call files (VCF) from CEA10, ISSFT-021, and IF1SW-F4 were imported into CLC Genomics Workbench v9.01, and the variants were filtered and annotated based on overlap with secondary metabolite gene cluster predictions (34). To determine which variants would result in nonsynonymous changes to coding genes, the variants were annotated using the Amino Acid Changes module of CLC Genomics Workbench.

### Fungal culture.

After initial isolation on PDA plates, all *A. fumigatus* strains were grown on solid glucose minimal medium at 37°C, unless otherwise noted, for conidial preparation, physiological analysis, and stress tests. Conidial preparations for *in vitro* analyses were harvested after approximately 72 h with sterile 0.01% Tween–water and gentle agitation with an L-shaped spreader before being passed through a double layer of sterile mica cloth into a 50-ml screw-cap tube. Conidia were enumerated with a hemocytometer before adjustment to various concentrations as needed. Original spore suspensions maintained as glycerol stocks were stored at − 80°C.

### Physiological analysis.

All GMM plates for physiological analysis and stress testing were measured to contain 25 ml. For radial growth assessment, 1 × 10^4^ conidia in a volume of 10 µl were centrally inoculated onto GMM plates. Radial growth was measured daily at the time points indicated. For spore germination rate assays, 100 ml of liquid GMM was inoculated with 5 × 10^6^ spores/ml and grown at 37°C and 250 rpm. At 2, 4, 6, and 8 h, 1-ml samples were drawn, pelleted in a tabletop centrifuge for 1 min at maximum rpm and resuspended in a final volume of 100 µl to concentrate spores and germlings for facile enumeration. As germlings can clump, clouding clear counts, each sample was briefly sonicated in a water bath for 10 s. One hundred cells per condition were counted and scored. A spore was considered germinated when the germ tube diameter was greater than or equal to the diameter of the swollen base.

### Stress tests.

Radial growth was assayed as described above but after supplementation with various stressors. Concentrations for stress chemicals were as follows: 1 M NaCl, 4 µM menadione, 25 µg/ml Congo red, and 0.01% methyl methanesulfonate. For assessment of H_2_O_2_ sensitivity, it is easier to obtain consistent results by using a modified diffusion assay (33) over direct medium supplementation, as done for the stressors listed above. Briefly, spores were evenly suspended in 55°C GMM while still liquid at a final count 1.5 × 10^4^ per 25-ml plate. Following solidification of the agar, a 1-cm circular core was removed from the center of each plate, and the resulting hole was inoculated with 100 µl of 4% H_2_O_2_. Zones of inhibition were measured after 48 h.

### Secondary metabolite extraction and analysis.

Fungal isolates were cultivated at 30°C on GMM agar plates (6 g/liter NaNO_3_, 0.52 g/liter KCl, 0.52 g/liter MgSO_4_⋅7H_2_O, 1.52 g/liter KH_2_PO_4_, 10 g/liter d-glucose, 15 g/liter agar supplemented with 1 ml/liter of trace elements) at 10 × 10^6^ spores/µl per plate (10 cm). After 5 days, agar was chopped into small pieces and extracted with 25 ml methanol (MeOH), followed by 1-h sonication and filtration. Extraction and sonication steps were repeated with 25 ml of 1:1 MeOH-dichloromethane. After a second filtration, combined crude extracts of each isolate were evaporated *in vacuo* to yield a residue that was then suspended in 25 ml of water and partitioned with ethanol acetate (EtOAc; 25 ml). The EtOAc layer was evaporated *in vacuo*, redissolved in 2 ml of 20% dimethyl sulfoxide–MeOH, and 10 µl was examined by HPLC-DAD-MS analysis. HPLC-MS was carried out using a ThermoFinnigan LCQ Advantage ion trap mass spectrometer with a reverse-phase C_18_ column (3 μm; 2.1 by 100 μm; Alltech Prevail) at a flow rate of 125 µl/min. The solvent gradient for LC/MS was 95% MeCN–H_2_O (solvent B) in 5% MeCN–H_2_O (solvent A), both of which contained 0.05% formic acid, as follows: 0% solvent B from 0 to 5 min, 0 to 100% solvent B from 5 min to 35 min, 100% solvent B from 35 to 40 min, 100 to 0% solvent B from 40 to 45 min, and reequilibration with 0% solvent B from 45 to 50 min.

### Zebrafish care and maintenance.

Adult zebrafish were reared as described previously (39). Briefly, adults were maintained on a dedicated aquatic system and exposed to a light/dark cycle of 14 h and 10 h, respectively, and fed twice daily. After spawning, embryos were collected in E3 buffer and stored at 28.5°C. Methelyne blue, an ingredient of E3 buffer that inhibits fungal growth, was omitted from E3 buffer (E3-MB) at 24 h postfertilization. All larval zebrafish procedures and adult husbandry were performed in compliance with NIH guidelines and approved by the University of Wisconsin—Madison Institutional Animal Care and Use Committee.

### Larval zebrafish virulence assay.

Virulence assays were performed using the larval zebrafish model of invasive aspergillosis as described previously (39) with slight modifications. Larval immune suppression was obtained genetically through the use of transgenic *mpx:mCherry-2A-Rac2D57N* larvae (41). Prior to infection, larvae were screened and selected for mCherry expression in neutrophils to identify individuals harboring the dominant negative allele. Briefly, hindbrain ventricle infections were performed at 48-h postfertilization, versus 36 h postfertilization, as originally reported. Briefly, conidial stocks in PBS at a concentration of 1.5 × 10^8^ conidia/ml were mixed 2:1 with 1% phenol red to 1.0 × 10^8^ conidia/ml to visualize injection success. Larvae were anesthetized in E3-MB supplemented with 0.2 nM tricaine (ethyl 3-aminobenzoate; Sigma-Aldrich) prior to microinjection of 3 nl spore suspension or PBS vehicle control into the hindbrain ventricle through the otic vesicle. Following microinjection, larvae were rinsed several times to remove anesthetic and housed individually in wells of a 96-well plate in ~100 µl E3-MB. Survival of individual larvae was scored daily using loss of heartbeat as the readout for mortality.

### Statistical analysis.

In order to compare differences in SM production between isolates, the area under the electrospray ionization curve (ESI) was integrated for each compound. SM data collected from three independent biological replicates were used for statistical analysis. Ordinary one-way analysis of variance (ANOVA) was conducted to compare the level of production of seven identified SMs between Af293 (treated as a control) and CEA10, ISSFT-021, and IF1SW-F4. The data are presented as column charts with corresponding error bars. Data analysis was conducted using GraphPad Prism version 7. Survival analysis for larval zebrafish infection experiments was performed as previously described (39) by pooling experimental replicates and generating *P* values via Cox proportional hazards regression analysis.

### Accession number(s).

The ITS sequence for IF1SW-F4 is available under GenBank accession number KX675260.
