# Supplementary figures and images for: Characterization of Aspergillus fumigatus Isolates from Air and Surfaces of the International Space Station
Source: mSphere. 2016 Oct 26;1(5):e00227-16. doi: 10.1128/mSphere.00227-16 (PMC5082629; doi:10.1128/mSphere.00227-16)

CEA10

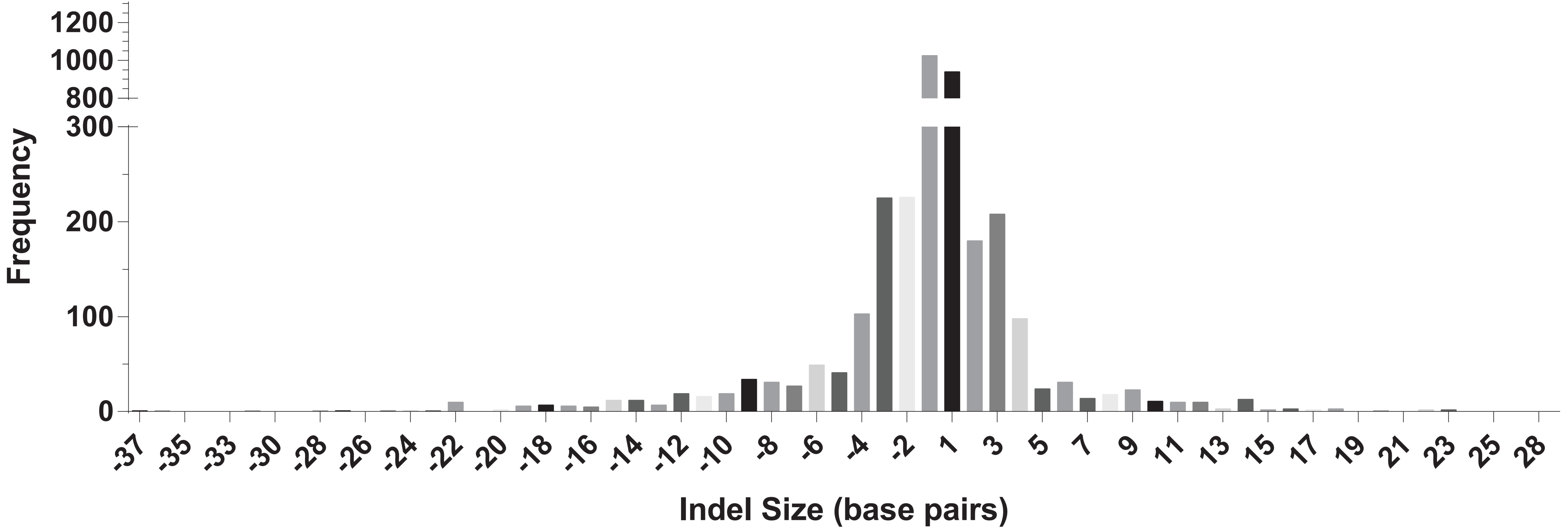

ISSFT-021

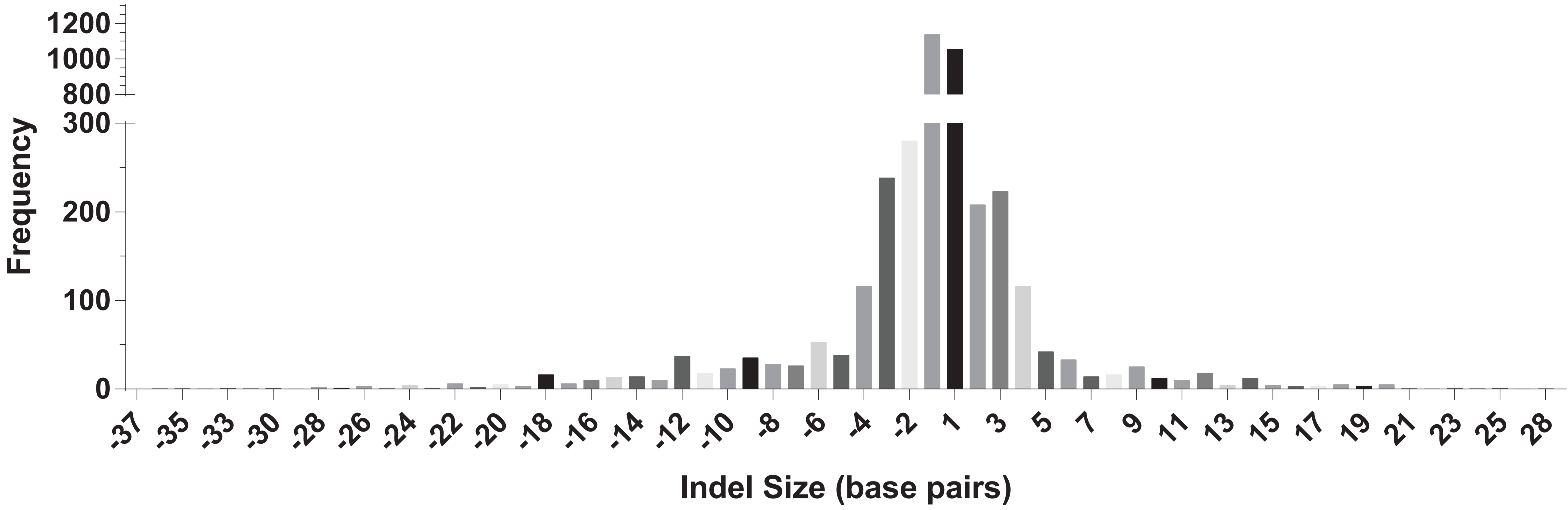

IF1SW-F4

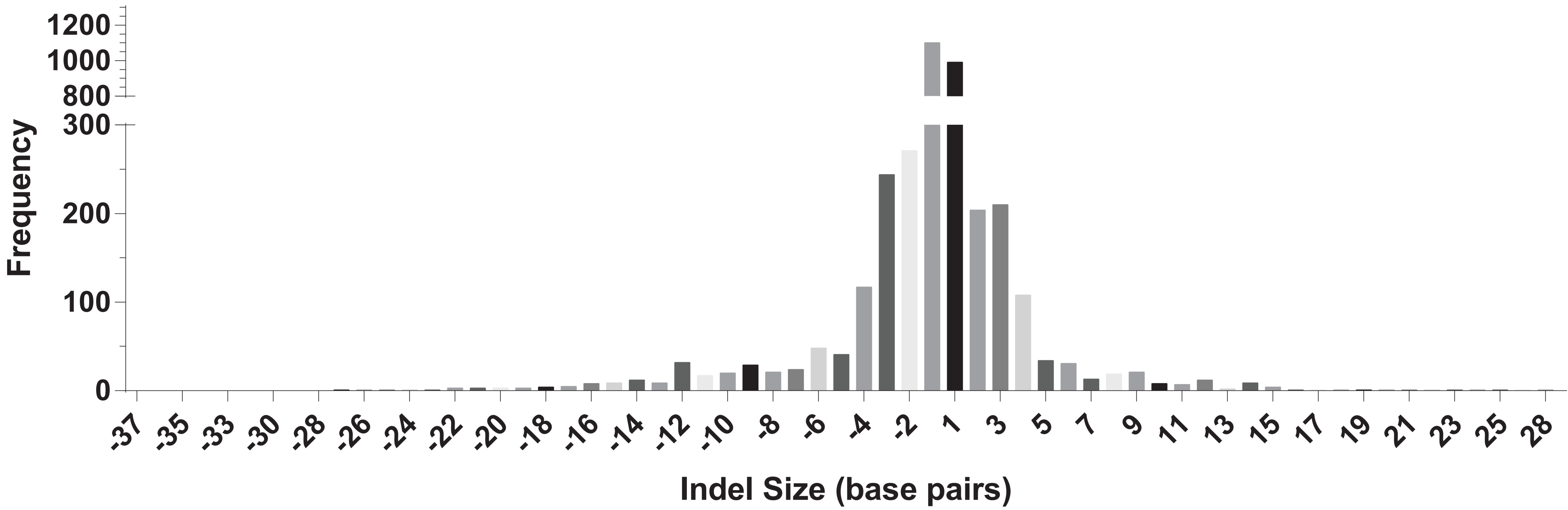

Average - All Strains (94 Total)

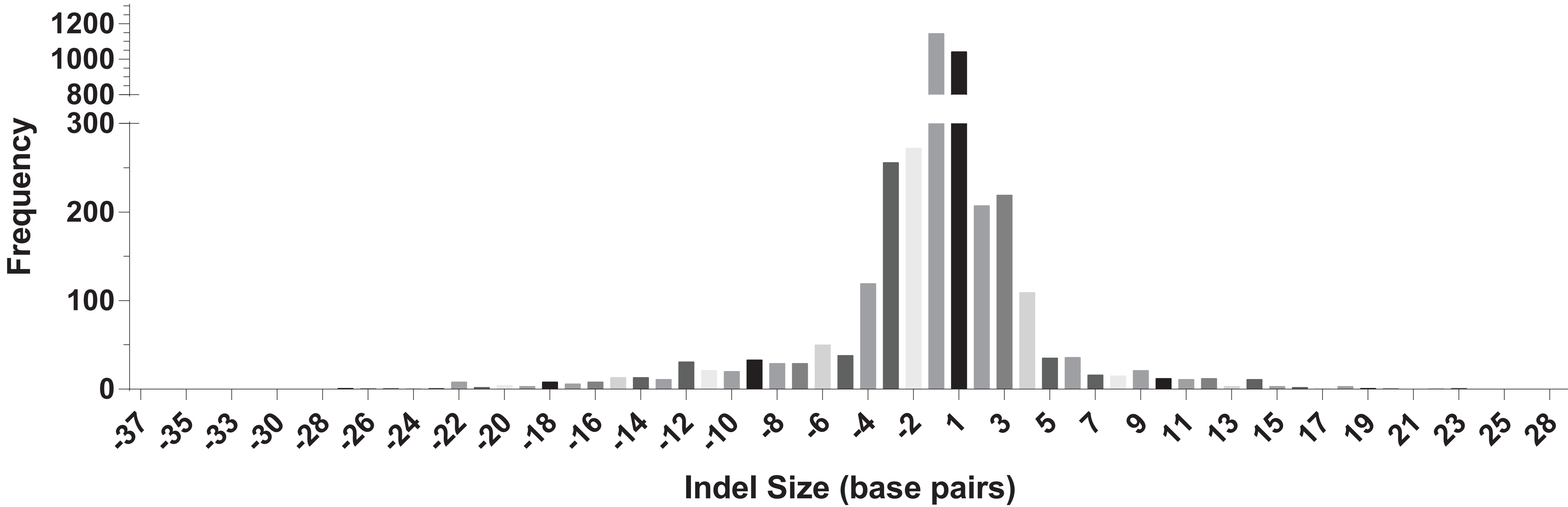

Supplement: Figure S2 [file sph005162172sf2.pdf]

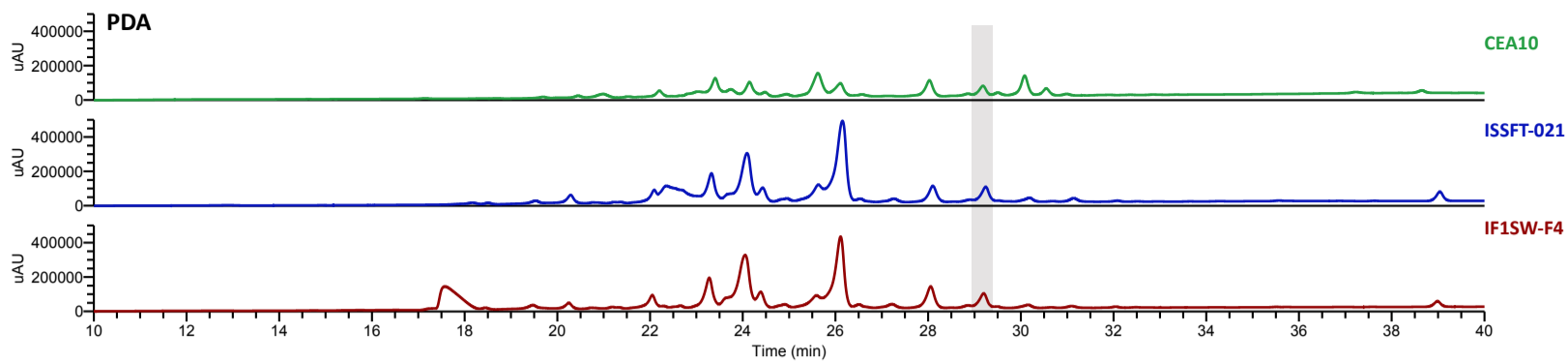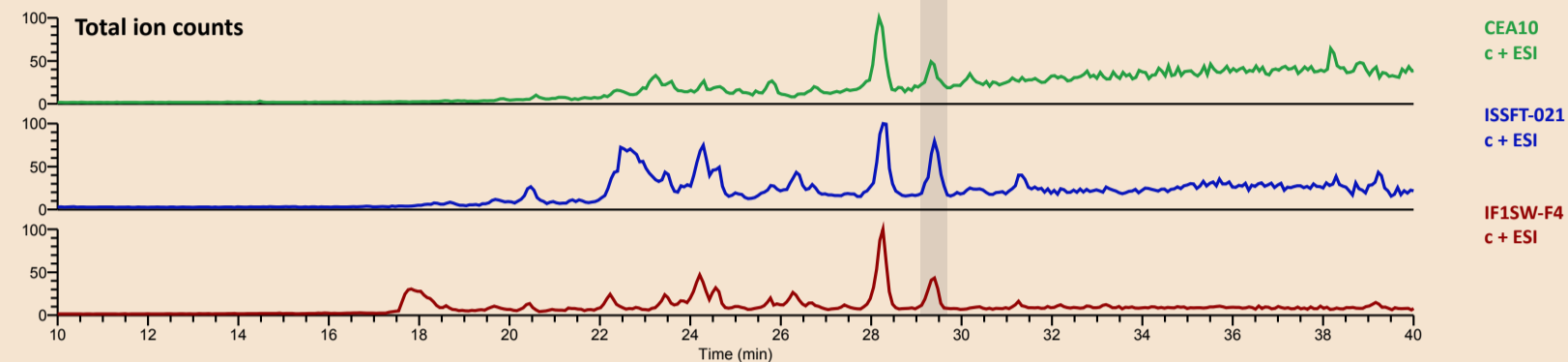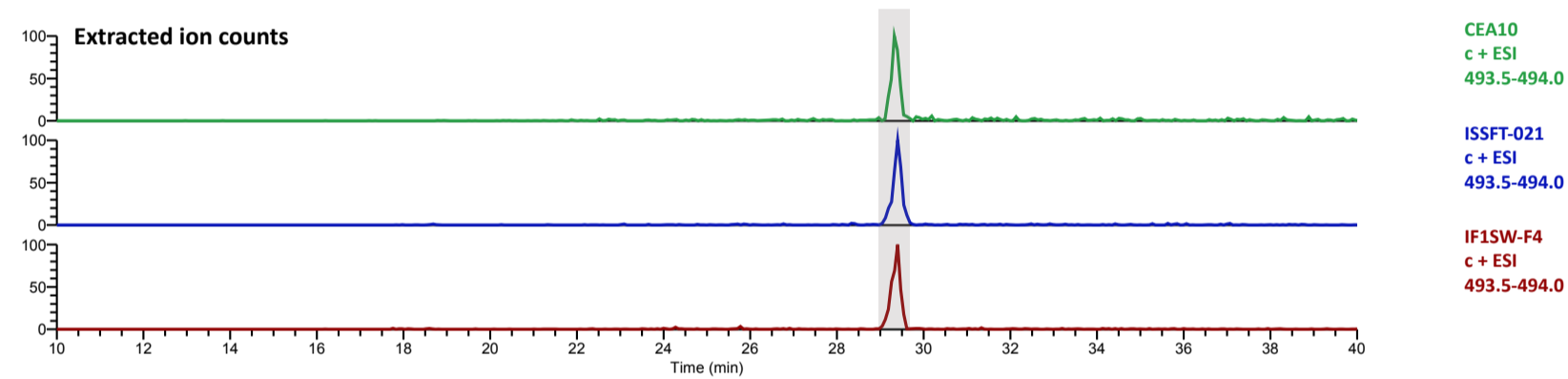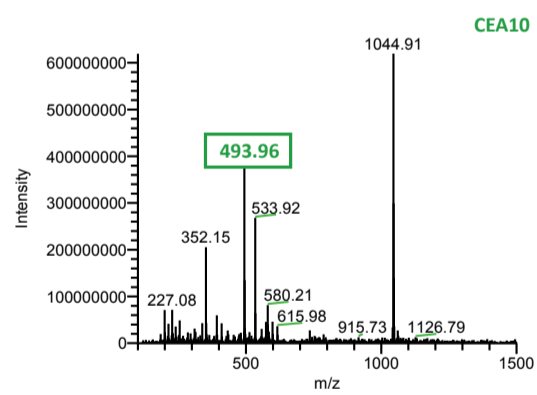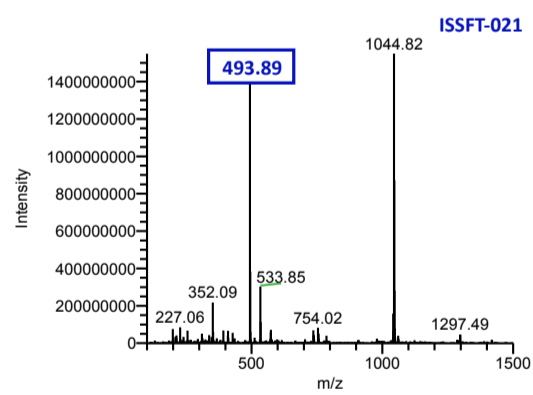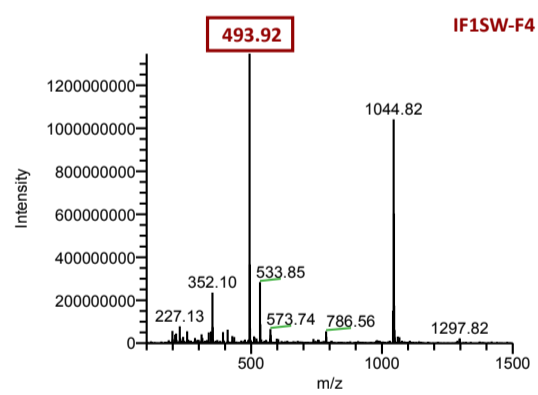

Supplement: Figure S3 [file sph005162172sf3.pdf]

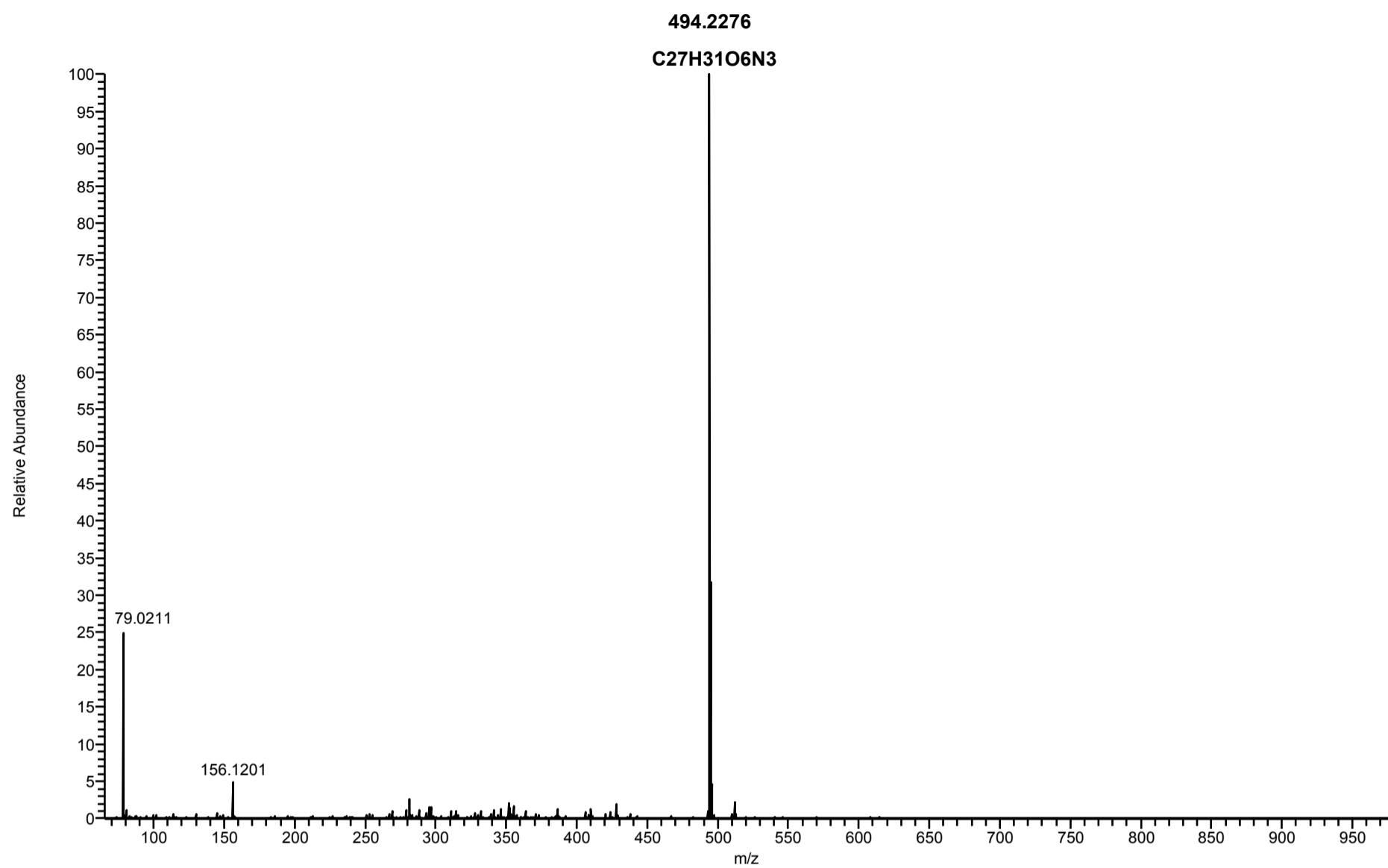

Supplement: Figure S4 [file sph005162172sf4.pdf]
