# Supplementary material for: Characterization of Aspergillus fumigatus Isolates from Air and Surfaces of the International Space Station
Source: mSphere. 2016 Oct 26;1(5):e00227-16. doi: 10.1128/mSphere.00227-16 (PMC5082629; doi:10.1128/mSphere.00227-16)
Supplement: Data Set S1 [file sph005162172sd5.pdf]

Chromosome	Region	Type	Reference	Allele	Length	Isolates harboring variant	Coding region change	Amino acid change	Non-synonymous	Af293 SecMet Cluster
Chr1	2646160	SNV	C	T	1	CEA10	Afu1g10270:c.298C>T	Afu1g10270:p.Arg100Cys	Yes	Cluster_2_nidulanin
Chr1	2646848	SNV	C	T	1	CEA10, ISSFT-021, IF1SW-F4	Afu1g10270:c.925C>T	Afu1g10270:p.Arg309Trp	Yes	Cluster_2_nidulanin
Chr1	2648583	SNV	G	C	1	CEA10, ISSFT-021, IF1SW-F4	Afu1g10280:c.1047G>C		No	Cluster_2_nidulanin
Chr1	2652911	SNV	A	C	1	CEA10	Afu1g10295:c.1737T>G		No	Cluster_2_nidulanin
Chr1	2653424	SNV	G	A	1	ISSFT-021, IF1SW-F4	Afu1g10295:c.1224C>T		No	Cluster_2_nidulanin
Chr1	2654215	SNV	A	G	1	CEA10, ISSFT-021, IF1SW-F4	Afu1g10295:c.433T>C	Afu1g10295:p.Ser145Pro	Yes	Cluster_2_nidulanin
Chr1	2655545	SNV	T	C	1	CEA10, ISSFT-021, IF1SW-F4	Afu1g10310:c.-99T>C		-	Cluster_2_nidulanin
Chr1	2655583	SNV	G	A	1	CEA10, ISSFT-021, IF1SW-F4	Afu1g10310:c.-61G>A		-	Cluster_2_nidulanin
Chr1	2655889	SNV	A	G	1	CEA10, ISSFT-021, IF1SW-F4	Afu1g10310:c.58-17A>G		-	Cluster_2_nidulanin
Chr1	2661626	SNV	A	C	1	CEA10, ISSFT-021, IF1SW-F4	Afu1g10330:c.-121A>C		-	Cluster_2_nidulanin
Chr1	2663148	SNV	C	T	1	CEA10, ISSFT-021, IF1SW-F4	Afu1g10330:c.1284C>T		No	Cluster_2_nidulanin
Chr1	2663743	SNV	A	G	1	CEA10, ISSFT-021, IF1SW-F4	Afu1g10330:c.*69A>G		-	Cluster_2_nidulanin
Chr1	2664079	SNV	G	T	1	CEA10, ISSFT-021, IF1SW-F4	Afu1g10340:c.-278G>T		-	Cluster_2_nidulanin
Chr1	2664318	SNV	G	A	1	CEA10, ISSFT-021, IF1SW-F4	Afu1g10340:c.-39G>A		-	Cluster_2_nidulanin
Chr1	2665344	SNV	T	C	1	CEA10, ISSFT-021, IF1SW-F4	Afu1g10340:c.657T>C		No	Cluster_2_nidulanin
Chr1	2665532	SNV	C	T	1	CEA10, ISSFT-021, IF1SW-F4	Afu1g10340:c.*24C>T		-	Cluster_2_nidulanin
Chr1	2665674	SNV	C	T	1	CEA10, ISSFT-021, IF1SW-F4	Afu1g10340:c.*166C>T		-	Cluster_2_nidulanin
Chr1	2666193	SNV	C	T	1	CEA10, ISSFT-021, IF1SW-F4			-	Cluster_2_nidulanin
Chr1	2667564	SNV	C	T	1	ISSFT-021	Afu1g10350:c.1149C>T		No	Cluster_2_nidulanin
Chr1	2668320	SNV	A	G	1	CEA10, ISSFT-021, IF1SW-F4	Afu1g10355:c.218+64T>C		-	Cluster_2_nidulanin
Chr1	2668789	SNV	T	C	1	CEA10, ISSFT-021, IF1SW-F4			-	Cluster_2_nidulanin
Chr1	2670708	SNV	A	G	1	CEA10, ISSFT-021, IF1SW-F4	Afu1g10360:c.713A>G	Afu1g10360:p.Asn238Ser	Yes	Cluster_2_nidulanin
Chr1	2671472	SNV	C	T	1	CEA10, ISSFT-021, IF1SW-F4	Afu1g10360:c.1421C>T	Afu1g10360:p.Ala474Val	Yes	Cluster_2_nidulanin
Chr1	2673100	SNV	T	G	1	CEA10			-	Cluster_2_nidulanin
Chr1	2673870..2673871	MNV	GG	AA	2	IF1SW-F4	Afu1g10370:c.454_455delGGinsAA	Afu1g10370:p.Gly152Lys	Yes	Cluster_2_nidulanin
Chr1	2674138	SNV	C	T	1	CEA10, ISSFT-021, IF1SW-F4	Afu1g10370:c.663C>T		No	Cluster_2_nidulanin
Chr1	2674253	SNV	G	A	1	CEA10, ISSFT-021, IF1SW-F4	Afu1g10370:c.778G>A	Afu1g10370:p.Val260Ile	Yes	Cluster_2_nidulanin
Chr1	2674435	SNV	G	A	1	ISSFT-021	Afu1g10370:c.903G>A		No	Cluster_2_nidulanin
Chr1	2674978	SNV	C	T	1	CEA10, ISSFT-021, IF1SW-F4	Afu1g10370:c.1446C>T		No	Cluster_2_nidulanin
Chr1	2675424..2675426	MNV	ATT	CTA	3	CEA10, ISSFT-021, IF1SW-F4			-	Cluster_2_nidulanin
Chr1	2677924	SNV	T	C	1	CEA10, ISSFT-021, IF1SW-F4	Afu1g10380:c.16519A>G	Afu1g10380:p.Ile5507Val	Yes	Cluster_2_nidulanin
Chr1	2678159	SNV	G	A	1	ISSFT-021	Afu1g10380:c.16284C>T		No	Cluster_2_nidulanin
Chr1	2683960	SNV	G	C	1	CEA10, ISSFT-021, IF1SW-F4	Afu1g10380:c.10703C>G	Afu1g10380:p.Ala3568Gly	Yes	Cluster_2_nidulanin
Chr1	2685168	SNV	T	C	1	CEA10, ISSFT-021, IF1SW-F4	Afu1g10380:c.9495A>G		No	Cluster_2_nidulanin
Chr1	2686541	SNV	A	G	1	CEA10, ISSFT-021, IF1SW-F4	Afu1g10380:c.8122T>C	Afu1g10380:p.Phe2708Leu	Yes	Cluster_2_nidulanin
Chr1	2688346	SNV	A	G	1	CEA10, ISSFT-021, IF1SW-F4	Afu1g10380:c.6317T>C	Afu1g10380:p.Leu2106Pro	Yes	Cluster_2_nidulanin
Chr1	2688395	SNV	T	C	1	CEA10, ISSFT-021, IF1SW-F4	Afu1g10380:c.6268A>G	Afu1g10380:p.Thr2090Ala	Yes	Cluster_2_nidulanin
Chr1	2688896	SNV	T	C	1	CEA10, ISSFT-021, IF1SW-F4	Afu1g10380:c.5767A>G	Afu1g10380:p.Met1923Val	Yes	Cluster_2_nidulanin
Chr1	2690799	SNV	G	A	1	CEA10, ISSFT-021, IF1SW-F4	Afu1g10380:c.3864C>T		No	Cluster_2_nidulanin
Chr1	2690845	SNV	A	T	1	CEA10, ISSFT-021, IF1SW-F4	Afu1g10380:c.3818T>A	Afu1g10380:p.Phe1273Tyr	Yes	Cluster_2_nidulanin
Chr1	2692074	SNV	A	G	1	CEA10, ISSFT-021, IF1SW-F4	Afu1g10380:c.2589T>C		No	Cluster_2_nidulanin
Chr1	2694898	SNV	A	G	1	CEA10, ISSFT-021, IF1SW-F4	Afu1g10380:c.-11T>C		-	Cluster_2_nidulanin
Chr1	2695677	SNV	T	C	1	CEA10, ISSFT-021, IF1SW-F4			-	Cluster_2_nidulanin
Chr1	2696408	SNV	A	T	1	CEA10, ISSFT-021, IF1SW-F4			-	Cluster_2_nidulanin
Chr1	2696435	Deletion	A	-	1	CEA10, IF1SW-F4			-	Cluster_2_nidulanin
Chr1	2697235	SNV	C	T	1	CEA10, ISSFT-021, IF1SW-F4			-	Cluster_2_nidulanin
Chr1	2698291	SNV	A	G	1	CEA10, ISSFT-021, IF1SW-F4			-	Cluster_2_nidulanin
Chr1	2698348	SNV	G	A	1	CEA10, ISSFT-021, IF1SW-F4			-	Cluster_2_nidulanin
Chr1	2698540	SNV	G	A	1	CEA10, ISSFT-021, IF1SW-F4			-	Cluster_2_nidulanin
Chr1	2699197	SNV	G	T	1	CEA10, ISSFT-021, IF1SW-F4			-	Cluster_2_nidulanin
Chr1	2699453	SNV	A	C	1	CEA10, ISSFT-021, IF1SW-F4			-	Cluster_2_nidulanin
Chr1	2699572	SNV	G	A	1	CEA10, ISSFT-021, IF1SW-F4			-	Cluster_2_nidulanin
Chr1	2700073	SNV	C	A	1	CEA10, ISSFT-021, IF1SW-F4	Afu1g10390:c.-299C>A		-	Cluster_2_nidulanin
Chr1	2704467	SNV	T	C	1	CEA10, ISSFT-021, IF1SW-F4	Afu1g10390:c.3387T>C		No	Cluster_2_nidulanin
Chr1	4685128	SNV	T	C	1	CEA10, ISSFT-021, IF1SW-F4	Afu1g17190:c.1734A>G		No	Cluster_3_ferricrocin
Chr1	4687418	SNV	C	T	1	ISSFT-021, IF1SW-F4			-	Cluster_3_ferricrocin
Chr1	4687522	SNV	T	C	1	ISSFT-021, IF1SW-F4			-	Cluster_3_ferricrocin

Chr1	4687762	SNV	C	T	1	ISSFT-021, IF1SW-F4			-	Cluster_3_ferricrocin
Chr1	4687882	SNV	G	A	1	ISSFT-021, IF1SW-F4			-	Cluster_3_ferricrocin
Chr1	4687970	SNV	G	A	1	ISSFT-021, IF1SW-F4			-	Cluster_3_ferricrocin
Chr1	4687987	SNV	T	G	1	ISSFT-021, IF1SW-F4			-	Cluster_3_ferricrocin
Chr1	4688032	SNV	A	C	1	ISSFT-021, IF1SW-F4			-	Cluster_3_ferricrocin
Chr1	4688042..4688044	MNV	TTT	CTC	3	ISSFT-021, IF1SW-F4			-	Cluster_3_ferricrocin
Chr1	4688086	SNV	C	T	1	ISSFT-021, IF1SW-F4			-	Cluster_3_ferricrocin
Chr1	4688229^4688230	Insertion	-	A	1	ISSFT-021, IF1SW-F4			-	Cluster_3_ferricrocin
Chr1	4688273	SNV	C	T	1	ISSFT-021, IF1SW-F4			-	Cluster_3_ferricrocin
Chr1	4688387	SNV	T	A	1	ISSFT-021, IF1SW-F4			-	Cluster_3_ferricrocin
Chr1	4688541	SNV	G	T	1	ISSFT-021, IF1SW-F4			-	Cluster_3_ferricrocin
Chr1	4690630	SNV	T	C	1	CEA10	Afu1g17200:c.1781T>C	Afu1g17200:p.Leu594Ser	Yes	Cluster_3_ferricrocin
Chr1	4692240	SNV	A	G	1	CEA10, ISSFT-021, IF1SW-F4	Afu1g17200:c.3391A>G	Afu1g17200:p.Asn1131Asp	Yes	Cluster_3_ferricrocin
Chr1	4692715	SNV	A	G	1	ISSFT-021, IF1SW-F4	Afu1g17200:c.3866A>G	Afu1g17200:p.His1289Arg	Yes	Cluster_3_ferricrocin
Chr1	4693587	SNV	T	C	1	ISSFT-021, IF1SW-F4	Afu1g17200:c.4738T>C	Afu1g17200:p.Trp1580Arg	Yes	Cluster_3_ferricrocin
Chr1	4693620	SNV	C	A	1	CEA10	Afu1g17200:c.4771C>A	Afu1g17200:p.Leu1591Ile	Yes	Cluster_3_ferricrocin
Chr1	4694243	SNV	G	A	1	ISSFT-021, IF1SW-F4	Afu1g17200:c.5394G>A		No	Cluster_3_ferricrocin
Chr1	4694600	SNV	A	G	1	CEA10, ISSFT-021, IF1SW-F4	Afu1g17200:c.5751A>G		No	Cluster_3_ferricrocin
Chr1	4694647	SNV	T	C	1	CEA10	Afu1g17200:c.5798T>C	Afu1g17200:p.Met1933Thr	Yes	Cluster_3_ferricrocin
Chr1	4698447	SNV	G	A	1	CEA10, ISSFT-021, IF1SW-F4	Afu1g17200:c.9598G>A	Afu1g17200:p.Gly3200Ser	Yes	Cluster_3_ferricrocin
Chr1	4698576	SNV	T	C	1	CEA10, ISSFT-021, IF1SW-F4	Afu1g17200:c.9727T>C	Afu1g17200:p.Phe3243Leu	Yes	Cluster_3_ferricrocin
Chr1	4699005	SNV	G	A	1	ISSFT-021, IF1SW-F4	Afu1g17200:c.10156G>A	Afu1g17200:p.Gly3386Ser	Yes	Cluster_3_ferricrocin
Chr1	4700709	SNV	G	A	1	ISSFT-021, IF1SW-F4	Afu1g17200:c.11860G>A	Afu1g17200:p.Glu3954Lys	Yes	Cluster_3_ferricrocin
Chr1	4700784	SNV	T	G	1	CEA10, ISSFT-021, IF1SW-F4	Afu1g17200:c.11935T>G	Afu1g17200:p.Ser3979Ala	Yes	Cluster_3_ferricrocin
Chr1	4702148	SNV	C	T	1	CEA10	Afu1g17200:c.13299C>T		No	Cluster_3_ferricrocin
Chr1	4702460	SNV	A	T	1	ISSFT-021, IF1SW-F4	Afu1g17200:c.13611A>T		No	Cluster_3_ferricrocin
Chr1	4703071	SNV	G	A	1	CEA10, ISSFT-021, IF1SW-F4	Afu1g17200:c.14222G>A	Afu1g17200:p.Gly4741Glu	Yes	Cluster_3_ferricrocin
Chr1	4895974	SNV	G	A	1	ISSFT-021, IF1SW-F4			-	Cluster_4_fusarielin-like
Chr1	4896854	SNV	C	T	1	ISSFT-021, IF1SW-F4			-	Cluster_4_fusarielin-like
Chr1	4897961	SNV	A	C	1	ISSFT-021, IF1SW-F4	Afu1g17723:c.232T>G	Afu1g17723:p.Tyr78Asp	Yes	Cluster_4_fusarielin-like
Chr1	4898209	SNV	A	C	1	ISSFT-021, IF1SW-F4			-	Cluster_4_fusarielin-like
Chr1	4898770	SNV	A	C	1	ISSFT-021, IF1SW-F4	Afu1g17725:c.1272T>G		No	Cluster_4_fusarielin-like
Chr1	4900748	SNV	C	T	1	ISSFT-021, IF1SW-F4	Afu1g17730:c.953G>A	Afu1g17730:p.Arg318Lys	Yes	Cluster_4_fusarielin-like
Chr1	4901481..4901482	MNV	AA	CC	2	ISSFT-021, IF1SW-F4	Afu1g17730:c.282_283delTTinsGG	Afu1g17730:p.Trp95Gly	Yes	Cluster_4_fusarielin-like
Chr1	4902271	SNV	C	G	1	ISSFT-021, IF1SW-F4			-	Cluster_4_fusarielin-like
Chr1	4903444	SNV	C	A	1	ISSFT-021, IF1SW-F4	Afu1g17740:c.1058C>A	Afu1g17740:p.Ala353Asp	Yes	Cluster_4_fusarielin-like
Chr1	4903980	SNV	A	G	1	ISSFT-021, IF1SW-F4	Afu1g17740:c.1515A>G		No	Cluster_4_fusarielin-like
Chr1	4904010	SNV	T	A	1	ISSFT-021, IF1SW-F4	Afu1g17740:c.1545T>A		No	Cluster_4_fusarielin-like
Chr1	4904591	SNV	G	C	1	ISSFT-021, IF1SW-F4	Afu1g17740:c.2035G>C	Afu1g17740:p.Glu679Gln	Yes	Cluster_4_fusarielin-like
Chr1	4904937	SNV	C	A	1	ISSFT-021, IF1SW-F4	Afu1g17740:c.2381C>A	Afu1g17740:p.Ala794Asp	Yes	Cluster_4_fusarielin-like
Chr1	4905466	SNV	A	G	1	ISSFT-021, IF1SW-F4	Afu1g17740:c.2778A>G		No	Cluster_4_fusarielin-like
Chr1	4905500	SNV	T	C	1	ISSFT-021, IF1SW-F4	Afu1g17740:c.2812T>C	Afu1g17740:p.Trp938Arg	Yes	Cluster_4_fusarielin-like
Chr1	4905956	SNV	A	G	1	ISSFT-021, IF1SW-F4	Afu1g17740:c.3217-24A>G		-	Cluster_4_fusarielin-like
Chr1	4907058	SNV	G	C	1	ISSFT-021, IF1SW-F4	Afu1g17740:c.4260G>C		No	Cluster_4_fusarielin-like
Chr1	4907995	SNV	A	G	1	ISSFT-021, IF1SW-F4	Afu1g17740:c.5197A>G	Afu1g17740:p.Asn1733Asp	Yes	Cluster_4_fusarielin-like
Chr1	4908205	SNV	G	A	1	ISSFT-021, IF1SW-F4	Afu1g17740:c.5311G>A	Afu1g17740:p.Ala1771Thr	Yes	Cluster_4_fusarielin-like
Chr1	4909348	SNV	G	C	1	ISSFT-021, IF1SW-F4	Afu1g17740:c.6340G>C	Afu1g17740:p.Asp2114His	Yes	Cluster_4_fusarielin-like
Chr2	4678781	SNV	G	C	1	CEA10, ISSFT-021, IF1SW-F4	Afu2g17530:c.1068G>C		No	Cluster_5_DHN-melanin
Chr2	4679096	SNV	T	C	1	CEA10, ISSFT-021, IF1SW-F4	Afu2g17530:c.1383T>C		No	Cluster_5_DHN-melanin
Chr2	4679497	SNV	A	C	1	CEA10, ISSFT-021, IF1SW-F4			-	Cluster_5_DHN-melanin
Chr2	4679585	SNV	A	G	1	CEA10, ISSFT-021, IF1SW-F4			-	Cluster_5_DHN-melanin
Chr2	4679602	SNV	T	C	1	CEA10, ISSFT-021, IF1SW-F4			-	Cluster_5_DHN-melanin
Chr2	4679624	SNV	A	G	1	CEA10, ISSFT-021, IF1SW-F4			-	Cluster_5_DHN-melanin
Chr2	4679692	SNV	G	T	1	CEA10, ISSFT-021, IF1SW-F4			-	Cluster_5_DHN-melanin
Chr2	4679728	SNV	A	G	1	IF1SW-F4			-	Cluster_5_DHN-melanin
Chr2	4679731	SNV	C	T	1	ISSFT-021			-	Cluster_5_DHN-melanin
Chr2	4679780	SNV	C	G	1	CEA10, ISSFT-021, IF1SW-F4			-	Cluster_5_DHN-melanin
Chr2	4679902..4679906	MNV	TCTTT	CCTTC	5	CEA10, ISSFT-021, IF1SW-F4			-	Cluster_5_DHN-melanin
Chr2	4679917	SNV	C	G	1	CEA10, ISSFT-021, IF1SW-F4			-	Cluster_5_DHN-melanin

Chr2	4680328	SNV	C	T	1	CEA10, ISSFT-021, IF1SW-F4	Afu2g17540:c.333+13C>T		-	Cluster_5_DHN-melanin
Chr2	4680337	SNV	C	G	1	CEA10, ISSFT-021, IF1SW-F4	Afu2g17540:c.333+22C>G		-	Cluster_5_DHN-melanin
Chr2	4680412	SNV	C	A	1	CEA10, ISSFT-021, IF1SW-F4	Afu2g17540:c.378C>A		No	Cluster_5_DHN-melanin
Chr2	4680766	SNV	T	C	1	CEA10, ISSFT-021	Afu2g17540:c.732T>C		No	Cluster_5_DHN-melanin
Chr2	4681214	SNV	T	A	1	CEA10, ISSFT-021, IF1SW-F4	Afu2g17540:c.1180T>A	Afu2g17540:p.Leu394Ile	Yes	Cluster_5_DHN-melanin
Chr2	4681330	SNV	T	C	1	CEA10, ISSFT-021, IF1SW-F4	Afu2g17540:c.1296T>C		No	Cluster_5_DHN-melanin
Chr2	4681661	SNV	G	A	1	CEA10, ISSFT-021, IF1SW-F4	Afu2g17540:c.1580G>A	Afu2g17540:p.Gly527Asp	Yes	Cluster_5_DHN-melanin
Chr2	4681711	SNV	G	A	1	CEA10, ISSFT-021, IF1SW-F4	Afu2g17540:c.1630G>A	Afu2g17540:p.Gly544Ser	Yes	Cluster_5_DHN-melanin
Chr2	4681815	SNV	T	C	1	CEA10, ISSFT-021, IF1SW-F4	Afu2g17540:c.1734T>C		No	Cluster_5_DHN-melanin
Chr2	4681896	SNV	G	A	1	CEA10, ISSFT-021, IF1SW-F4	Afu2g17540:c.1815G>A		No	Cluster_5_DHN-melanin
Chr2	4682218^4682219	Insertion	-	G	1	CEA10, ISSFT-021, IF1SW-F4			-	Cluster_5_DHN-melanin
Chr2	4682226..4682228	Deletion	CCT	-	3	CEA10, ISSFT-021, IF1SW-F4			-	Cluster_5_DHN-melanin
Chr2	4682240	SNV	C	T	1	CEA10, ISSFT-021, IF1SW-F4			-	Cluster_5_DHN-melanin
Chr2	4682472	SNV	A	G	1	CEA10, ISSFT-021, IF1SW-F4			-	Cluster_5_DHN-melanin
Chr2	4682778	SNV	C	T	1	CEA10, ISSFT-021, IF1SW-F4	Afu2g17550:c.996G>A		No	Cluster_5_DHN-melanin
Chr2	4683166	SNV	A	G	1	CEA10, ISSFT-021, IF1SW-F4	Afu2g17550:c.657T>C		No	Cluster_5_DHN-melanin
Chr2	4683232	SNV	T	G	1	CEA10, ISSFT-021, IF1SW-F4	Afu2g17550:c.591A>C		No	Cluster_5_DHN-melanin
Chr2	4683249	SNV	A	G	1	CEA10, ISSFT-021, IF1SW-F4	Afu2g17550:c.574T>C	Afu2g17550:p.Ser192Pro	Yes	Cluster_5_DHN-melanin
Chr2	4683381	SNV	T	C	1	CEA10, ISSFT-021, IF1SW-F4	Afu2g17550:c.442A>G	Afu2g17550:p.Thr148Ala	Yes	Cluster_5_DHN-melanin
Chr2	4683467	SNV	C	T	1	CEA10, ISSFT-021, IF1SW-F4	Afu2g17550:c.356G>A	Afu2g17550:p.Ser119Asn	Yes	Cluster_5_DHN-melanin
Chr2	4683631	SNV	C	T	1	CEA10, ISSFT-021, IF1SW-F4	Afu2g17550:c.192G>A		No	Cluster_5_DHN-melanin
Chr2	4683643	SNV	G	A	1	CEA10, ISSFT-021, IF1SW-F4	Afu2g17550:c.180C>T		No	Cluster_5_DHN-melanin
Chr2	4683828	SNV	G	A	1	CEA10, ISSFT-021, IF1SW-F4	Afu2g17550:c.89C>T	Afu2g17550:p.Ser30Phe	Yes	Cluster_5_DHN-melanin
Chr2	4683908	SNV	G	T	1	CEA10, ISSFT-021, IF1SW-F4	Afu2g17550:c.9C>A		No	Cluster_5_DHN-melanin
Chr2	4684025	SNV	T	C	1	CEA10, ISSFT-021, IF1SW-F4			-	Cluster_5_DHN-melanin
Chr2	4684154	SNV	C	T	1	CEA10, ISSFT-021, IF1SW-F4			-	Cluster_5_DHN-melanin
Chr2	4684266	SNV	G	C	1	CEA10, ISSFT-021, IF1SW-F4			-	Cluster_5_DHN-melanin
Chr2	4686802	SNV	G	T	1	ISSFT-021			-	Cluster_5_DHN-melanin
Chr2	4687406	SNV	G	A	1	CEA10			-	Cluster_5_DHN-melanin
Chr2	4688349	SNV	A	G	1	CEA10, ISSFT-021, IF1SW-F4	Afu2g17600:c.559A>G	Afu2g17600:p.Asn187Asp	Yes	Cluster_5_DHN-melanin
Chr2	4689670	SNV	G	A	1	ISSFT-021	Afu2g17600:c.1833G>A		No	Cluster_5_DHN-melanin
Chr2	4692702	SNV	T	A	1	ISSFT-021	Afu2g17600:c.4765-27T>A		-	Cluster_5_DHN-melanin
Chr2	4693591	SNV	C	T	1	IF1SW-F4	Afu2g17600:c.5627C>T	Afu2g17600:p.Pro1876Leu	Yes	Cluster_5_DHN-melanin
Chr2	4776978	SNV	G	A	1	CEA10	Afu2g17960:c.1092C>T		No	Cluster_6_fumigaclavine
Chr2	4777232	SNV	C	T	1	ISSFT-021	Afu2g17960:c.838G>A	Afu2g17960:p.Glu280Lys	Yes	Cluster_6_fumigaclavine
Chr2	4777445	SNV	C	A	1	CEA10	Afu2g17960:c.625G>T	Afu2g17960:p.Val209Leu	Yes	Cluster_6_fumigaclavine
Chr2	4778240..4778244	MNV	CGGGC	TGGGT	5	ISSFT-021, IF1SW-F4			-	Cluster_6_fumigaclavine
Chr2	4779905	SNV	A	G	1	ISSFT-021, IF1SW-F4			-	Cluster_6_fumigaclavine
Chr2	4780224	SNV	T	C	1	ISSFT-021, IF1SW-F4			-	Cluster_6_fumigaclavine
Chr2	4781532	SNV	C	T	1	ISSFT-021, IF1SW-F4	Afu2g17980:c.157-94G>A		-	Cluster_6_fumigaclavine
Chr2	4781542	SNV	A	G	1	ISSFT-021, IF1SW-F4	Afu2g17980:c.157-104T>C		-	Cluster_6_fumigaclavine
Chr2	4781624	SNV	G	A	1	CEA10	Afu2g17980:c.156+106C>T		-	Cluster_6_fumigaclavine
Chr2	4783007	SNV	G	C	1	ISSFT-021, IF1SW-F4	Afu2g17990:c.773C>G	Afu2g17990:p.Pro258Arg	Yes	Cluster_6_fumigaclavine
Chr2	4783046	Deletion	A	-	1	IF1SW-F4	Afu2g17990:c.734delT	Afu2g17990:p.Phe245fs	Yes	Cluster_6_fumigaclavine
Chr2	4786100	SNV	C	A	1	ISSFT-021, IF1SW-F4	Afu2g18010:c.87C>A	Afu2g18010:p.Phe29Leu	Yes	Cluster_6_fumigaclavine
Chr2	4786625	SNV	T	C	1	IF1SW-F4	Afu2g18010:c.457+16T>C		-	Cluster_6_fumigaclavine
Chr2	4787958	SNV	G	A	1	ISSFT-021, IF1SW-F4			-	Cluster_6_fumigaclavine
Chr2	4793630	SNV	C	G	1	ISSFT-021, IF1SW-F4	Afu2g18040:c.1332G>C		No	Cluster_6_fumigaclavine
Chr2	4793659	SNV	A	C	1	ISSFT-021, IF1SW-F4	Afu2g18040:c.1303T>G	Afu2g18040:p.Ser435Ala	Yes	Cluster_6_fumigaclavine
Chr2	4793989	SNV	T	C	1	ISSFT-021, IF1SW-F4	Afu2g18040:c.1132A>G	Afu2g18040:p.Thr378Ala	Yes	Cluster_6_fumigaclavine
Chr2	4797640	SNV	A	G	1	ISSFT-021	Afu2g18050:c.1785A>G		No	Cluster_6_fumigaclavine
Chr2	4797705	SNV	A	C	1	ISSFT-021, IF1SW-F4	Afu2g18050:c.1850A>C	Afu2g18050:p.Tyr617Ser	Yes	Cluster_6_fumigaclavine
Chr2	4798785	SNV	T	C	1	ISSFT-021, IF1SW-F4	Afu2g18060:c.715T>C	Afu2g18060:p.Phe239Leu	Yes	Cluster_6_fumigaclavine
Chr3	348181	SNV	G	A	1	CEA10, ISSFT-021, IF1SW-F4	Afu3g01400:c.-19G>A		-	Cluster_7_PKS
Chr3	348219	SNV	T	C	1	CEA10, ISSFT-021, IF1SW-F4	Afu3g01400:c.20T>C	Afu3g01400:p.Leu7Pro	Yes	Cluster_7_PKS
Chr3	348361	SNV	T	C	1	CEA10, ISSFT-021, IF1SW-F4	Afu3g01400:c.162T>C		No	Cluster_7_PKS
Chr3	348772	SNV	T	C	1	CEA10, ISSFT-021, IF1SW-F4	Afu3g01400:c.573T>C		No	Cluster_7_PKS
Chr3	348835	SNV	T	C	1	CEA10, ISSFT-021, IF1SW-F4	Afu3g01400:c.636T>C		No	Cluster_7_PKS
Chr3	350598	SNV	G	A	1	CEA10, ISSFT-021, IF1SW-F4	Afu3g01400:c.2320G>A	Afu3g01400:p.Gly774Ser	Yes	Cluster_7_PKS

Chr3	351027	SNV	T	C	1	IF1SW-F4	Afu3g01400:c.2749T>C		No	Cluster_7_PKS
Chr3	351344	SNV	C	T	1	IF1SW-F4	Afu3g01400:c.3066C>T		No	Cluster_7_PKS
Chr3	351416	SNV	T	C	1	IF1SW-F4	Afu3g01400:c.3138T>C		No	Cluster_7_PKS
Chr3	351428	SNV	T	C	1	CEA10, ISSFT-021, IF1SW-F4	Afu3g01400:c.3150T>C		No	Cluster_7_PKS
Chr3	351917	SNV	T	C	1	IF1SW-F4	Afu3g01400:c.3639T>C		No	Cluster_7_PKS
Chr3	352052^352053	Insertion	-	C	1	CEA10, ISSFT-021	Afu3g01400:c.3759+15_3759+16insC		-	Cluster_7_PKS
Chr3	352057	SNV	A	C	1	IF1SW-F4	Afu3g01400:c.3759+20A>C		-	Cluster_7_PKS
Chr3	352150	SNV	T	C	1	IF1SW-F4	Afu3g01400:c.3804T>C		No	Cluster_7_PKS
Chr3	352189	SNV	T	C	1	CEA10, ISSFT-021, IF1SW-F4	Afu3g01400:c.3843T>C		No	Cluster_7_PKS
Chr3	352432	SNV	T	C	1	CEA10, ISSFT-021, IF1SW-F4	Afu3g01400:c.4086T>C		No	Cluster_7_PKS
Chr3	352656	SNV	A	G	1	IF1SW-F4			-	Cluster_7_PKS
Chr3	352844	Deletion	A	-	1	IF1SW-F4			-	Cluster_7_PKS
Chr3	352898	SNV	T	G	1	CEA10, ISSFT-021, IF1SW-F4	Afu3g01410:c.7641A>C		No	Cluster_7_PKS
Chr3	353000	SNV	T	C	1	CEA10, ISSFT-021, IF1SW-F4	Afu3g01410:c.7539A>G		No	Cluster_7_PKS
Chr3	353163	SNV	T	A	1	IF1SW-F4	Afu3g01410:c.7376A>T	Afu3g01410:p.Glu2459Val	Yes	Cluster_7_PKS
Chr3	353288	SNV	A	G	1	IF1SW-F4	Afu3g01410:c.7251T>C		No	Cluster_7_PKS
Chr3	353388	SNV	T	G	1	IF1SW-F4	Afu3g01410:c.7151A>C	Afu3g01410:p.Glu2384Ala	Yes	Cluster_7_PKS
Chr3	353394	SNV	G	T	1	IF1SW-F4	Afu3g01410:c.7145C>A	Afu3g01410:p.Ala2382Asp	Yes	Cluster_7_PKS
Chr3	353461	SNV	T	C	1	IF1SW-F4	Afu3g01410:c.7078A>G	Afu3g01410:p.Ser2360Gly	Yes	Cluster_7_PKS
Chr3	353582	SNV	G	C	1	IF1SW-F4	Afu3g01410:c.6957C>G		No	Cluster_7_PKS
Chr3	353706	SNV	A	G	1	IF1SW-F4	Afu3g01410:c.6861+28T>C		-	Cluster_7_PKS
Chr3	354516	SNV	C	T	1	IF1SW-F4	Afu3g01410:c.6125G>A	Afu3g01410:p.Gly2042Glu	Yes	Cluster_7_PKS
Chr3	354604	SNV	A	T	1	IF1SW-F4	Afu3g01410:c.6037T>A	Afu3g01410:p.Leu2013Ile	Yes	Cluster_7_PKS
Chr3	354622	SNV	T	G	1	CEA10, ISSFT-021, IF1SW-F4	Afu3g01410:c.6019A>C	Afu3g01410:p.Asn2007His	Yes	Cluster_7_PKS
Chr3	354699	SNV	C	T	1	CEA10, ISSFT-021	Afu3g01410:c.5942G>A	Afu3g01410:p.Arg1981Gln	Yes	Cluster_7_PKS
Chr3	354707	SNV	G	T	1	IF1SW-F4	Afu3g01410:c.5934C>A	Afu3g01410:p.Asn1978Lys	Yes	Cluster_7_PKS
Chr3	354806	SNV	C	T	1	IF1SW-F4	Afu3g01410:c.5835G>A		No	Cluster_7_PKS
Chr3	354990	SNV	T	C	1	IF1SW-F4	Afu3g01410:c.5651A>G	Afu3g01410:p.Tyr1884Cys	Yes	Cluster_7_PKS
Chr3	355011	SNV	T	C	1	IF1SW-F4	Afu3g01410:c.5630A>G	Afu3g01410:p.Asp1877Gly	Yes	Cluster_7_PKS
Chr3	355121	SNV	G	A	1	CEA10, ISSFT-021, IF1SW-F4	Afu3g01410:c.5520C>T		No	Cluster_7_PKS
Chr3	355280	SNV	A	T	1	IF1SW-F4	Afu3g01410:c.5361T>A		No	Cluster_7_PKS
Chr3	355296	SNV	T	C	1	IF1SW-F4	Afu3g01410:c.5345A>G	Afu3g01410:p.His1782Arg	Yes	Cluster_7_PKS
Chr3	355355	SNV	G	T	1	IF1SW-F4	Afu3g01410:c.5286C>A		No	Cluster_7_PKS
Chr3	355712	SNV	C	G	1	IF1SW-F4	Afu3g01410:c.4929G>C	Afu3g01410:p.Met1643Ile	Yes	Cluster_7_PKS
Chr3	355822	SNV	G	A	1	IF1SW-F4	Afu3g01410:c.4819C>T	Afu3g01410:p.Pro1607Ser	Yes	Cluster_7_PKS
Chr3	355868	SNV	G	C	1	CEA10, ISSFT-021	Afu3g01410:c.4773C>G	Afu3g01410:p.Asp1591Glu	Yes	Cluster_7_PKS
Chr3	355876	SNV	T	C	1	IF1SW-F4	Afu3g01410:c.4765A>G	Afu3g01410:p.Arg1589Gly	Yes	Cluster_7_PKS
Chr3	356048	SNV	C	T	1	CEA10, ISSFT-021	Afu3g01410:c.4593G>A		No	Cluster_7_PKS
Chr3	356137	SNV	T	C	1	CEA10, ISSFT-021	Afu3g01410:c.4504A>G	Afu3g01410:p.Arg1502Gly	Yes	Cluster_7_PKS
Chr3	356176	SNV	C	T	1	CEA10, ISSFT-021	Afu3g01410:c.4465G>A	Afu3g01410:p.Glu1489Lys	Yes	Cluster_7_PKS
Chr3	356682	SNV	G	A	1	CEA10	Afu3g01410:c.3959C>T	Afu3g01410:p.Ser1320Phe	Yes	Cluster_7_PKS
Chr3	356723	SNV	A	C	1	IF1SW-F4	Afu3g01410:c.3918T>G		No	Cluster_7_PKS
Chr3	356877	SNV	C	T	1	CEA10, ISSFT-021, IF1SW-F4	Afu3g01410:c.3764G>A	Afu3g01410:p.Arg1255Lys	Yes	Cluster_7_PKS
Chr3	357219	SNV	G	A	1	IF1SW-F4	Afu3g01410:c.3422C>T	Afu3g01410:p.Thr1141Ile	Yes	Cluster_7_PKS
Chr3	357226	SNV	A	G	1	CEA10	Afu3g01410:c.3415T>C	Afu3g01410:p.Tyr1139His	Yes	Cluster_7_PKS
Chr3	357252	SNV	G	C	1	IF1SW-F4	Afu3g01410:c.3389C>G	Afu3g01410:p.Ala1130Gly	Yes	Cluster_7_PKS
Chr3	357428	SNV	A	G	1	CEA10, ISSFT-021	Afu3g01410:c.3213T>C		No	Cluster_7_PKS
Chr3	357566	SNV	G	A	1	IF1SW-F4	Afu3g01410:c.3075C>T		No	Cluster_7_PKS
Chr3	357583	SNV	T	C	1	CEA10, ISSFT-021, IF1SW-F4	Afu3g01410:c.3058A>G	Afu3g01410:p.Met1020Val	Yes	Cluster_7_PKS
Chr3	357619	SNV	G	A	1	IF1SW-F4	Afu3g01410:c.3022C>T		No	Cluster_7_PKS
Chr3	357898	SNV	T	C	1	CEA10, ISSFT-021, IF1SW-F4	Afu3g01410:c.2743A>G	Afu3g01410:p.Asn915Asp	Yes	Cluster_7_PKS
Chr3	357928	SNV	G	A	1	CEA10, ISSFT-021	Afu3g01410:c.2713C>T	Afu3g01410:p.Arg905Cys	Yes	Cluster_7_PKS
Chr3	358121	SNV	A	G	1	IF1SW-F4	Afu3g01410:c.2520T>C		No	Cluster_7_PKS
Chr3	358147	SNV	C	T	1	IF1SW-F4	Afu3g01410:c.2494G>A	Afu3g01410:p.Glu832Lys	Yes	Cluster_7_PKS
Chr3	358208	SNV	C	A	1	IF1SW-F4	Afu3g01410:c.2433G>T		No	Cluster_7_PKS
Chr3	358313	SNV	T	C	1	IF1SW-F4	Afu3g01410:c.2328A>G		No	Cluster_7_PKS
Chr3	358370	SNV	T	A	1	CEA10	Afu3g01410:c.2271A>T		No	Cluster_7_PKS
Chr3	358460..358463	MNV	TCCT	ACCG	4	IF1SW-F4	fu3g01410:c.2178_2181delAGGAinsCGG		No	Cluster_7_PKS
Chr3	358514	SNV	T	A	1	IF1SW-F4	Afu3g01410:c.2127A>T		No	Cluster_7_PKS

Chr3	358688	SNV	A	G	1	IF1SW-F4	Afu3g01410:c.1953T>C		No	Cluster_7_PKS
Chr3	358724	SNV	T	C	1	IF1SW-F4	Afu3g01410:c.1917A>G		No	Cluster_7_PKS
Chr3	358763..358765	Deletion	TCT	-	3	IF1SW-F4	Afu3g01410:c.1904-28_1904-26delAGA		-	Cluster_7_PKS
Chr3	358783..358786	MNV	ACCC	GCCT	4	IF1SW-F4	g01410:c.1903+31_1903+34delGGGTinsA		-	Cluster_7_PKS
Chr3	358810	SNV	C	T	1	CEA10, ISSFT-021	Afu3g01410:c.1903+7G>A		-	Cluster_7_PKS
Chr3	359029	SNV	C	T	1	CEA10, ISSFT-021, IF1SW-F4	Afu3g01410:c.1691G>A	Afu3g01410:p.Ser564Asn	Yes	Cluster_7_PKS
Chr3	359296	SNV	G	A	1	IF1SW-F4	Afu3g01410:c.1424C>T	Afu3g01410:p.Pro475Leu	Yes	Cluster_7_PKS
Chr3	359465	SNV	A	G	1	CEA10, ISSFT-021, IF1SW-F4	Afu3g01410:c.1284+23T>C		-	Cluster_7_PKS
Chr3	359508	SNV	G	A	1	IF1SW-F4	Afu3g01410:c.1264C>T		No	Cluster_7_PKS
Chr3	359607	SNV	T	C	1	IF1SW-F4	Afu3g01410:c.1165A>G	Afu3g01410:p.Thr389Ala	Yes	Cluster_7_PKS
Chr3	359803	SNV	G	C	1	IF1SW-F4	Afu3g01410:c.969C>G		No	Cluster_7_PKS
Chr3	359915	SNV	T	G	1	IF1SW-F4	Afu3g01410:c.857A>C	Afu3g01410:p.Lys286Thr	Yes	Cluster_7_PKS
Chr3	360149	SNV	C	T	1	IF1SW-F4	Afu3g01410:c.672G>A		No	Cluster_7_PKS
Chr3	360242	SNV	C	T	1	CEA10, ISSFT-021	Afu3g01410:c.579G>A		No	Cluster_7_PKS
Chr3	360492	SNV	G	T	1	CEA10, ISSFT-021	Afu3g01410:c.379C>A		No	Cluster_7_PKS
Chr3	360556	SNV	G	A	1	CEA10, ISSFT-021, IF1SW-F4	Afu3g01410:c.360+22C>T		-	Cluster_7_PKS
Chr3	360602	SNV	G	A	1	CEA10	Afu3g01410:c.336C>T		No	Cluster_7_PKS
Chr3	360878	SNV	C	T	1	IF1SW-F4	Afu3g01410:c.108G>A	Afu3g01410:p.Met36Ile	Yes	Cluster_7_PKS
Chr3	361176	SNV	A	G	1	CEA10, ISSFT-021	Afu3g01420:c.*100T>C		-	Cluster_7_PKS
Chr3	361332	SNV	C	T	1	CEA10, ISSFT-021	Afu3g01420:c.622G>A	Afu3g01420:p.Ala208Thr	Yes	Cluster_7_PKS
Chr3	361382	SNV	G	C	1	CEA10, ISSFT-021, IF1SW-F4	Afu3g01420:c.572C>G	Afu3g01420:p.Ser191Trp	Yes	Cluster_7_PKS
Chr3	361440	SNV	G	A	1	IF1SW-F4	Afu3g01420:c.514C>T	Afu3g01420:p.Pro172Ser	Yes	Cluster_7_PKS
Chr3	361496	SNV	G	A	1	IF1SW-F4	Afu3g01420:c.458C>T	Afu3g01420:p.Ser153Leu	Yes	Cluster_7_PKS
Chr3	361508..361509	MNV	AT	GG	2	IF1SW-F4	Afu3g01420:c.445_446delATinsCC	Afu3g01420:p.Ile149Pro	Yes	Cluster_7_PKS
Chr3	361609	SNV	C	G	1	IF1SW-F4	Afu3g01420:c.345G>C		No	Cluster_7_PKS
Chr3	361925	SNV	T	C	1	CEA10, ISSFT-021, IF1SW-F4	Afu3g01420:c.67+9A>G		-	Cluster_7_PKS
Chr3	362031	SNV	A	G	1	IF1SW-F4	Afu3g01420:c.21T>C		No	Cluster_7_PKS
Chr3	362068	SNV	C	T	1	CEA10, ISSFT-021			-	Cluster_7_PKS
Chr3	362107	SNV	T	A	1	CEA10, ISSFT-021, IF1SW-F4			-	Cluster_7_PKS
Chr3	362198	SNV	T	C	1	IF1SW-F4			-	Cluster_7_PKS
Chr3	362355	SNV	C	T	1	IF1SW-F4			-	Cluster_7_PKS
Chr3	362380	SNV	A	G	1	CEA10, ISSFT-021			-	Cluster_7_PKS
Chr3	362601	Deletion	A	-	1	IF1SW-F4			-	Cluster_7_PKS
Chr3	362624	SNV	G	A	1	IF1SW-F4			-	Cluster_7_PKS
Chr3	362650..362653	Deletion	ATGG	-	4	IF1SW-F4			-	Cluster_7_PKS
Chr3	363080	SNV	C	T	1	IF1SW-F4	Afu3g01430:c.222C>T		No	Cluster_7_PKS
Chr3	363200	SNV	G	C	1	CEA10, ISSFT-021	Afu3g01430:c.342G>C		No	Cluster_7_PKS
Chr3	363347	SNV	A	T	1	IF1SW-F4	Afu3g01430:c.489A>T		No	Cluster_7_PKS
Chr3	363588^363589	Insertion	-	G	1	IF1SW-F4	Afu3g01430:c.*190_*191insG		-	Cluster_7_PKS
Chr3	363704	SNV	T	C	1	IF1SW-F4			-	Cluster_7_PKS
Chr3	363945	SNV	A	C	1	CEA10, ISSFT-021, IF1SW-F4	Afu3g01440:c.-693A>C		-	Cluster_7_PKS
Chr3	363984	SNV	G	A	1	IF1SW-F4	Afu3g01440:c.-654G>A		-	Cluster_7_PKS
Chr3	364086	SNV	A	G	1	IF1SW-F4	Afu3g01440:c.-552A>G		-	Cluster_7_PKS
Chr3	364189	SNV	T	C	1	IF1SW-F4	Afu3g01440:c.-449T>C		-	Cluster_7_PKS
Chr3	364249^364250	Insertion	-	T	1	IF1SW-F4	Afu3g01440:c.-389_-388insT		-	Cluster_7_PKS
Chr3	364286	SNV	A	T	1	IF1SW-F4	Afu3g01440:c.-352A>T		-	Cluster_7_PKS
Chr3	364361	SNV	C	T	1	CEA10, ISSFT-021, IF1SW-F4	Afu3g01440:c.-277C>T		-	Cluster_7_PKS
Chr3	364546	SNV	G	T	1	CEA10, ISSFT-021	Afu3g01440:c.-92G>T		-	Cluster_7_PKS
Chr3	364646	SNV	G	T	1	IF1SW-F4	Afu3g01440:c.9G>T	Afu3g01440:p.Glu3Asp	Yes	Cluster_7_PKS
Chr3	364658	SNV	T	A	1	IF1SW-F4	Afu3g01440:c.21T>A	Afu3g01440:p.Asp7Glu	Yes	Cluster_7_PKS
Chr3	364755	SNV	A	G	1	IF1SW-F4	Afu3g01440:c.118A>G	Afu3g01440:p.Asn40Asp	Yes	Cluster_7_PKS
Chr3	365912	SNV	G	A	1	IF1SW-F4	Afu3g01440:c.1275G>A		No	Cluster_7_PKS
Chr3	366251	SNV	C	T	1	CEA10, ISSFT-021	Afu3g01440:c.1614C>T		No	Cluster_7_PKS
Chr3	366404	SNV	G	T	1	IF1SW-F4	Afu3g01440:c.1767G>T		No	Cluster_7_PKS
Chr3	366421	SNV	T	C	1	CEA10, ISSFT-021, IF1SW-F4	Afu3g01440:c.1784T>C	Afu3g01440:p.Phe595Ser	Yes	Cluster_7_PKS
Chr3	366464..366468	MNV	AATTT	GATTC	5	IF1SW-F4	fu3g01440:c.1827_1831delAATTTinsGAT		No	Cluster_7_PKS
Chr3	366767	SNV	T	C	1	IF1SW-F4	Afu3g01440:c.2130T>C		No	Cluster_7_PKS
Chr3	366885	SNV	T	C	1	IF1SW-F4	Afu3g01440:c.2248T>C		No	Cluster_7_PKS
Chr3	366920	SNV	T	G	1	IF1SW-F4	Afu3g01440:c.2283T>G		No	Cluster_7_PKS

Chr3	367244	SNV	C	T	1	IF1SW-F4	Afu3g01440:c.*69C>T		-	Cluster_7_PKS
Chr3	367303	SNV	T	C	1	IF1SW-F4	Afu3g01440:c.*128T>C		-	Cluster_7_PKS
Chr3	367313	SNV	C	T	1	IF1SW-F4	Afu3g01440:c.*138C>T		-	Cluster_7_PKS
Chr3	367344	SNV	A	G	1	CEA10, ISSFT-021, IF1SW-F4	Afu3g01440:c.*169A>G		-	Cluster_7_PKS
Chr3	367376	SNV	G	T	1	IF1SW-F4			-	Cluster_7_PKS
Chr3	367560	SNV	C	T	1	CEA10, ISSFT-021, IF1SW-F4			-	Cluster_7_PKS
Chr3	367560..367564	MNV	CCTCA	TCTCG	5	IF1SW-F4			-	Cluster_7_PKS
Chr3	367575	SNV	A	G	1	CEA10, ISSFT-021, IF1SW-F4			-	Cluster_7_PKS
Chr3	367686	SNV	G	A	1	IF1SW-F4			-	Cluster_7_PKS
Chr3	367735	SNV	G	A	1	IF1SW-F4			-	Cluster_7_PKS
Chr3	367799	SNV	A	G	1	IF1SW-F4			-	Cluster_7_PKS
Chr3	367878	SNV	C	A	1	CEA10, ISSFT-021, IF1SW-F4			-	Cluster_7_PKS
Chr3	367884	SNV	G	A	1	CEA10, ISSFT-021			-	Cluster_7_PKS
Chr3	367891	SNV	G	A	1	IF1SW-F4			-	Cluster_7_PKS
Chr3	368013	SNV	G	A	1	IF1SW-F4			-	Cluster_7_PKS
Chr3	368025	SNV	C	G	1	IF1SW-F4			-	Cluster_7_PKS
Chr3	368051	SNV	T	G	1	CEA10, ISSFT-021			-	Cluster_7_PKS
Chr3	368112	SNV	G	A	1	CEA10, ISSFT-021			-	Cluster_7_PKS
Chr3	368195	SNV	T	A	1	CEA10, ISSFT-021			-	Cluster_7_PKS
Chr3	368276	SNV	A	G	1	CEA10, ISSFT-021, IF1SW-F4			-	Cluster_7_PKS
Chr3	368359	SNV	A	G	1	IF1SW-F4			-	Cluster_7_PKS
Chr3	368364	SNV	T	A	1	IF1SW-F4			-	Cluster_7_PKS
Chr3	368407	SNV	A	C	1	IF1SW-F4			-	Cluster_7_PKS
Chr3	368449	SNV	C	A	1	CEA10, ISSFT-021			-	Cluster_7_PKS
Chr3	368456	SNV	G	A	1	CEA10, ISSFT-021			-	Cluster_7_PKS
Chr3	368462	SNV	A	G	1	IF1SW-F4			-	Cluster_7_PKS
Chr3	368644	SNV	A	C	1	IF1SW-F4			-	Cluster_7_PKS
Chr3	368664	SNV	T	C	1	IF1SW-F4	Afu3g01450:c.*87A>G		-	Cluster_7_PKS
Chr3	368992	SNV	A	G	1	CEA10, ISSFT-021, IF1SW-F4	Afu3g01450:c.987T>C		No	Cluster_7_PKS
Chr3	369128	SNV	C	T	1	CEA10, ISSFT-021	Afu3g01450:c.851G>A	Afu3g01450:p.Arg284Lys	Yes	Cluster_7_PKS
Chr3	369219	SNV	A	G	1	CEA10, ISSFT-021, IF1SW-F4	Afu3g01450:c.760T>C	Afu3g01450:p.Ser254Pro	Yes	Cluster_7_PKS
Chr3	369304	SNV	C	G	1	IF1SW-F4	Afu3g01450:c.675G>C	Afu3g01450:p.Gln225His	Yes	Cluster_7_PKS
Chr3	369309	SNV	A	G	1	CEA10	Afu3g01450:c.670T>C	Afu3g01450:p.Phe224Leu	Yes	Cluster_7_PKS
Chr3	369310..369313	MNV	GATT	AATC	4	IF1SW-F4	Afu3g01450:c.666_669delAATCinsGATT		No	Cluster_7_PKS
Chr3	369400	SNV	G	T	1	IF1SW-F4	Afu3g01450:c.579C>A		No	Cluster_7_PKS
Chr3	369532	SNV	T	C	1	IF1SW-F4	Afu3g01450:c.507A>G		No	Cluster_7_PKS
Chr3	369544	SNV	T	C	1	IF1SW-F4	Afu3g01450:c.495A>G		No	Cluster_7_PKS
Chr3	369661	SNV	A	G	1	IF1SW-F4	Afu3g01450:c.378T>C		No	Cluster_7_PKS
Chr3	369882	SNV	G	A	1	CEA10, ISSFT-021	Afu3g01450:c.157C>T		No	Cluster_7_PKS
Chr3	370052	SNV	G	A	1	CEA10, ISSFT-021, IF1SW-F4	Afu3g01450:c.-14C>T		-	Cluster_7_PKS
Chr3	370068	SNV	G	A	1	IF1SW-F4	Afu3g01450:c.-30C>T		-	Cluster_7_PKS
Chr3	370097	SNV	T	C	1	CEA10, ISSFT-021, IF1SW-F4	Afu3g01450:c.-59A>G		-	Cluster_7_PKS
Chr3	370173	SNV	C	T	1	CEA10, ISSFT-021, IF1SW-F4			-	Cluster_7_PKS
Chr3	370263	SNV	G	A	1	CEA10, ISSFT-021, IF1SW-F4			-	Cluster_7_PKS
Chr3	370263..370267	MNV	GATTC	AATAT	5	IF1SW-F4			-	Cluster_7_PKS
Chr3	370274	SNV	A	C	1	IF1SW-F4			-	Cluster_7_PKS
Chr3	370282	SNV	C	T	1	IF1SW-F4			-	Cluster_7_PKS
Chr3	370332	SNV	C	T	1	IF1SW-F4			-	Cluster_7_PKS
Chr3	370566	SNV	G	C	1	IF1SW-F4	Afu3g01460:c.1126C>G	Afu3g01460:p.His376Asp	Yes	Cluster_7_PKS
Chr3	370606	SNV	T	A	1	IF1SW-F4	Afu3g01460:c.1086A>T		No	Cluster_7_PKS
Chr3	370774	SNV	A	G	1	IF1SW-F4	Afu3g01460:c.918T>C		No	Cluster_7_PKS
Chr3	370820	SNV	A	G	1	IF1SW-F4	Afu3g01460:c.872T>C	Afu3g01460:p.Leu291Ser	Yes	Cluster_7_PKS
Chr3	370985	SNV	T	C	1	CEA10, ISSFT-021	Afu3g01460:c.788A>G	Afu3g01460:p.Lys263Arg	Yes	Cluster_7_PKS
Chr3	371316	SNV	G	T	1	CEA10, ISSFT-021, IF1SW-F4	Afu3g01460:c.457C>A	Afu3g01460:p.His153Asn	Yes	Cluster_7_PKS
Chr3	371474	SNV	T	C	1	CEA10, ISSFT-021	Afu3g01460:c.299A>G	Afu3g01460:p.Lys100Arg	Yes	Cluster_7_PKS
Chr3	371739	SNV	T	C	1	CEA10, ISSFT-021	Afu3g01460:c.34A>G	Afu3g01460:p.Ile12Val	Yes	Cluster_7_PKS
Chr3	371816	SNV	C	T	1	IF1SW-F4			-	Cluster_7_PKS
Chr3	371884	SNV	C	A	1	CEA10, ISSFT-021, IF1SW-F4			-	Cluster_7_PKS
Chr3	372037	SNV	G	A	1	IF1SW-F4			-	Cluster_7_PKS

Chr3	372048	SNV	A	G	1	IF1SW-F4			-	Cluster_7_PKS
Chr3	372124	SNV	T	G	1	IF1SW-F4			-	Cluster_7_PKS
Chr3	372166	SNV	A	G	1	IF1SW-F4			-	Cluster_7_PKS
Chr3	372414	SNV	C	G	1	CEA10, ISSFT-021, IF1SW-F4			-	Cluster_7_PKS
Chr3	372608	SNV	T	G	1	IF1SW-F4			-	Cluster_7_PKS
Chr3	372797	SNV	A	G	1	IF1SW-F4	Afu3g01470:c.112A>G	Afu3g01470:p.Thr38Ala	Yes	Cluster_7_PKS
Chr3	373008	SNV	C	T	1	IF1SW-F4	Afu3g01470:c.299+24C>T		-	Cluster_7_PKS
Chr3	373049	SNV	C	T	1	CEA10, ISSFT-021	Afu3g01470:c.308C>T	Afu3g01470:p.Ser103Leu	Yes	Cluster_7_PKS
Chr3	373148..373150	MNV	CGG	TGA	3	IF1SW-F4	Afu3g01470:c.357_359delCGGinsTGA	Afu3g01470:p.Gly120Asp	Yes	Cluster_7_PKS
Chr3	373300	SNV	G	A	1	CEA10, ISSFT-021, IF1SW-F4	Afu3g01470:c.454G>A	Afu3g01470:p.Val152Met	Yes	Cluster_7_PKS
Chr3	373349	SNV	T	C	1	CEA10	Afu3g01470:c.487+16T>C		-	Cluster_7_PKS
Chr3	373443	SNV	T	C	1	CEA10, ISSFT-021, IF1SW-F4	Afu3g01470:c.552T>C		No	Cluster_7_PKS
Chr3	373661	SNV	C	A	1	CEA10, ISSFT-021, IF1SW-F4	Afu3g01470:c.719C>A	Afu3g01470:p.Ala240Asp	Yes	Cluster_7_PKS
Chr3	373804..373809	MNV	TAATAC	ATATAT	6	IF1SW-F4			-	Cluster_7_PKS
Chr3	373809	SNV	C	T	1	CEA10, ISSFT-021, IF1SW-F4			-	Cluster_7_PKS
Chr3	373908	SNV	G	A	1	CEA10, ISSFT-021, IF1SW-F4			-	Cluster_7_PKS
Chr3	374139	SNV	T	C	1	CEA10, ISSFT-021, IF1SW-F4	Afu3g01480:c.1612A>G	Afu3g01480:p.Ile538Val	Yes	Cluster_7_PKS
Chr3	374276	SNV	C	T	1	CEA10, ISSFT-021, IF1SW-F4	Afu3g01480:c.1475G>A	Afu3g01480:p.Gly492Asp	Yes	Cluster_7_PKS
Chr3	374349	SNV	A	G	1	CEA10, ISSFT-021, IF1SW-F4	Afu3g01480:c.1402T>C	Afu3g01480:p.Tyr468His	Yes	Cluster_7_PKS
Chr3	374409..374410	MNV	TT	CC	2	CEA10, ISSFT-021, IF1SW-F4	Afu3g01480:c.1341_1342delAAinsGG	Afu3g01480:p.Ser448Gly	Yes	Cluster_7_PKS
Chr3	374588	SNV	C	T	1	CEA10, ISSFT-021, IF1SW-F4	Afu3g01480:c.1163G>A	Afu3g01480:p.Arg388Lys	Yes	Cluster_7_PKS
Chr3	374794	SNV	G	C	1	ISSFT-021	Afu3g01480:c.957C>G	Afu3g01480:p.Phe319Leu	Yes	Cluster_7_PKS
Chr3	374857	SNV	A	G	1	CEA10, ISSFT-021, IF1SW-F4	Afu3g01480:c.894T>C		No	Cluster_7_PKS
Chr3	375287	SNV	T	G	1	CEA10, ISSFT-021, IF1SW-F4	Afu3g01480:c.513+20A>C		-	Cluster_7_PKS
Chr3	375456	SNV	T	C	1	CEA10, ISSFT-021, IF1SW-F4	Afu3g01480:c.364A>G	Afu3g01480:p.Ile122Val	Yes	Cluster_7_PKS
Chr3	902923	SNV	T	C	1	CEA10, ISSFT-021, IF1SW-F4	Afu3g03390:c.458A>G	Afu3g03390:p.Lys153Arg	Yes	Cluster_8_fusarine_C
Chr3	903424	SNV	A	C	1	CEA10, ISSFT-021, IF1SW-F4	Afu3g03390:c.17T>G	Afu3g03390:p.Phe6Cys	Yes	Cluster_8_fusarine_C
Chr3	903646	SNV	T	A	1	CEA10, ISSFT-021, IF1SW-F4			-	Cluster_8_fusarine_C
Chr3	907891	SNV	G	A	1	CEA10, ISSFT-021, IF1SW-F4			-	Cluster_8_fusarine_C
Chr3	908129	SNV	G	A	1	CEA10			-	Cluster_8_fusarine_C
Chr3	908676	SNV	A	G	1	CEA10, ISSFT-021, IF1SW-F4	Afu3g03420:c.454A>G	Afu3g03420:p.Ile152Val	Yes	Cluster_8_fusarine_C
Chr3	908790	SNV	A	G	1	CEA10, ISSFT-021, IF1SW-F4	Afu3g03420:c.568A>G	Afu3g03420:p.Ile190Val	Yes	Cluster_8_fusarine_C
Chr3	909217	SNV	A	G	1	CEA10	Afu3g03420:c.995A>G	Afu3g03420:p.Tyr332Cys	Yes	Cluster_8_fusarine_C
Chr3	910578	SNV	T	G	1	CEA10, ISSFT-021, IF1SW-F4	Afu3g03420:c.2356T>G	Afu3g03420:p.Cys786Gly	Yes	Cluster_8_fusarine_C
Chr3	912458	SNV	A	G	1	CEA10, ISSFT-021, IF1SW-F4	Afu3g03420:c.4236A>G		No	Cluster_8_fusarine_C
Chr3	914352	SNV	T	G	1	CEA10, ISSFT-021, IF1SW-F4	Afu3g03420:c.6130T>G	Afu3g03420:p.Cys2044Gly	Yes	Cluster_8_fusarine_C
Chr3	916387	SNV	C	T	1	CEA10, ISSFT-021, IF1SW-F4	Afu3g03430:c.2163G>A		No	Cluster_8_fusarine_C
Chr3	917512	SNV	G	A	1	CEA10, ISSFT-021, IF1SW-F4	Afu3g03430:c.1038C>T		No	Cluster_8_fusarine_C
Chr3	917647	SNV	G	A	1	CEA10, ISSFT-021, IF1SW-F4	Afu3g03430:c.934+16C>T		-	Cluster_8_fusarine_C
Chr3	917733	SNV	A	C	1	CEA10, ISSFT-021, IF1SW-F4	Afu3g03430:c.864T>G	Afu3g03430:p.Asp288Glu	Yes	Cluster_8_fusarine_C
Chr3	918377	SNV	G	A	1	CEA10	Afu3g03430:c.220C>T	Afu3g03430:p.His74Tyr	Yes	Cluster_8_fusarine_C
Chr3	918646..918649	MNV	CTGT	TTGC	4	CEA10, ISSFT-021, IF1SW-F4			-	Cluster_8_fusarine_C
Chr3	918865	SNV	A	G	1	CEA10, ISSFT-021, IF1SW-F4			-	Cluster_8_fusarine_C
Chr3	919011	SNV	G	C	1	ISSFT-021			-	Cluster_8_fusarine_C
Chr3	919181	SNV	T	A	1	IF1SW-F4			-	Cluster_8_fusarine_C
Chr3	919287	SNV	G	A	1	CEA10, ISSFT-021, IF1SW-F4	Afu3g03440:c.-87G>A		-	Cluster_8_fusarine_C
Chr3	919603	SNV	T	C	1	CEA10, ISSFT-021, IF1SW-F4	Afu3g03440:c.230T>C	Afu3g03440:p.Ile77Thr	Yes	Cluster_8_fusarine_C
Chr3	921447	SNV	C	T	1	CEA10, ISSFT-021, IF1SW-F4	Afu3g03440:c.*121C>T		-	Cluster_8_fusarine_C
Chr3	921463	SNV	G	A	1	CEA10, ISSFT-021, IF1SW-F4	Afu3g03440:c.*137G>A		-	Cluster_8_fusarine_C
Chr3	921481	SNV	A	C	1	CEA10, ISSFT-021, IF1SW-F4	Afu3g03440:c.*155A>C		-	Cluster_8_fusarine_C
Chr3	921696	SNV	C	T	1	CEA10, ISSFT-021, IF1SW-F4			-	Cluster_8_fusarine_C
Chr3	921726	SNV	T	G	1	CEA10, ISSFT-021, IF1SW-F4			-	Cluster_8_fusarine_C
Chr3	3423839	SNV	G	T	1	CEA10			-	Cluster_9_hexadehydroastechrome
Chr3	3424055	SNV	T	C	1	ISSFT-021, IF1SW-F4	Afu3g12890:c.1047A>G		No	Cluster_9_hexadehydroastechrome
Chr3	3424601	SNV	A	G	1	CEA10, ISSFT-021, IF1SW-F4	Afu3g12890:c.501T>C		No	Cluster_9_hexadehydroastechrome
Chr3	3425485^3425486	Insertion	-	TGTTAATTT[illegible]	16	CEA10	[illegible]g12890:c.-333_-332insTTGGAAATTAACA[illegible]		-	Cluster_9_hexadehydroastechrome
Chr3	3425589	SNV	C	G	1	CEA10, ISSFT-021, IF1SW-F4	Afu3g12890:c.-364G>C		-	Cluster_9_hexadehydroastechrome
Chr3	3425793	SNV	A	G	1	ISSFT-021, IF1SW-F4	Afu3g12890:c.-482T>C		-	Cluster_9_hexadehydroastechrome
Chr3	3426168	SNV	T	G	1	ISSFT-021, IF1SW-F4	Afu3g12900:c.-279T>G		-	Cluster_9_hexadehydroastechrome

Chr3	3426242	SNV	C	T	1	ISSFT-021	Afu3g12900:c.-205C>T		-	Cluster_9_hexadehydroastechrome
Chr3	3426295	SNV	G	A	1	ISSFT-021, IF1SW-F4	Afu3g12900:c.-152G>A		-	Cluster_9_hexadehydroastechrome
Chr3	3426474	SNV	G	T	1	ISSFT-021, IF1SW-F4	Afu3g12900:c.28G>T	Afu3g12900:p.Ala10Ser	Yes	Cluster_9_hexadehydroastechrome
Chr3	3426676	SNV	T	C	1	ISSFT-021, IF1SW-F4	Afu3g12900:c.180-22T>C		-	Cluster_9_hexadehydroastechrome
Chr3	3427129	SNV	T	C	1	CEA10, ISSFT-021, IF1SW-F4	Afu3g12900:c.552T>C		No	Cluster_9_hexadehydroastechrome
Chr3	3428231^3428232	Insertion	-	AT	2	CEA10			-	Cluster_9_hexadehydroastechrome
Chr3	3429765	SNV	C	T	1	ISSFT-021	Afu3g12910:c.94G>A	Afu3g12910:p.Glu32Lys	Yes	Cluster_9_hexadehydroastechrome
Chr3	3430521	SNV	T	C	1	CEA10, ISSFT-021, IF1SW-F4	Afu3g12920:c.328T>C	Afu3g12920:p.Ser110Pro	Yes	Cluster_9_hexadehydroastechrome
Chr3	3432321	SNV	A	G	1	CEA10, ISSFT-021, IF1SW-F4	Afu3g12920:c.2074A>G	Afu3g12920:p.Thr692Ala	Yes	Cluster_9_hexadehydroastechrome
Chr3	3433250	SNV	G	A	1	ISSFT-021, IF1SW-F4	Afu3g12920:c.3003G>A		No	Cluster_9_hexadehydroastechrome
Chr3	3435059	SNV	G	T	1	IF1SW-F4	Afu3g12920:c.4745G>T	Afu3g12920:p.Arg1582Leu	Yes	Cluster_9_hexadehydroastechrome
Chr3	3435792	SNV	T	C	1	ISSFT-021, IF1SW-F4	Afu3g12920:c.5478T>C		No	Cluster_9_hexadehydroastechrome
Chr3	3436479	SNV	T	C	1	CEA10	Afu3g12920:c.6165T>C		No	Cluster_9_hexadehydroastechrome
Chr3	3437543	SNV	A	C	1	ISSFT-021, IF1SW-F4			-	Cluster_9_hexadehydroastechrome
Chr3	3439253	SNV	G	A	1	IF1SW-F4			-	Cluster_9_hexadehydroastechrome
Chr3	3441432	SNV	A	G	1	CEA10, ISSFT-021, IF1SW-F4	Afu3g12940:c.386T>C	Afu3g12940:p.Leu129Pro	Yes	Cluster_9_hexadehydroastechrome
Chr3	3441910	SNV	A	T	1	CEA10, ISSFT-021, IF1SW-F4	Afu3g12940:c.-93T>A		-	Cluster_9_hexadehydroastechrome
Chr3	3441910..3441911	MNV	AA	TG	2	CEA10	Afu3g12940:c.-94_-93delTTinsCA		-	Cluster_9_hexadehydroastechrome
Chr3	3442839	SNV	A	G	1	CEA10, ISSFT-021, IF1SW-F4	Afu3g12950:c.862T>C	Afu3g12950:p.Ser288Pro	Yes	Cluster_9_hexadehydroastechrome
Chr3	3443263	SNV	A	C	1	CEA10, ISSFT-021, IF1SW-F4	Afu3g12950:c.492T>G		No	Cluster_9_hexadehydroastechrome
Chr3	3444128	SNV	G	A	1	IF1SW-F4	Afu3g12960:c.*76C>T		-	Cluster_9_hexadehydroastechrome
Chr3	3445665	SNV	T	C	1	CEA10, ISSFT-021, IF1SW-F4	Afu3g12960:c.377+37A>G		-	Cluster_9_hexadehydroastechrome
Chr3	3446008	SNV	C	A	1	CEA10, ISSFT-021, IF1SW-F4	Afu3g12960:c.122G>T	Afu3g12960:p.Gly41Val	Yes	Cluster_9_hexadehydroastechrome
Chr3	3593463	SNV	C	T	1	CEA10	Afu3g13600:c.1014C>T		No	Cluster_10_NRPS
Chr3	3594671	SNV	G	T	1	CEA10, ISSFT-021, IF1SW-F4			-	Cluster_10_NRPS
Chr3	3594836	SNV	C	T	1	CEA10, ISSFT-021, IF1SW-F4			-	Cluster_10_NRPS
Chr3	3595642	SNV	G	T	1	CEA10	Afu3g13610:c.792G>T		No	Cluster_10_NRPS
Chr3	3596895	SNV	C	T	1	CEA10, ISSFT-021, IF1SW-F4			-	Cluster_10_NRPS
Chr3	3598036	SNV	T	G	1	ISSFT-021, IF1SW-F4			-	Cluster_10_NRPS
Chr3	3599158	SNV	C	T	1	CEA10, ISSFT-021, IF1SW-F4			-	Cluster_10_NRPS
Chr3	3599735	SNV	A	T	1	ISSFT-021, IF1SW-F4			-	Cluster_10_NRPS
Chr3	3599815..3599816	MNV	GG	TA	2	ISSFT-021			-	Cluster_10_NRPS
Chr3	3599877	SNV	A	C	1	CEA10, ISSFT-021, IF1SW-F4			-	Cluster_10_NRPS
Chr3	3600868	SNV	C	T	1	IF1SW-F4	Afu3g13640:c.*124G>A		-	Cluster_10_NRPS
Chr3	3601484	SNV	A	G	1	ISSFT-021, IF1SW-F4	Afu3g13640:c.324T>C		No	Cluster_10_NRPS
Chr3	3601548	SNV	T	C	1	ISSFT-021, IF1SW-F4	Afu3g13640:c.321+4A>G		-	Cluster_10_NRPS
Chr3	3601636	SNV	A	G	1	ISSFT-021, IF1SW-F4	Afu3g13640:c.237T>C		No	Cluster_10_NRPS
Chr3	3601744	SNV	A	G	1	ISSFT-021, IF1SW-F4	Afu3g13640:c.129T>C		No	Cluster_10_NRPS
Chr3	3601807	SNV	A	G	1	ISSFT-021, IF1SW-F4	Afu3g13640:c.66T>C		No	Cluster_10_NRPS
Chr3	3602661	SNV	G	A	1	IF1SW-F4			-	Cluster_10_NRPS
Chr3	3602770	SNV	G	A	1	ISSFT-021			-	Cluster_10_NRPS
Chr3	3604060	SNV	G	A	1	ISSFT-021, IF1SW-F4			-	Cluster_10_NRPS
Chr3	3604238	SNV	C	T	1	ISSFT-021	Afu3g13660:c.657G>A		No	Cluster_10_NRPS
Chr3	3604513	SNV	A	G	1	CEA10	Afu3g13660:c.435T>C		No	Cluster_10_NRPS
Chr3	3604870	SNV	G	A	1	ISSFT-021, IF1SW-F4	Afu3g13660:c.78C>T		No	Cluster_10_NRPS
Chr3	3604950	SNV	G	T	1	CEA10			-	Cluster_10_NRPS
Chr3	3605153	SNV	G	C	1	ISSFT-021, IF1SW-F4			-	Cluster_10_NRPS
Chr3	3605371^3605372	Insertion	-	CCAA	4	ISSFT-021, IF1SW-F4			-	Cluster_10_NRPS
Chr3	3605436	SNV	A	G	1	CEA10			-	Cluster_10_NRPS
Chr3	3605823	SNV	C	T	1	CEA10, ISSFT-021, IF1SW-F4			-	Cluster_10_NRPS
Chr3	3606378	SNV	G	T	1	ISSFT-021	Afu3g13670:c.409G>T	Afu3g13670:p.Val137Phe	Yes	Cluster_10_NRPS
Chr3	3607482	SNV	T	C	1	CEA10, ISSFT-021, IF1SW-F4	Afu3g13670:c.1513T>C		No	Cluster_10_NRPS
Chr3	3607527	SNV	G	A	1	CEA10, ISSFT-021, IF1SW-F4	Afu3g13670:c.1558G>A	Afu3g13670:p.Val520Ile	Yes	Cluster_10_NRPS
Chr3	3607550	SNV	G	C	1	ISSFT-021, IF1SW-F4	Afu3g13670:c.1581G>C		No	Cluster_10_NRPS
Chr3	3608839	SNV	A	G	1	CEA10	Afu3g13680:c.1182T>C		No	Cluster_10_NRPS
Chr3	3608864	SNV	G	T	1	ISSFT-021	Afu3g13680:c.1157C>A	Afu3g13680:p.Pro386Gln	Yes	Cluster_10_NRPS
Chr3	3609333	SNV	C	G	1	CEA10	Afu3g13680:c.688G>C	Afu3g13680:p.Val230Leu	Yes	Cluster_10_NRPS
Chr3	3609378	SNV	T	G	1	CEA10	Afu3g13680:c.643A>C	Afu3g13680:p.Thr215Pro	Yes	Cluster_10_NRPS
Chr3	3609670	SNV	T	G	1	CEA10	Afu3g13680:c.351A>C		No	Cluster_10_NRPS

Chr3	3610212	SNV	A	T	1	ISSFT-021, IF1SW-F4			-	Cluster_10_NRPS
Chr3	3610235	SNV	T	A	1	IF1SW-F4			-	Cluster_10_NRPS
Chr3	3610389	SNV	C	A	1	CEA10, ISSFT-021, IF1SW-F4			-	Cluster_10_NRPS
Chr3	3611915	SNV	A	G	1	CEA10, ISSFT-021, IF1SW-F4	Afu3g13690:c.1162A>G	Afu3g13690:p.Met388Val	Yes	Cluster_10_NRPS
Chr3	3611982	SNV	A	G	1	CEA10, ISSFT-021, IF1SW-F4	Afu3g13690:c.1229A>G	Afu3g13690:p.Glu410Gly	Yes	Cluster_10_NRPS
Chr3	3612094	SNV	A	G	1	CEA10, ISSFT-021, IF1SW-F4	Afu3g13690:c.1341A>G		No	Cluster_10_NRPS
Chr3	3612380	SNV	A	G	1	CEA10, ISSFT-021, IF1SW-F4			-	Cluster_10_NRPS
Chr3	3612674	SNV	C	G	1	CEA10, ISSFT-021, IF1SW-F4	Afu3g13700:c.102C>G		No	Cluster_10_NRPS
Chr3	3612781	SNV	C	G	1	CEA10, ISSFT-021, IF1SW-F4	Afu3g13700:c.209C>G	Afu3g13700:p.Ala70Gly	Yes	Cluster_10_NRPS
Chr3	3613407	SNV	G	A	1	ISSFT-021	Afu3g13700:c.762G>A		No	Cluster_10_NRPS
Chr3	3613522	SNV	T	C	1	CEA10, ISSFT-021, IF1SW-F4	Afu3g13700:c.844-3T>C		-	Cluster_10_NRPS
Chr3	3613940	SNV	C	A	1	CEA10, ISSFT-021, IF1SW-F4	Afu3g13700:c.1259C>A	Afu3g13700:p.Ala420Asp	Yes	Cluster_10_NRPS
Chr3	3614158	SNV	G	T	1	CEA10	Afu3g13700:c.1477G>T	Afu3g13700:p.Asp493Tyr	Yes	Cluster_10_NRPS
Chr3	3614565^3614566	Insertion	-	G	1	CEA10	Afu3g13700:c.1875+9_1875+10insG		-	Cluster_10_NRPS
Chr3	3615905	SNV	C	G	1	CEA10, ISSFT-021, IF1SW-F4			-	Cluster_10_NRPS
Chr3	3617376	SNV	C	G	1	ISSFT-021	Afu3g13720:c.*134G>C		-	Cluster_10_NRPS
Chr3	3618315	SNV	T	C	1	CEA10	Afu3g13720:c.273-67A>G		-	Cluster_10_NRPS
Chr3	3620312	SNV	C	T	1	ISSFT-021	Afu3g13730:c.992C>T	Afu3g13730:p.Thr331Ile	Yes	Cluster_10_NRPS
Chr3	3622035	SNV	T	C	1	CEA10, ISSFT-021, IF1SW-F4	Afu3g13730:c.2715T>C		No	Cluster_10_NRPS
Chr3	3622629	SNV	C	A	1	CEA10, ISSFT-021, IF1SW-F4	Afu3g13730:c.3309C>A		No	Cluster_10_NRPS
Chr3	3895452	SNV	C	T	1	ISSFT-021, IF1SW-F4	Afu3g14690:c.1357G>A	Afu3g14690:p.Gly453Ser	Yes	Cluster_11_sphingofungin
Chr3	3895516	SNV	A	G	1	ISSFT-021, IF1SW-F4	Afu3g14690:c.1293T>C		No	Cluster_11_sphingofungin
Chr3	3895618	SNV	C	G	1	ISSFT-021, IF1SW-F4	Afu3g14690:c.1191G>C		No	Cluster_11_sphingofungin
Chr3	3895764	SNV	T	C	1	ISSFT-021, IF1SW-F4	Afu3g14690:c.1045A>G	Afu3g14690:p.Asn349Asp	Yes	Cluster_11_sphingofungin
Chr3	3896638	SNV	A	G	1	ISSFT-021, IF1SW-F4	Afu3g14690:c.294T>C		No	Cluster_11_sphingofungin
Chr3	3896898	SNV	T	C	1	ISSFT-021, IF1SW-F4	Afu3g14690:c.34A>G	Afu3g14690:p.Ile12Val	Yes	Cluster_11_sphingofungin
Chr3	3896942	SNV	T	C	1	ISSFT-021, IF1SW-F4	Afu3g14690:c.-11A>G		-	Cluster_11_sphingofungin
Chr3	3897139	SNV	T	C	1	ISSFT-021, IF1SW-F4			-	Cluster_11_sphingofungin
Chr3	3897227	SNV	C	T	1	ISSFT-021, IF1SW-F4			-	Cluster_11_sphingofungin
Chr3	3897252	SNV	T	A	1	ISSFT-021, IF1SW-F4	Afu3g14700:c.2T>A	Afu3g14700:p.Met1?	Yes	Cluster_11_sphingofungin
Chr3	3897362	SNV	A	G	1	CEA10	Afu3g14700:c.112A>G	Afu3g14700:p.Asn38Asp	Yes	Cluster_11_sphingofungin
Chr3	3897562	SNV	C	A	1	ISSFT-021, IF1SW-F4	Afu3g14700:c.312C>A	Afu3g14700:p.His104Gln	Yes	Cluster_11_sphingofungin
Chr3	3897755	SNV	T	A	1	ISSFT-021, IF1SW-F4	Afu3g14700:c.505T>A	Afu3g14700:p.Phe169Ile	Yes	Cluster_11_sphingofungin
Chr3	3897957^3897958	Insertion	-	T	1	ISSFT-021, IF1SW-F4	Afu3g14700:c.692+15_692+16insT		-	Cluster_11_sphingofungin
Chr3	3898093	SNV	C	T	1	ISSFT-021, IF1SW-F4	Afu3g14700:c.781C>T		No	Cluster_11_sphingofungin
Chr3	3898183	SNV	G	A	1	CEA10, ISSFT-021, IF1SW-F4	Afu3g14700:c.871G>A	Afu3g14700:p.Ala291Thr	Yes	Cluster_11_sphingofungin
Chr3	3898260	SNV	C	T	1	ISSFT-021, IF1SW-F4	Afu3g14700:c.948C>T		No	Cluster_11_sphingofungin
Chr3	3898332	SNV	A	C	1	ISSFT-021, IF1SW-F4	Afu3g14700:c.1020A>C		No	Cluster_11_sphingofungin
Chr3	3898661	SNV	A	G	1	ISSFT-021, IF1SW-F4	Afu3g14700:c.1349A>G	Afu3g14700:p.Lys450Arg	Yes	Cluster_11_sphingofungin
Chr3	3898703	SNV	G	T	1	IF1SW-F4	Afu3g14700:c.1391G>T	Afu3g14700:p.Arg464Ile	Yes	Cluster_11_sphingofungin
Chr3	3898908	SNV	T	C	1	ISSFT-021, IF1SW-F4	Afu3g14700:c.1596T>C		No	Cluster_11_sphingofungin
Chr3	3899031	SNV	G	A	1	ISSFT-021, IF1SW-F4	Afu3g14700:c.1719G>A		No	Cluster_11_sphingofungin
Chr3	3899533	SNV	G	A	1	CEA10	Afu3g14700:c.2221G>A	Afu3g14700:p.Val741Ile	Yes	Cluster_11_sphingofungin
Chr3	3899543	SNV	C	T	1	ISSFT-021	Afu3g14700:c.2231C>T	Afu3g14700:p.Ser744Leu	Yes	Cluster_11_sphingofungin
Chr3	3899687	SNV	A	G	1	ISSFT-021, IF1SW-F4	Afu3g14700:c.2375A>G	Afu3g14700:p.Asn792Ser	Yes	Cluster_11_sphingofungin
Chr3	3899716..3899720	MNV	GCGCT	ACGCG	5	ISSFT-021, IF1SW-F4	u3g14700:c.2404_2408delGCGCTinsACG	3g14700:p.Ala802_Leu803delinsThr	Yes	Cluster_11_sphingofungin
Chr3	3900259	SNV	A	G	1	CEA10	Afu3g14700:c.2947A>G	Afu3g14700:p.Lys983Glu	Yes	Cluster_11_sphingofungin
Chr3	3901345	SNV	C	T	1	ISSFT-021, IF1SW-F4	Afu3g14700:c.4033C>T	Afu3g14700:p.Pro1345Ser	Yes	Cluster_11_sphingofungin
Chr3	3901966	SNV	C	A	1	CEA10	Afu3g14700:c.4654C>A	Afu3g14700:p.Leu1552Ile	Yes	Cluster_11_sphingofungin
Chr3	3902207	SNV	C	T	1	ISSFT-021, IF1SW-F4	Afu3g14700:c.4895C>T	Afu3g14700:p.Ser1632Leu	Yes	Cluster_11_sphingofungin
Chr3	3902869	SNV	T	C	1	CEA10	Afu3g14700:c.5501T>C	Afu3g14700:p.Ile1834Thr	Yes	Cluster_11_sphingofungin
Chr3	3902992	SNV	A	G	1	CEA10, ISSFT-021, IF1SW-F4	Afu3g14700:c.5624A>G	Afu3g14700:p.Gln1875Arg	Yes	Cluster_11_sphingofungin
Chr3	3903368	SNV	A	C	1	CEA10	Afu3g14700:c.5928A>C	Afu3g14700:p.Lys1976Asn	Yes	Cluster_11_sphingofungin
Chr3	3903437	SNV	T	G	1	ISSFT-021, IF1SW-F4	Afu3g14700:c.5997T>G		No	Cluster_11_sphingofungin
Chr3	3903647	SNV	C	T	1	IF1SW-F4	Afu3g14700:c.6207C>T		No	Cluster_11_sphingofungin
Chr3	3903815	SNV	C	T	1	IF1SW-F4	Afu3g14700:c.6375C>T		No	Cluster_11_sphingofungin
Chr3	3903963	SNV	A	C	1	IF1SW-F4	Afu3g14700:c.6523A>C		No	Cluster_11_sphingofungin
Chr3	3908809	SNV	T	C	1	CEA10, ISSFT-021	Afu3g14720:c.-280A>G		-	Cluster_11_sphingofungin
Chr3	3909676	SNV	T	G	1	CEA10, ISSFT-021	Afu3g14730:c.543T>G		No	Cluster_11_sphingofungin

Chr3	3910235	SNV	G	C	1	ISSFT-021	Afu3g14730:c.1102G>C	Afu3g14730:p.Val368Leu	Yes	Cluster_11_sphingofungin
Chr3	3910438	SNV	G	C	1	CEA10, ISSFT-021, IF1SW-F4	Afu3g14730:c.1305G>C	Afu3g14730:p.Leu435Phe	Yes	Cluster_11_sphingofungin
Chr3	3910783	SNV	T	C	1	IF1SW-F4			-	Cluster_11_sphingofungin
Chr3	3910826	SNV	T	C	1	IF1SW-F4	Afu3g14740:c.1239A>G		No	Cluster_11_sphingofungin
Chr3	3910948	SNV	C	T	1	IF1SW-F4	Afu3g14740:c.1117G>A	Afu3g14740:p.Glu373Lys	Yes	Cluster_11_sphingofungin
Chr3	3910957	SNV	T	C	1	IF1SW-F4	Afu3g14740:c.1108A>G	Afu3g14740:p.Ile370Val	Yes	Cluster_11_sphingofungin
Chr3	3911275	SNV	A	G	1	CEA10, ISSFT-021, IF1SW-F4	Afu3g14740:c.822-32T>C		-	Cluster_11_sphingofungin
Chr3	3911309	SNV	G	A	1	CEA10, ISSFT-021, IF1SW-F4	Afu3g14740:c.821+22C>T		-	Cluster_11_sphingofungin
Chr3	3912396	SNV	A	C	1	CEA10, ISSFT-021, IF1SW-F4			-	Cluster_11_sphingofungin
Chr3	3912845	SNV	A	G	1	ISSFT-021, IF1SW-F4			-	Cluster_11_sphingofungin
Chr3	3913123	SNV	G	A	1	CEA10, ISSFT-021, IF1SW-F4			-	Cluster_11_sphingofungin
Chr3	3913573	SNV	C	T	1	ISSFT-021, IF1SW-F4			-	Cluster_11_sphingofungin
Chr3	3915063	SNV	G	A	1	CEA10	Afu3g14750:c.988G>A	Afu3g14750:p.Asp330Asn	Yes	Cluster_11_sphingofungin
Chr3	3915419	SNV	A	G	1	IF1SW-F4	Afu3g14750:c.1344A>G		No	Cluster_11_sphingofungin
Chr3	3915666	SNV	A	G	1	ISSFT-021	Afu3g14750:c.1591A>G	Afu3g14750:p.Ile531Val	Yes	Cluster_11_sphingofungin
Chr3	3915743	SNV	C	T	1	CEA10	Afu3g14750:c.1668C>T		No	Cluster_11_sphingofungin
Chr3	3915864	SNV	G	A	1	CEA10, IF1SW-F4	Afu3g14750:c.1751+38G>A		-	Cluster_11_sphingofungin
Chr3	3916732	SNV	T	C	1	CEA10, ISSFT-021, IF1SW-F4			-	Cluster_11_sphingofungin
Chr3	3917291	SNV	C	T	1	CEA10, ISSFT-021, IF1SW-F4	Afu3g14760:c.318C>T		No	Cluster_11_sphingofungin
Chr3	3917474	SNV	C	A	1	CEA10, IF1SW-F4	Afu3g14760:c.477+24C>A		-	Cluster_11_sphingofungin
Chr3	3917616	SNV	C	G	1	CEA10, ISSFT-021	Afu3g14760:c.582C>G	Afu3g14760:p.Asn194Lys	Yes	Cluster_11_sphingofungin
Chr3	3917658	SNV	A	C	1	ISSFT-021	Afu3g14760:c.624A>C	Afu3g14760:p.Gln208His	Yes	Cluster_11_sphingofungin
Chr3	3917759	SNV	G	A	1	CEA10, ISSFT-021, IF1SW-F4	Afu3g14760:c.725G>A	Afu3g14760:p.Arg242Gln	Yes	Cluster_11_sphingofungin
Chr3	3918025	SNV	G	A	1	CEA10	Afu3g14760:c.991G>A	Afu3g14760:p.Val331Ile	Yes	Cluster_11_sphingofungin
Chr3	3918313	SNV	C	T	1	IF1SW-F4	Afu3g14760:c.1279C>T	Afu3g14760:p.Pro427Ser	Yes	Cluster_11_sphingofungin
Chr3	4008296	SNV	A	G	1	CEA10, ISSFT-021, IF1SW-F4	Afu3g15250:c.510A>G		No	Cluster_12_NRPS
Chr3	4008533	SNV	G	A	1	CEA10, ISSFT-021, IF1SW-F4	Afu3g15250:c.747G>A		No	Cluster_12_NRPS
Chr3	4009551	SNV	T	C	1	CEA10, ISSFT-021, IF1SW-F4	Afu3g15250:c.*64T>C		-	Cluster_12_NRPS
Chr3	4009576	SNV	C	T	1	CEA10, ISSFT-021, IF1SW-F4	Afu3g15250:c.*89C>T		-	Cluster_12_NRPS
Chr3	4009646	SNV	T	C	1	CEA10, ISSFT-021, IF1SW-F4	Afu3g15250:c.*159T>C		-	Cluster_12_NRPS
Chr3	4011052	SNV	T	G	1	CEA10, ISSFT-021, IF1SW-F4	Afu3g15270:c.531T>G	Afu3g15270:p.Ile177Met	Yes	Cluster_12_NRPS
Chr3	4011853	SNV	A	G	1	CEA10, ISSFT-021, IF1SW-F4	Afu3g15270:c.1332A>G		No	Cluster_12_NRPS
Chr3	4012157	SNV	C	T	1	ISSFT-021, IF1SW-F4	Afu3g15270:c.1636C>T	Afu3g15270:p.Leu546Phe	Yes	Cluster_12_NRPS
Chr3	4012378	SNV	G	A	1	ISSFT-021, IF1SW-F4	Afu3g15270:c.1857G>A		No	Cluster_12_NRPS
Chr3	4012588	SNV	A	G	1	CEA10, ISSFT-021, IF1SW-F4	Afu3g15270:c.2067A>G		No	Cluster_12_NRPS
Chr3	4012733	SNV	T	C	1	CEA10, ISSFT-021, IF1SW-F4	Afu3g15270:c.2212T>C	Afu3g15270:p.Ser738Pro	Yes	Cluster_12_NRPS
Chr3	4012844	SNV	A	G	1	CEA10, ISSFT-021, IF1SW-F4	Afu3g15270:c.2323A>G	Afu3g15270:p.Lys775Glu	Yes	Cluster_12_NRPS
Chr3	4013023	SNV	G	A	1	CEA10	Afu3g15270:c.2502G>A		No	Cluster_12_NRPS
Chr3	4013119	SNV	G	T	1	ISSFT-021, IF1SW-F4	Afu3g15270:c.2598G>T		No	Cluster_12_NRPS
Chr3	4013246	SNV	A	G	1	ISSFT-021, IF1SW-F4	Afu3g15270:c.2725A>G	Afu3g15270:p.Ile909Val	Yes	Cluster_12_NRPS
Chr3	4013346	SNV	A	T	1	ISSFT-021, IF1SW-F4	Afu3g15270:c.2825A>T	Afu3g15270:p.Tyr942Phe	Yes	Cluster_12_NRPS
Chr3	4013895	SNV	T	C	1	CEA10, ISSFT-021, IF1SW-F4	Afu3g15270:c.3374T>C	Afu3g15270:p.Val1125Ala	Yes	Cluster_12_NRPS
Chr3	4014252	SNV	G	A	1	CEA10, ISSFT-021, IF1SW-F4	Afu3g15270:c.3731G>A	Afu3g15270:p.Cys1244Tyr	Yes	Cluster_12_NRPS
Chr3	4015561	SNV	C	G	1	ISSFT-021, IF1SW-F4	Afu3g15270:c.4986C>G	Afu3g15270:p.Asn1662Lys	Yes	Cluster_12_NRPS
Chr3	4017241	SNV	A	G	1	ISSFT-021, IF1SW-F4	Afu3g15270:c.6666A>G	Afu3g15270:p.Ile2222Met	Yes	Cluster_12_NRPS
Chr3	4017496	SNV	G	A	1	ISSFT-021	Afu3g15270:c.6921G>A		No	Cluster_12_NRPS
Chr3	4017514	SNV	T	G	1	CEA10	Afu3g15270:c.6939T>G		No	Cluster_12_NRPS
Chr3	4017781	SNV	G	A	1	ISSFT-021, IF1SW-F4			-	Cluster_12_NRPS
Chr3	4018801	SNV	G	T	1	CEA10	Afu3g15280:c.287C>A	Afu3g15280:p.Pro96His	Yes	Cluster_12_NRPS
Chr3	4019748	SNV	G	T	1	ISSFT-021, IF1SW-F4			-	Cluster_12_NRPS
Chr3	4019820..4019823	MNV	ATCA	GTCG	4	ISSFT-021, IF1SW-F4			-	Cluster_12_NRPS
Chr3	4020189	SNV	A	G	1	ISSFT-021, IF1SW-F4			-	Cluster_12_NRPS
Chr3	4020278	SNV	T	C	1	CEA10, ISSFT-021, IF1SW-F4			-	Cluster_12_NRPS
Chr3	4020981	SNV	G	A	1	ISSFT-021, IF1SW-F4			-	Cluster_12_NRPS
Chr3	4021471	SNV	T	A	1	ISSFT-021, IF1SW-F4	Afu3g15290:c.185T>A	Afu3g15290:p.Leu62Gln	Yes	Cluster_12_NRPS
Chr3	4021751	SNV	C	A	1	IF1SW-F4	Afu3g15290:c.465C>A	Afu3g15290:p.Asp155Glu	Yes	Cluster_12_NRPS
Chr3	4022642	SNV	C	T	1	ISSFT-021, IF1SW-F4	Afu3g15290:c.1304C>T	Afu3g15290:p.Thr435Met	Yes	Cluster_12_NRPS
Chr3	4023789	SNV	C	G	1	ISSFT-021, IF1SW-F4			-	Cluster_12_NRPS
Chr3	4024843	SNV	A	G	1	ISSFT-021			-	Cluster_12_NRPS

Chr3	4024858	SNV	T	C	1	CEA10			-	Cluster_12_NRPS
Chr3	4025304	SNV	A	G	1	CEA10			-	Cluster_12_NRPS
Chr3	4025429	SNV	C	T	1	ISSFT-021			-	Cluster_12_NRPS
Chr3	4025643	SNV	T	C	1	CEA10			-	Cluster_12_NRPS
Chr3	4025819	SNV	A	G	1	CEA10			-	Cluster_12_NRPS
Chr3	4025829	SNV	A	G	1	CEA10			-	Cluster_12_NRPS
Chr3	4026282	SNV	A	G	1	CEA10			-	Cluster_12_NRPS
Chr3	4026508	SNV	A	G	1	CEA10			-	Cluster_12_NRPS
Chr3	4026526	SNV	A	G	1	CEA10			-	Cluster_12_NRPS
Chr3	4026843^4026844	Insertion	-	CAAGGACC	12	ISSFT-021, IF1SW-F4			-	Cluster_12_NRPS
Chr3	4026955	SNV	G	A	1	ISSFT-021			-	Cluster_12_NRPS
Chr3	4027166	SNV	T	A	1	ISSFT-021, IF1SW-F4			-	Cluster_12_NRPS
Chr3	4027688	SNV	C	T	1	ISSFT-021, IF1SW-F4	Afu3g15300:c.512-26G>A		-	Cluster_12_NRPS
Chr3	4027937	SNV	A	G	1	ISSFT-021, IF1SW-F4	Afu3g15300:c.390T>C		No	Cluster_12_NRPS
Chr3	4028220	SNV	A	G	1	ISSFT-021, IF1SW-F4	Afu3g15300:c.107T>C	Afu3g15300:p.Leu36Pro	Yes	Cluster_12_NRPS
Chr4	56164	SNV	C	T	1	IF1SW-F4	Afu4g00210:c.5152G>A	Afu4g00210:p.Glu1718Lys	Yes	Cluster_13_endocrocin
Chr4	56577	SNV	T	C	1	IF1SW-F4	Afu4g00210:c.4739A>G	Afu4g00210:p.Lys1580Arg	Yes	Cluster_13_endocrocin
Chr4	57108	SNV	C	T	1	CEA10, ISSFT-021, IF1SW-F4	Afu4g00210:c.4208G>A	Afu4g00210:p.Arg1403Gln	Yes	Cluster_13_endocrocin
Chr4	57510^57511	Insertion	-	GCT	3	CEA10, ISSFT-021	Afu4g00210:c.3863_3864insAGC	fu4g00210:p.Ala1292_Ser1293insA	Yes	Cluster_13_endocrocin
Chr4	60348	SNV	T	G	1	CEA10, ISSFT-021, IF1SW-F4	Afu4g00210:c.1026A>C	Afu4g00210:p.Gln342His	Yes	Cluster_13_endocrocin
Chr4	60600	SNV	A	G	1	IF1SW-F4	Afu4g00210:c.774T>C		No	Cluster_13_endocrocin
Chr4	60693	SNV	T	G	1	ISSFT-021, IF1SW-F4	Afu4g00210:c.681A>C		No	Cluster_13_endocrocin
Chr4	61020	SNV	A	G	1	CEA10	Afu4g00210:c.405T>C		No	Cluster_13_endocrocin
Chr4	61087	SNV	G	A	1	CEA10, ISSFT-021, IF1SW-F4	Afu4g00210:c.338C>T	Afu4g00210:p.Pro113Leu	Yes	Cluster_13_endocrocin
Chr4	61122	SNV	C	T	1	CEA10	Afu4g00210:c.315-12G>A		-	Cluster_13_endocrocin
Chr4	61300	SNV	C	T	1	CEA10	Afu4g00210:c.186G>A		No	Cluster_13_endocrocin
Chr4	61526	SNV	A	G	1	ISSFT-021, IF1SW-F4			-	Cluster_13_endocrocin
Chr4	61587	SNV	C	G	1	CEA10			-	Cluster_13_endocrocin
Chr4	62084	SNV	A	G	1	CEA10, ISSFT-021, IF1SW-F4	Afu4g00220:c.714T>C		No	Cluster_13_endocrocin
Chr4	62526	SNV	A	G	1	ISSFT-021	Afu4g00220:c.272T>C	Afu4g00220:p.Val91Ala	Yes	Cluster_13_endocrocin
Chr4	62651	SNV	C	T	1	CEA10, ISSFT-021, IF1SW-F4	Afu4g00220:c.147G>A		No	Cluster_13_endocrocin
Chr4	62696	SNV	G	A	1	CEA10, ISSFT-021, IF1SW-F4	Afu4g00220:c.132+29C>T		-	Cluster_13_endocrocin
Chr4	63052	SNV	A	T	1	CEA10, ISSFT-021, IF1SW-F4			-	Cluster_13_endocrocin
Chr4	63773	SNV	A	T	1	CEA10	Afu4g00225:c.524A>T	Afu4g00225:p.Tyr175Phe	Yes	Cluster_13_endocrocin
Chr4	63977	SNV	T	G	1	CEA10, ISSFT-021, IF1SW-F4			-	Cluster_13_endocrocin
Chr4	64001	SNV	A	G	1	CEA10, ISSFT-021, IF1SW-F4			-	Cluster_13_endocrocin
Chr4	64254	SNV	C	G	1	CEA10, ISSFT-021, IF1SW-F4	Afu4g00230:c.861G>C		No	Cluster_13_endocrocin
Chr4	3827491	SNV	A	G	1	ISSFT-021			-	Cluster_14_trypacidin
Chr4	3827499	SNV	G	A	1	CEA10			-	Cluster_14_trypacidin
Chr4	3827614	SNV	A	C	1	ISSFT-021			-	Cluster_14_trypacidin
Chr4	3827662	SNV	T	C	1	ISSFT-021			-	Cluster_14_trypacidin
Chr4	3827699	SNV	C	T	1	ISSFT-021			-	Cluster_14_trypacidin
Chr4	3827727	SNV	G	A	1	ISSFT-021			-	Cluster_14_trypacidin
Chr4	3828014	SNV	C	G	1	CEA10, ISSFT-021, IF1SW-F4	Afu4g14470:c.231C>G		No	Cluster_14_trypacidin
Chr4	3828242	SNV	T	A	1	ISSFT-021	Afu4g14470:c.408T>A		No	Cluster_14_trypacidin
Chr4	3828476	SNV	G	C	1	CEA10			-	Cluster_14_trypacidin
Chr4	3828551	SNV	G	C	1	CEA10, ISSFT-021	Afu4g14490:c.46G>C	Afu4g14490:p.Ala16Pro	Yes	Cluster_14_trypacidin
Chr4	3829116^3829117	Insertion	-	TCG	3	ISSFT-021	Afu4g14490:c.426+26_427-26insTCG		-	Cluster_14_trypacidin
Chr4	3829136	SNV	A	G	1	ISSFT-021	Afu4g14490:c.427-7A>G		-	Cluster_14_trypacidin
Chr4	3829243	SNV	C	T	1	CEA10, ISSFT-021	Afu4g14490:c.518+9C>T		-	Cluster_14_trypacidin
Chr4	3829306	SNV	A	C	1	ISSFT-021	Afu4g14490:c.532+19A>C		-	Cluster_14_trypacidin
Chr4	3829358	SNV	G	A	1	ISSFT-021	Afu4g14490:c.555G>A		No	Cluster_14_trypacidin
Chr4	3829466	SNV	T	A	1	ISSFT-021	Afu4g14490:c.663T>A		No	Cluster_14_trypacidin
Chr4	3829593	SNV	T	C	1	ISSFT-021	Afu4g14490:c.790T>C	Afu4g14490:p.Phe264Leu	Yes	Cluster_14_trypacidin
Chr4	3829890	SNV	A	G	1	ISSFT-021	Afu4g14490:c.1087A>G	Afu4g14490:p.Ser363Gly	Yes	Cluster_14_trypacidin
Chr4	3830123	SNV	C	A	1	IF1SW-F4	Afu4g14490:c.1320C>A		No	Cluster_14_trypacidin
Chr4	3830795	SNV	C	G	1	CEA10			-	Cluster_14_trypacidin
Chr4	3831200	SNV	G	A	1	CEA10	Afu4g14500:c.78G>A		No	Cluster_14_trypacidin
Chr4	3832364	SNV	A	G	1	CEA10	Afu4g14500:c.1242A>G		No	Cluster_14_trypacidin

Chr4	3833172	SNV	C	T	1	CEA10, ISSFT-021, IF1SW-F4	Afu4g14510:c.406+3G>A		-	Cluster_14_trypacidin
Chr4	3834620	SNV	T	C	1	CEA10, ISSFT-021, IF1SW-F4	Afu4g14520:c.390T>C		No	Cluster_14_trypacidin
Chr4	3835092	SNV	A	C	1	CEA10, IF1SW-F4	Afu4g14520:c.*70A>C		-	Cluster_14_trypacidin
Chr4	3835315	SNV	A	C	1	CEA10, IF1SW-F4			-	Cluster_14_trypacidin
Chr4	3835385	SNV	G	A	1	CEA10, ISSFT-021, IF1SW-F4			-	Cluster_14_trypacidin
Chr4	3835478	SNV	T	C	1	CEA10, IF1SW-F4	Afu4g14530:c.39T>C		No	Cluster_14_trypacidin
Chr4	3835536	SNV	G	A	1	CEA10, IF1SW-F4	Afu4g14530:c.97G>A	Afu4g14530:p.Val33Ile	Yes	Cluster_14_trypacidin
Chr4	3837471	SNV	G	A	1	CEA10, ISSFT-021, IF1SW-F4			-	Cluster_14_trypacidin
Chr4	3837471..3837474	MNV	GGCA	AGCC	4	CEA10			-	Cluster_14_trypacidin
Chr4	3837579	SNV	T	C	1	CEA10			-	Cluster_14_trypacidin
Chr4	3837580	SNV	A	T	1	IF1SW-F4			-	Cluster_14_trypacidin
Chr4	3837860	SNV	T	G	1	CEA10			-	Cluster_14_trypacidin
Chr4	3837961..3837980	Deletion	TGACCCAAGGC	-	20	ISSFT-021, IF1SW-F4			-	Cluster_14_trypacidin
Chr4	3838175	SNV	T	A	1	CEA10, ISSFT-021, IF1SW-F4			-	Cluster_14_trypacidin
Chr4	3838477	SNV	G	A	1	CEA10			-	Cluster_14_trypacidin
Chr4	3838749	SNV	A	G	1	CEA10, ISSFT-021, IF1SW-F4			-	Cluster_14_trypacidin
Chr4	3839095	SNV	T	C	1	CEA10, ISSFT-021, IF1SW-F4	Afu4g14550:c.988A>G	Afu4g14550:p.Thr330Ala	Yes	Cluster_14_trypacidin
Chr4	3839471	SNV	G	A	1	CEA10, ISSFT-021, IF1SW-F4	Afu4g14550:c.664C>T	Afu4g14550:p.His222Tyr	Yes	Cluster_14_trypacidin
Chr4	3839799	SNV	G	A	1	CEA10, ISSFT-021, IF1SW-F4	Afu4g14550:c.336C>T		No	Cluster_14_trypacidin
Chr4	3840421	SNV	C	G	1	CEA10			-	Cluster_14_trypacidin
Chr4	3840452	SNV	C	T	1	CEA10	Afu4g14560:c.5337G>A		No	Cluster_14_trypacidin
Chr4	3840542	SNV	C	T	1	CEA10, ISSFT-021, IF1SW-F4	Afu4g14560:c.5247G>A		No	Cluster_14_trypacidin
Chr4	3841328	SNV	A	G	1	IF1SW-F4	Afu4g14560:c.4461T>C		No	Cluster_14_trypacidin
Chr4	3841443	SNV	T	C	1	IF1SW-F4	Afu4g14560:c.4346A>G	Afu4g14560:p.Asn1449Ser	Yes	Cluster_14_trypacidin
Chr4	3842091^3842092	Insertion	-	T	1	CEA10	Afu4g14560:c.3742_3743insA	Afu4g14560:p.Asp1248fs	Yes	Cluster_14_trypacidin
Chr4	3844829	SNV	C	A	1	ISSFT-021, IF1SW-F4	Afu4g14560:c.1051G>T	Afu4g14560:p.Val351Leu	Yes	Cluster_14_trypacidin
Chr4	3845941	SNV	A	G	1	CEA10, ISSFT-021, IF1SW-F4	Afu4g14560:c.30T>C		No	Cluster_14_trypacidin
Chr4	3845971	SNV	C	T	1	CEA10, ISSFT-021, IF1SW-F4			-	Cluster_14_trypacidin
Chr4	3846019	SNV	A	G	1	CEA10, ISSFT-021, IF1SW-F4			-	Cluster_14_trypacidin
Chr4	3846079	SNV	G	A	1	CEA10, ISSFT-021, IF1SW-F4			-	Cluster_14_trypacidin
Chr4	3847165..3847166	MNV	AC	GA	2	CEA10, ISSFT-021, IF1SW-F4	Afu4g14570:c.1057_1058delACinsGA	Afu4g14570:p.Thr353Asp	Yes	Cluster_14_trypacidin
Chr4	3847407	SNV	A	C	1	CEA10, ISSFT-021, IF1SW-F4			-	Cluster_14_trypacidin
Chr4	3847477	SNV	T	C	1	CEA10, ISSFT-021, IF1SW-F4			-	Cluster_14_trypacidin
Chr4	3847483	SNV	T	A	1	CEA10, ISSFT-021, IF1SW-F4			-	Cluster_14_trypacidin
Chr4	3847554	SNV	T	G	1	CEA10, ISSFT-021, IF1SW-F4			-	Cluster_14_trypacidin
Chr4	3847807	SNV	G	A	1	CEA10, ISSFT-021, IF1SW-F4	Afu4g14580:c.33G>A		No	Cluster_14_trypacidin
Chr4	3848140	SNV	C	T	1	IF1SW-F4	Afu4g14580:c.366C>T		No	Cluster_14_trypacidin
Chr4	3848203	SNV	T	C	1	IF1SW-F4	Afu4g14580:c.389-40T>C		-	Cluster_14_trypacidin
Chr4	3848277	SNV	T	C	1	IF1SW-F4	Afu4g14580:c.423T>C		No	Cluster_14_trypacidin
Chr4	3897699	SNV	G	A	1	CEA10, ISSFT-021	Afu4g14770:c.84C>T		No	Cluster_15_helvolic_acid
Chr4	3899722	SNV	T	C	1	ISSFT-021, IF1SW-F4	Afu4g14780:c.238A>G	Afu4g14780:p.Thr80Ala	Yes	Cluster_15_helvolic_acid
Chr4	3899948	SNV	G	T	1	CEA10, ISSFT-021, IF1SW-F4	Afu4g14780:c.12C>A	Afu4g14780:p.Tyr4*	Yes	Cluster_15_helvolic_acid
Chr4	3900976	SNV	C	A	1	CEA10, ISSFT-021, IF1SW-F4	Afu4g14790:c.439C>A		No	Cluster_15_helvolic_acid
Chr4	3900998	SNV	G	A	1	CEA10, ISSFT-021, IF1SW-F4	Afu4g14790:c.461G>A	Afu4g14790:p.Gly154Asp	Yes	Cluster_15_helvolic_acid
Chr4	3901111	SNV	T	C	1	CEA10, ISSFT-021, IF1SW-F4	Afu4g14790:c.574T>C	Afu4g14790:p.Trp192Arg	Yes	Cluster_15_helvolic_acid
Chr4	3901333	SNV	C	T	1	ISSFT-021	Afu4g14790:c.796C>T	Afu4g14790:p.Pro266Ser	Yes	Cluster_15_helvolic_acid
Chr4	3901761	SNV	A	G	1	CEA10, ISSFT-021, IF1SW-F4	Afu4g14790:c.1224A>G		No	Cluster_15_helvolic_acid
Chr4	3904674..3904677	Deletion	AGTG	-	4	CEA10, ISSFT-021, IF1SW-F4	Afu4g14810:c.1524_*3delAGTG	Afu4g14810:p.Val509fs	Yes	Cluster_15_helvolic_acid
Chr4	3906445	SNV	G	A	1	CEA10	Afu4g14820:c.1347G>A		No	Cluster_15_helvolic_acid
Chr4	3906706	SNV	C	T	1	CEA10, ISSFT-021, IF1SW-F4	Afu4g14830:c.-19C>T		-	Cluster_15_helvolic_acid
Chr4	3906922	SNV	G	C	1	CEA10, ISSFT-021, IF1SW-F4	Afu4g14830:c.198G>C		No	Cluster_15_helvolic_acid
Chr4	3907245	SNV	A	C	1	CEA10, ISSFT-021, IF1SW-F4	Afu4g14830:c.521A>C	Afu4g14830:p.Lys174Thr	Yes	Cluster_15_helvolic_acid
Chr4	3908511	SNV	T	A	1	ISSFT-021, IF1SW-F4			-	Cluster_15_helvolic_acid
Chr4	3909982	SNV	A	G	1	CEA10, ISSFT-021, IF1SW-F4	Afu4g14840:c.1342A>G	Afu4g14840:p.Met448Val	Yes	Cluster_15_helvolic_acid
Chr4	3911609	SNV	G	A	1	CEA10, ISSFT-021, IF1SW-F4	Afu4g14850:c.243C>T		No	Cluster_15_helvolic_acid
Chr5	2602733	SNV	A	C	1	CEA10	Afu5g10120:c.-277A>C		-	Cluster_16_NRPS
Chr5	2602753	SNV	A	C	1	CEA10	Afu5g10120:c.-257A>C		-	Cluster_16_NRPS
Chr5	2602781..2602783	MNV	AAT	GAC	3	CEA10	Afu5g10120:c.-229_-227delAATinsGAC		-	Cluster_16_NRPS
Chr5	2602900	SNV	T	G	1	CEA10	Afu5g10120:c.-180-6T>G		-	Cluster_16_NRPS

Chr5	2603081..2603084	Deletion	ACTC	-	4	CEA10	Afu5g10120:c.-5_-2delACTC		-	Cluster_16_NRPS
Chr5	2604074	SNV	T	C	1	CEA10, ISSFT-021, IF1SW-F4	Afu5g10120:c.989T>C	Afu5g10120:p.Leu330Pro	Yes	Cluster_16_NRPS
Chr5	2606981	SNV	T	C	1	IF1SW-F4			-	Cluster_16_NRPS
Chr5	2607991	SNV	G	T	1	CEA10, ISSFT-021	Afu5g10130:c.396G>T	Afu5g10130:p.Glu132Asp	Yes	Cluster_16_NRPS
Chr5	2608147	SNV	G	C	1	ISSFT-021	Afu5g10130:c.552G>C	Afu5g10130:p.Gln184His	Yes	Cluster_16_NRPS
Chr5	2608358	SNV	A	T	1	CEA10, ISSFT-021, IF1SW-F4	Afu5g10130:c.763A>T	Afu5g10130:p.Met255Leu	Yes	Cluster_16_NRPS
Chr5	2608487	SNV	C	T	1	CEA10, ISSFT-021, IF1SW-F4			-	Cluster_16_NRPS
Chr5	2608687	SNV	C	A	1	IF1SW-F4			-	Cluster_16_NRPS
Chr5	2608927^2608928	Insertion	-	GGA	3	CEA10			-	Cluster_16_NRPS
Chr5	2609600	SNV	G	A	1	ISSFT-021			-	Cluster_16_NRPS
Chr5	2609657	SNV	C	T	1	CEA10, ISSFT-021, IF1SW-F4			-	Cluster_16_NRPS
Chr5	2609923^2609924	Insertion	-	T	1	CEA10, ISSFT-021, IF1SW-F4			-	Cluster_16_NRPS
Chr5	2610405	SNV	T	C	1	CEA10, ISSFT-021, IF1SW-F4	Afu5g10140:c.653A>G	Afu5g10140:p.Asp218Gly	Yes	Cluster_16_NRPS
Chr5	2610961^2610962	Insertion	-	A	1	CEA10, ISSFT-021, IF1SW-F4	Afu5g10140:c.106-10_106-9insT		-	Cluster_16_NRPS
Chr5	2611779..2611780	MNV	CA	AG	2	ISSFT-021, IF1SW-F4			-	Cluster_16_NRPS
Chr5	2611997	SNV	G	C	1	ISSFT-021, IF1SW-F4			-	Cluster_16_NRPS
Chr5	2612713	SNV	C	G	1	CEA10	Afu5g10150:c.279C>G	Afu5g10150:p.Tyr93*	Yes	Cluster_16_NRPS
Chr5	2612798	SNV	T	C	1	CEA10, ISSFT-021, IF1SW-F4	Afu5g10150:c.364T>C	Afu5g10150:p.Cys122Arg	Yes	Cluster_16_NRPS
Chr5	2613079	SNV	A	C	1	CEA10, ISSFT-021, IF1SW-F4			-	Cluster_16_NRPS
Chr5	2614369^2614370	Insertion	-	G	1	CEA10, ISSFT-021, IF1SW-F4	Afu5g10160:c.252+31_252+32insC		-	Cluster_16_NRPS
Chr5	2614729	SNV	T	A	1	ISSFT-021, IF1SW-F4			-	Cluster_16_NRPS
Chr5	2615047	SNV	A	G	1	CEA10, ISSFT-021, IF1SW-F4	Afu5g10170:c.984T>C		No	Cluster_16_NRPS
Chr5	2617407	SNV	G	T	1	IF1SW-F4	Afu5g10180:c.478G>T	Afu5g10180:p.Val160Phe	Yes	Cluster_16_NRPS
Chr5	2618352	SNV	G	T	1	IF1SW-F4	Afu5g10180:c.1278G>T	Afu5g10180:p.Met426Ile	Yes	Cluster_16_NRPS
Chr5	2618651	SNV	A	G	1	ISSFT-021, IF1SW-F4	Afu5g10190:c.767T>C	Afu5g10190:p.Val256Ala	Yes	Cluster_16_NRPS
Chr5	2619454	SNV	G	A	1	ISSFT-021, IF1SW-F4	Afu5g10190:c.21C>T		No	Cluster_16_NRPS
Chr5	2619889	SNV	G	A	1	ISSFT-021, IF1SW-F4	Afu5g10190:c.-415C>T		-	Cluster_16_NRPS
Chr5	2620351	SNV	A	G	1	ISSFT-021, IF1SW-F4	Afu5g10200:c.1129T>C	Afu5g10200:p.Phe377Leu	Yes	Cluster_16_NRPS
Chr5	2620849	SNV	C	T	1	CEA10, ISSFT-021, IF1SW-F4	Afu5g10200:c.631G>A	Afu5g10200:p.Glu211Lys	Yes	Cluster_16_NRPS
Chr5	2622012	SNV	T	G	1	CEA10, ISSFT-021, IF1SW-F4	Afu5g10210:c.475A>C		No	Cluster_16_NRPS
Chr5	2622254	SNV	C	G	1	CEA10, ISSFT-021, IF1SW-F4	Afu5g10210:c.233G>C	Afu5g10210:p.Arg78Pro	Yes	Cluster_16_NRPS
Chr5	2622684	SNV	A	G	1	CEA10			-	Cluster_16_NRPS
Chr5	3302668	SNV	C	A	1	CEA10, ISSFT-021, IF1SW-F4			-	Cluster_17_NRPS
Chr5	3303104	SNV	T	A	1	CEA10, ISSFT-021, IF1SW-F4	Afu5g12700:c.300T>A	Afu5g12700:p.Tyr100*	Yes	Cluster_17_NRPS
Chr5	3303298	SNV	C	T	1	CEA10, ISSFT-021, IF1SW-F4	Afu5g12700:c.494C>T	Afu5g12700:p.Pro165Leu	Yes	Cluster_17_NRPS
Chr5	3303358..3303362	MNV	TAACT	CAACC	5	CEA10, ISSFT-021, IF1SW-F4			-	Cluster_17_NRPS
Chr5	3303708	SNV	A	C	1	CEA10, ISSFT-021, IF1SW-F4			-	Cluster_17_NRPS
Chr5	3303830..3303831	MNV	CC	AA	2	CEA10, ISSFT-021, IF1SW-F4			-	Cluster_17_NRPS
Chr5	3303892	SNV	A	G	1	CEA10, ISSFT-021, IF1SW-F4			-	Cluster_17_NRPS
Chr5	3303964	SNV	T	G	1	CEA10, ISSFT-021, IF1SW-F4			-	Cluster_17_NRPS
Chr5	3304170	SNV	G	A	1	CEA10, ISSFT-021, IF1SW-F4			-	Cluster_17_NRPS
Chr5	3304322	SNV	T	C	1	CEA10, ISSFT-021, IF1SW-F4			-	Cluster_17_NRPS
Chr5	3304513	SNV	C	T	1	CEA10, ISSFT-021, IF1SW-F4			-	Cluster_17_NRPS
Chr5	3304526	SNV	G	A	1	CEA10, ISSFT-021, IF1SW-F4			-	Cluster_17_NRPS
Chr5	3304539	SNV	G	A	1	CEA10, ISSFT-021, IF1SW-F4			-	Cluster_17_NRPS
Chr5	3304721	SNV	A	C	1	CEA10, ISSFT-021, IF1SW-F4			-	Cluster_17_NRPS
Chr5	3304756	SNV	A	C	1	CEA10, ISSFT-021, IF1SW-F4			-	Cluster_17_NRPS
Chr5	3304824	SNV	A	G	1	CEA10, ISSFT-021, IF1SW-F4			-	Cluster_17_NRPS
Chr5	3304946	SNV	G	C	1	CEA10, ISSFT-021, IF1SW-F4	Afu5g12710:c.99G>C		No	Cluster_17_NRPS
Chr5	3305104	SNV	C	T	1	CEA10, ISSFT-021, IF1SW-F4	Afu5g12710:c.257C>T	Afu5g12710:p.Ala86Val	Yes	Cluster_17_NRPS
Chr5	3305386	SNV	T	C	1	CEA10, ISSFT-021, IF1SW-F4	Afu5g12710:c.539T>C	Afu5g12710:p.Leu180Pro	Yes	Cluster_17_NRPS
Chr5	3305548	SNV	A	G	1	CEA10, ISSFT-021, IF1SW-F4	Afu5g12710:c.669-21A>G		-	Cluster_17_NRPS
Chr5	3305694	SNV	C	T	1	CEA10, ISSFT-021, IF1SW-F4	Afu5g12710:c.794C>T	Afu5g12710:p.Ser265Leu	Yes	Cluster_17_NRPS
Chr5	3305826	SNV	G	A	1	CEA10, ISSFT-021, IF1SW-F4	Afu5g12710:c.926G>A	Afu5g12710:p.Arg309Gln	Yes	Cluster_17_NRPS
Chr5	3306385	SNV	G	T	1	CEA10, ISSFT-021, IF1SW-F4	Afu5g12710:c.1437G>T		No	Cluster_17_NRPS
Chr5	3306500	SNV	C	T	1	CEA10, ISSFT-021, IF1SW-F4	Afu5g12710:c.1552C>T	Afu5g12710:p.Arg518Trp	Yes	Cluster_17_NRPS
Chr5	3307224^3307225	Insertion	-	T	1	CEA10, IF1SW-F4			-	Cluster_17_NRPS
Chr5	3307564	Deletion	T	-	1	CEA10, IF1SW-F4			-	Cluster_17_NRPS
Chr5	3307704	SNV	C	G	1	CEA10, ISSFT-021, IF1SW-F4			-	Cluster_17_NRPS

Chr5	3308688	SNV	A	G	1	CEA10, ISSFT-021, IF1SW-F4	Afu5g12720:c.3543T>C		No	Cluster_17_NRPS
Chr5	3308754	SNV	A	C	1	CEA10, ISSFT-021, IF1SW-F4	Afu5g12720:c.3477T>G		No	Cluster_17_NRPS
Chr5	3309384	SNV	T	C	1	CEA10, ISSFT-021, IF1SW-F4	Afu5g12720:c.2886+35A>G		-	Cluster_17_NRPS
Chr5	3309416	SNV	T	A	1	CEA10, ISSFT-021, IF1SW-F4	Afu5g12720:c.2886+3A>T		-	Cluster_17_NRPS
Chr5	3309634	SNV	G	A	1	CEA10, ISSFT-021, IF1SW-F4	Afu5g12720:c.2671C>T	Afu5g12720:p.Gln891*	Yes	Cluster_17_NRPS
Chr5	3309866	SNV	T	C	1	CEA10, ISSFT-021, IF1SW-F4	Afu5g12720:c.2494-55A>G		-	Cluster_17_NRPS
Chr5	3309878	SNV	C	T	1	CEA10, ISSFT-021, IF1SW-F4	Afu5g12720:c.2493+46G>A		-	Cluster_17_NRPS
Chr5	3309972..3309973	MNV	TA	CG	2	CEA10, ISSFT-021, IF1SW-F4	Afu5g12720:c.2444_2445delTAinsCG	Afu5g12720:p.Leu815Pro	Yes	Cluster_17_NRPS
Chr5	3310051	SNV	C	A	1	CEA10, ISSFT-021, IF1SW-F4	Afu5g12720:c.2416+44G>T		-	Cluster_17_NRPS
Chr5	3310120	SNV	A	G	1	CEA10, ISSFT-021, IF1SW-F4	Afu5g12720:c.2391T>C		No	Cluster_17_NRPS
Chr5	3310294	SNV	G	A	1	CEA10, ISSFT-021, IF1SW-F4	Afu5g12720:c.2217C>T		No	Cluster_17_NRPS
Chr5	3310978	SNV	T	C	1	CEA10, ISSFT-021, IF1SW-F4	Afu5g12720:c.1533A>G		No	Cluster_17_NRPS
Chr5	3311190	SNV	A	G	1	CEA10, ISSFT-021, IF1SW-F4	Afu5g12720:c.1383T>C		No	Cluster_17_NRPS
Chr5	3311307	SNV	A	G	1	CEA10, ISSFT-021, IF1SW-F4	Afu5g12720:c.1266T>C		No	Cluster_17_NRPS
Chr5	3311351	SNV	C	T	1	CEA10, ISSFT-021, IF1SW-F4	Afu5g12720:c.1264+13G>A		-	Cluster_17_NRPS
Chr5	3311967	SNV	G	A	1	CEA10, ISSFT-021, IF1SW-F4	Afu5g12720:c.718C>T	Afu5g12720:p.Pro240Ser	Yes	Cluster_17_NRPS
Chr5	3312016	SNV	G	A	1	CEA10, ISSFT-021, IF1SW-F4	Afu5g12720:c.701+6C>T		-	Cluster_17_NRPS
Chr5	3312216	SNV	A	G	1	CEA10, ISSFT-021, IF1SW-F4	Afu5g12720:c.507T>C		No	Cluster_17_NRPS
Chr5	3312271	SNV	C	G	1	CEA10, ISSFT-021, IF1SW-F4	Afu5g12720:c.452G>C	Afu5g12720:p.Arg151Pro	Yes	Cluster_17_NRPS
Chr5	3312277	SNV	A	T	1	IF1SW-F4	Afu5g12720:c.446T>A	Afu5g12720:p.Phe149Tyr	Yes	Cluster_17_NRPS
Chr5	3312399	SNV	G	A	1	CEA10, ISSFT-021, IF1SW-F4	Afu5g12720:c.324C>T		No	Cluster_17_NRPS
Chr5	3312425	SNV	T	C	1	CEA10, ISSFT-021, IF1SW-F4	Afu5g12720:c.298A>G	Afu5g12720:p.Ile100Val	Yes	Cluster_17_NRPS
Chr5	3312618	SNV	C	G	1	CEA10, ISSFT-021, IF1SW-F4	Afu5g12720:c.105G>C		No	Cluster_17_NRPS
Chr5	3312750	SNV	A	G	1	CEA10, ISSFT-021, IF1SW-F4	Afu5g12720:c.-20-8T>C		-	Cluster_17_NRPS
Chr5	3312778..3312788	Replacemen	GCAGATTCGAG	ACA	11	CEA10, ISSFT-021, IF1SW-F4	12720:c.-20-46_-20-36delCTCGAATCTGC		-	Cluster_17_NRPS
Chr5	3312911	SNV	C	T	1	CEA10, ISSFT-021, IF1SW-F4	Afu5g12720:c.-27G>A		-	Cluster_17_NRPS
Chr5	3312999	SNV	G	C	1	CEA10, ISSFT-021, IF1SW-F4	Afu5g12720:c.-115C>G		-	Cluster_17_NRPS
Chr5	3313146	SNV	A	G	1	CEA10, ISSFT-021, IF1SW-F4	Afu5g12720:c.-262T>C		-	Cluster_17_NRPS
Chr5	3313188	SNV	A	G	1	CEA10, ISSFT-021, IF1SW-F4	Afu5g12720:c.-304T>C		-	Cluster_17_NRPS
Chr5	3313218	SNV	A	G	1	CEA10, ISSFT-021, IF1SW-F4	Afu5g12720:c.-334T>C		-	Cluster_17_NRPS
Chr5	3313458	SNV	C	A	1	CEA10, ISSFT-021, IF1SW-F4			-	Cluster_17_NRPS
Chr5	3313502	SNV	C	A	1	CEA10, ISSFT-021, IF1SW-F4			-	Cluster_17_NRPS
Chr5	3313706	SNV	C	T	1	CEA10, ISSFT-021, IF1SW-F4			-	Cluster_17_NRPS
Chr5	3313775	SNV	C	T	1	CEA10, ISSFT-021, IF1SW-F4			-	Cluster_17_NRPS
Chr5	3313854	SNV	G	A	1	CEA10, ISSFT-021, IF1SW-F4			-	Cluster_17_NRPS
Chr5	3313921	SNV	G	A	1	CEA10, ISSFT-021, IF1SW-F4			-	Cluster_17_NRPS
Chr5	3314044	SNV	A	C	1	CEA10, ISSFT-021, IF1SW-F4			-	Cluster_17_NRPS
Chr5	3314218	SNV	A	G	1	CEA10, ISSFT-021, IF1SW-F4			-	Cluster_17_NRPS
Chr5	3314449	SNV	G	T	1	CEA10, ISSFT-021, IF1SW-F4			-	Cluster_17_NRPS
Chr5	3314942	SNV	A	G	1	CEA10, ISSFT-021, IF1SW-F4	Afu5g12730:c.406A>G	Afu5g12730:p.Met136Val	Yes	Cluster_17_NRPS
Chr5	3315158	SNV	A	T	1	CEA10, ISSFT-021, IF1SW-F4	Afu5g12730:c.622A>T	Afu5g12730:p.Lys208*	Yes	Cluster_17_NRPS
Chr5	3315292	SNV	T	C	1	CEA10, ISSFT-021, IF1SW-F4	Afu5g12730:c.756T>C		No	Cluster_17_NRPS
Chr5	3315487	SNV	G	A	1	CEA10, ISSFT-021, IF1SW-F4	Afu5g12730:c.951G>A		No	Cluster_17_NRPS
Chr5	3318191	SNV	C	T	1	CEA10	Afu5g12730:c.3655C>T	Afu5g12730:p.Arg1219*	Yes	Cluster_17_NRPS
Chr5	3318470	SNV	C	T	1	CEA10, ISSFT-021, IF1SW-F4	Afu5g12730:c.3934C>T	Afu5g12730:p.Gln1312*	Yes	Cluster_17_NRPS
Chr5	3318580	SNV	C	T	1	CEA10, ISSFT-021, IF1SW-F4	Afu5g12730:c.4044C>T		No	Cluster_17_NRPS
Chr5	3319154	SNV	C	T	1	CEA10, ISSFT-021, IF1SW-F4	Afu5g12730:c.4618C>T	Afu5g12730:p.Pro1540Ser	Yes	Cluster_17_NRPS
Chr5	3320078..3320079	Deletion	TT	-	2	CEA10, ISSFT-021, IF1SW-F4	Afu5g12730:c.5542_5543delTT	Afu5g12730:p.Leu1848fs	Yes	Cluster_17_NRPS
Chr5	3321933	SNV	C	A	1	CEA10, ISSFT-021, IF1SW-F4	Afu5g12730:c.7397C>A	Afu5g12730:p.Thr2466Asn	Yes	Cluster_17_NRPS
Chr5	3322661	SNV	T	C	1	CEA10, ISSFT-021, IF1SW-F4	Afu5g12730:c.8125T>C		No	Cluster_17_NRPS
Chr5	3323193	SNV	C	T	1	CEA10, ISSFT-021, IF1SW-F4	Afu5g12730:c.8657C>T	Afu5g12730:p.Thr2886Ile	Yes	Cluster_17_NRPS
Chr5	3323868	SNV	G	T	1	CEA10, ISSFT-021, IF1SW-F4	Afu5g12730:c.9332G>T	Afu5g12730:p.Arg3111Leu	Yes	Cluster_17_NRPS
Chr5	3323962	SNV	A	G	1	CEA10, ISSFT-021, IF1SW-F4	Afu5g12730:c.9426A>G		No	Cluster_17_NRPS
Chr5	3324005	SNV	G	C	1	CEA10, ISSFT-021, IF1SW-F4	Afu5g12730:c.9469G>C	Afu5g12730:p.Glu3157Gln	Yes	Cluster_17_NRPS
Chr5	3325018	SNV	C	T	1	CEA10, ISSFT-021, IF1SW-F4	Afu5g12730:c.10482C>T		No	Cluster_17_NRPS
Chr5	3325230	SNV	A	G	1	CEA10, ISSFT-021, IF1SW-F4	Afu5g12730:c.10694A>G	Afu5g12730:p.Glu3565Gly	Yes	Cluster_17_NRPS
Chr5	3325243	SNV	C	T	1	CEA10, ISSFT-021, IF1SW-F4	Afu5g12730:c.10707C>T		No	Cluster_17_NRPS
Chr5	3325858	SNV	A	G	1	CEA10, ISSFT-021, IF1SW-F4	Afu5g12730:c.11322A>G		No	Cluster_17_NRPS
Chr5	3325867	SNV	C	T	1	CEA10, ISSFT-021, IF1SW-F4	Afu5g12730:c.11331C>T		No	Cluster_17_NRPS

Chr5	3326046	SNV	G	A	1	CEA10, ISSFT-021, IF1SW-F4	Afu5g12730:c.11510G>A	Afu5g12730:p.Gly3837Asp	Yes	Cluster_17_NRPS
Chr5	3326649	SNV	A	G	1	CEA10, ISSFT-021, IF1SW-F4	Afu5g12730:c.12113A>G	Afu5g12730:p.Asp4038Gly	Yes	Cluster_17_NRPS
Chr5	3326900	SNV	C	A	1	CEA10, ISSFT-021, IF1SW-F4	Afu5g12730:c.12364C>A	Afu5g12730:p.Gln4122Lys	Yes	Cluster_17_NRPS
Chr5	3327370	SNV	A	G	1	CEA10, ISSFT-021, IF1SW-F4	Afu5g12730:c.12834A>G		No	Cluster_17_NRPS
Chr5	3327493	SNV	T	C	1	CEA10, ISSFT-021, IF1SW-F4	Afu5g12730:c.12957T>C		No	Cluster_17_NRPS
Chr5	3327767	SNV	G	T	1	CEA10, ISSFT-021, IF1SW-F4	Afu5g12730:c.13231G>T	Afu5g12730:p.Asp4411Tyr	Yes	Cluster_17_NRPS
Chr5	3327841	SNV	T	C	1	CEA10, ISSFT-021, IF1SW-F4	Afu5g12730:c.13305T>C		No	Cluster_17_NRPS
Chr5	3327912	SNV	A	G	1	CEA10, ISSFT-021, IF1SW-F4	Afu5g12730:c.13376A>G	Afu5g12730:p.Gln4459Arg	Yes	Cluster_17_NRPS
Chr5	3328522	SNV	C	T	1	CEA10, ISSFT-021, IF1SW-F4	Afu5g12730:c.13986C>T		No	Cluster_17_NRPS
Chr5	3328888	SNV	C	A	1	CEA10, ISSFT-021, IF1SW-F4	Afu5g12730:c.14352C>A		No	Cluster_17_NRPS
Chr5	3329140	SNV	G	C	1	CEA10, ISSFT-021, IF1SW-F4	Afu5g12730:c.14604G>C		No	Cluster_17_NRPS
Chr5	3329692	SNV	C	A	1	CEA10, ISSFT-021, IF1SW-F4	Afu5g12730:c.15156C>A		No	Cluster_17_NRPS
Chr5	3329754	SNV	A	C	1	CEA10, ISSFT-021, IF1SW-F4	Afu5g12730:c.15218A>C	Afu5g12730:p.His5073Pro	Yes	Cluster_17_NRPS
Chr5	3329808	SNV	C	T	1	CEA10, ISSFT-021, IF1SW-F4	Afu5g12730:c.15272C>T	Afu5g12730:p.Thr5091Met	Yes	Cluster_17_NRPS
Chr5	3329824	SNV	T	C	1	CEA10, ISSFT-021, IF1SW-F4	Afu5g12730:c.15288T>C		No	Cluster_17_NRPS
Chr5	3330409	SNV	G	A	1	CEA10, ISSFT-021, IF1SW-F4	Afu5g12730:c.15873G>A		No	Cluster_17_NRPS
Chr5	3330438	SNV	G	A	1	CEA10, ISSFT-021, IF1SW-F4	Afu5g12730:c.15902G>A	Afu5g12730:p.Gly5301Glu	Yes	Cluster_17_NRPS
Chr5	3330869	SNV	T	C	1	CEA10, ISSFT-021, IF1SW-F4	Afu5g12730:c.16333T>C	Afu5g12730:p.Phe5445Leu	Yes	Cluster_17_NRPS
Chr5	3330929	SNV	G	A	1	CEA10, ISSFT-021, IF1SW-F4	Afu5g12730:c.16393G>A	Afu5g12730:p.Val5465Met	Yes	Cluster_17_NRPS
Chr5	3331152	SNV	C	G	1	CEA10, ISSFT-021, IF1SW-F4	Afu5g12730:c.16616C>G	Afu5g12730:p.Ala5539Gly	Yes	Cluster_17_NRPS
Chr5	3331200	SNV	A	G	1	CEA10, ISSFT-021, IF1SW-F4	Afu5g12730:c.16664A>G	Afu5g12730:p.Gln5555Arg	Yes	Cluster_17_NRPS
Chr5	3331391	SNV	A	G	1	CEA10, ISSFT-021, IF1SW-F4	Afu5g12730:c.16855A>G	Afu5g12730:p.Ile5619Val	Yes	Cluster_17_NRPS
Chr5	3331410	SNV	A	G	1	CEA10, ISSFT-021, IF1SW-F4	Afu5g12730:c.16874A>G	Afu5g12730:p.Gln5625Arg	Yes	Cluster_17_NRPS
Chr5	3331533	SNV	A	G	1	CEA10, ISSFT-021, IF1SW-F4	Afu5g12730:c.16997A>G	Afu5g12730:p.Tyr5666Cys	Yes	Cluster_17_NRPS
Chr5	3331567	SNV	G	A	1	CEA10	Afu5g12730:c.17031G>A		No	Cluster_17_NRPS
Chr5	3331750	SNV	T	C	1	CEA10, ISSFT-021, IF1SW-F4	Afu5g12730:c.17214T>C		No	Cluster_17_NRPS
Chr5	3332240	SNV	A	G	1	CEA10, ISSFT-021, IF1SW-F4	Afu5g12730:c.17704A>G	Afu5g12730:p.Met5902Val	Yes	Cluster_17_NRPS
Chr5	3332594^3332595	Insertion	-	G	1	ISSFT-021	Afu5g12730:c.18058_18059insG	Afu5g12730:p.Ala6020fs	Yes	Cluster_17_NRPS
Chr5	3332765	SNV	G	T	1	CEA10, ISSFT-021, IF1SW-F4	Afu5g12730:c.18229G>T	Afu5g12730:p.Glu6077*	Yes	Cluster_17_NRPS
Chr5	3333559	SNV	T	A	1	CEA10, ISSFT-021, IF1SW-F4	Afu5g12730:c.19023T>A		No	Cluster_17_NRPS
Chr5	3333782	SNV	G	A	1	CEA10, ISSFT-021, IF1SW-F4	Afu5g12730:c.19246G>A	Afu5g12730:p.Ala6416Thr	Yes	Cluster_17_NRPS
Chr5	3333967	SNV	A	G	1	CEA10, ISSFT-021, IF1SW-F4	Afu5g12730:c.19431A>G		No	Cluster_17_NRPS
Chr5	3333983	SNV	A	G	1	CEA10, ISSFT-021, IF1SW-F4	Afu5g12730:c.19447A>G	Afu5g12730:p.Ile6483Val	Yes	Cluster_17_NRPS
Chr5	3334093	SNV	C	A	1	ISSFT-021	Afu5g12730:c.19557C>A		No	Cluster_17_NRPS
Chr5	3334241	SNV	T	A	1	CEA10, ISSFT-021, IF1SW-F4	Afu5g12730:c.19705T>A	Afu5g12730:p.Ser6569Thr	Yes	Cluster_17_NRPS
Chr5	3334456	SNV	G	A	1	CEA10, ISSFT-021, IF1SW-F4	Afu5g12730:c.19920G>A		No	Cluster_17_NRPS
Chr5	3334830..3334831	MNV	GA	AG	2	CEA10, ISSFT-021, IF1SW-F4	Afu5g12730:c.20294_20295delGAinsAG	Afu5g12730:p.Arg6765Gln	Yes	Cluster_17_NRPS
Chr5	3335551	SNV	T	C	1	CEA10, ISSFT-021, IF1SW-F4	Afu5g12730:c.21015T>C		No	Cluster_17_NRPS
Chr5	3335559	SNV	A	G	1	CEA10, ISSFT-021, IF1SW-F4	Afu5g12730:c.21023A>G	Afu5g12730:p.Asp7008Gly	Yes	Cluster_17_NRPS
Chr5	3336004	SNV	G	A	1	CEA10, ISSFT-021, IF1SW-F4	Afu5g12730:c.21468G>A		No	Cluster_17_NRPS
Chr5	3336140	SNV	G	T	1	CEA10, ISSFT-021, IF1SW-F4	Afu5g12730:c.21604G>T	Afu5g12730:p.Ala7202Ser	Yes	Cluster_17_NRPS
Chr5	3336500	SNV	C	T	1	CEA10, ISSFT-021, IF1SW-F4	Afu5g12730:c.21964C>T	Afu5g12730:p.Leu7322Phe	Yes	Cluster_17_NRPS
Chr5	3336987	SNV	C	T	1	CEA10, ISSFT-021, IF1SW-F4	Afu5g12730:c.22451C>T	Afu5g12730:p.Pro7484Leu	Yes	Cluster_17_NRPS
Chr5	3337201	SNV	C	T	1	CEA10, ISSFT-021, IF1SW-F4	Afu5g12730:c.22665C>T		No	Cluster_17_NRPS
Chr5	3337615	SNV	T	C	1	CEA10, ISSFT-021, IF1SW-F4	Afu5g12730:c.23079T>C		No	Cluster_17_NRPS
Chr5	3337895	SNV	C	T	1	CEA10, ISSFT-021, IF1SW-F4	Afu5g12730:c.23359C>T	Afu5g12730:p.Gln7787*	Yes	Cluster_17_NRPS
Chr5	3338443	SNV	G	A	1	CEA10, ISSFT-021, IF1SW-F4	Afu5g12730:c.23907G>A		No	Cluster_17_NRPS
Chr5	3338950	SNV	C	T	1	CEA10, ISSFT-021, IF1SW-F4	Afu5g12730:c.24414C>T		No	Cluster_17_NRPS
Chr5	3339054	SNV	A	G	1	CEA10, ISSFT-021, IF1SW-F4	Afu5g12730:c.24518A>G	Afu5g12730:p.Lys8173Arg	Yes	Cluster_17_NRPS
Chr5	3339157	SNV	C	T	1	CEA10, ISSFT-021, IF1SW-F4	Afu5g12730:c.24621C>T		No	Cluster_17_NRPS
Chr5	3339379	SNV	A	C	1	CEA10, ISSFT-021, IF1SW-F4	Afu5g12730:c.24843A>C		No	Cluster_17_NRPS
Chr5	3339558	SNV	T	G	1	CEA10, ISSFT-021, IF1SW-F4	Afu5g12730:c.25022T>G	Afu5g12730:p.Ile8341Ser	Yes	Cluster_17_NRPS
Chr5	3339680	SNV	C	T	1	CEA10, ISSFT-021, IF1SW-F4	Afu5g12730:c.25144C>T	Afu5g12730:p.Arg8382Cys	Yes	Cluster_17_NRPS
Chr5	3340734	SNV	T	A	1	CEA10, ISSFT-021, IF1SW-F4	Afu5g12740:c.-503T>A		-	Cluster_17_NRPS
Chr5	3341230	SNV	C	G	1	CEA10, ISSFT-021	Afu5g12740:c.-7C>G		-	Cluster_17_NRPS
Chr5	3341315..3341317	Deletion	TTC	-	3	ISSFT-021	Afu5g12740:c.79_81delTTC	Afu5g12740:p.Phe27del	Yes	Cluster_17_NRPS
Chr5	3341895	SNV	T	C	1	CEA10, ISSFT-021, IF1SW-F4	Afu5g12740:c.531T>C		No	Cluster_17_NRPS
Chr5	3342123	SNV	C	T	1	CEA10, ISSFT-021, IF1SW-F4	Afu5g12740:c.759C>T		No	Cluster_17_NRPS
Chr5	3342140	SNV	A	G	1	CEA10, ISSFT-021, IF1SW-F4	Afu5g12740:c.776A>G	Afu5g12740:p.Asn259Ser	Yes	Cluster_17_NRPS

Chr5	3342261..3342262	MNV	AA	CC	2	CEA10, ISSFT-021, IF1SW-F4	Afu5g12740:c.897_898delAAinsCC		No	Cluster_17_NRPS
Chr5	3342455	SNV	A	C	1	CEA10, ISSFT-021, IF1SW-F4	Afu5g12740:c.1091A>C	Afu5g12740:p.Glu364Ala	Yes	Cluster_17_NRPS
Chr5	3342543	SNV	G	A	1	CEA10, ISSFT-021, IF1SW-F4	Afu5g12740:c.1179G>A		No	Cluster_17_NRPS
Chr5	3342651	SNV	G	A	1	CEA10, ISSFT-021	Afu5g12740:c.1287G>A		No	Cluster_17_NRPS
Chr5	3342746	SNV	G	C	1	CEA10, ISSFT-021, IF1SW-F4	Afu5g12740:c.1382G>C	Afu5g12740:p.Arg461Pro	Yes	Cluster_17_NRPS
Chr5	3342879	SNV	A	G	1	CEA10, ISSFT-021, IF1SW-F4	Afu5g12740:c.*87A>G		-	Cluster_17_NRPS
Chr5	3342913	SNV	T	C	1	CEA10, ISSFT-021, IF1SW-F4	Afu5g12740:c.*121T>C		-	Cluster_17_NRPS
Chr5	3342960	SNV	A	G	1	CEA10, ISSFT-021, IF1SW-F4	Afu5g12740:c.*168A>G		-	Cluster_17_NRPS
Chr5	3343048	SNV	A	G	1	CEA10, ISSFT-021, IF1SW-F4	Afu5g12740:c.*256A>G		-	Cluster_17_NRPS
Chr5	3343268	SNV	T	C	1	CEA10, ISSFT-021, IF1SW-F4	Afu5g12740:c.*476T>C		-	Cluster_17_NRPS
Chr5	3343349	SNV	T	C	1	ISSFT-021	Afu5g12740:c.*557T>C		-	Cluster_17_NRPS
Chr5	3343366	SNV	C	T	1	CEA10, ISSFT-021, IF1SW-F4	Afu5g12740:c.*574C>T		-	Cluster_17_NRPS
Chr5	3343879	Deletion	T	-	1	ISSFT-021			-	Cluster_17_NRPS
Chr5	3346091	SNV	A	G	1	IF1SW-F4			-	Cluster_17_NRPS
Chr5	3346149	SNV	G	A	1	CEA10, ISSFT-021, IF1SW-F4			-	Cluster_17_NRPS
Chr5	3346728	SNV	G	A	1	CEA10, ISSFT-021, IF1SW-F4			-	Cluster_17_NRPS
Chr5	3347144	SNV	C	A	1	IF1SW-F4	Afu5g12760:c.1046G>T	Afu5g12760:p.Gly349Val	Yes	Cluster_17_NRPS
Chr5	3348662	SNV	A	G	1	IF1SW-F4			-	Cluster_17_NRPS
Chr5	3350862	SNV	T	C	1	IF1SW-F4	Afu5g12780:c.-1213T>C		-	Cluster_17_NRPS
Chr5	3351511..3351512	Deletion	TA	-	2	CEA10, ISSFT-021	Afu5g12780:c.-564_-563delTA		-	Cluster_17_NRPS
Chr5	3351943	SNV	C	G	1	CEA10, ISSFT-021	Afu5g12780:c.-132C>G		-	Cluster_17_NRPS
Chr5	3352772	SNV	T	C	1	CEA10, ISSFT-021, IF1SW-F4	Afu5g12780:c.642T>C		No	Cluster_17_NRPS
Chr5	3353309	SNV	T	A	1	CEA10			-	Cluster_17_NRPS
Chr5	3354748	SNV	T	G	1	CEA10, ISSFT-021	Afu5g12790:c.406-13A>C		-	Cluster_17_NRPS
Chr5	3354750	SNV	T	C	1	IF1SW-F4	Afu5g12790:c.405+13A>G		-	Cluster_17_NRPS
Chr6	735303	SNV	C	T	1	CEA10, ISSFT-021, IF1SW-F4	Afu6g03430:c.317C>T	Afu6g03430:p.Pro106Leu	Yes	Cluster_18_fumisoquin
Chr6	735704	SNV	T	G	1	ISSFT-021, IF1SW-F4	Afu6g03430:c.718T>G	Afu6g03430:p.Tyr240Asp	Yes	Cluster_18_fumisoquin
Chr6	736407	SNV	A	G	1	CEA10, ISSFT-021, IF1SW-F4	Afu6g03430:c.1421A>G	Afu6g03430:p.Tyr474Cys	Yes	Cluster_18_fumisoquin
Chr6	736607^736608	Insertion	-	GA	2	ISSFT-021, IF1SW-F4			-	Cluster_18_fumisoquin
Chr6	736635	SNV	G	T	1	CEA10, ISSFT-021, IF1SW-F4			-	Cluster_18_fumisoquin
Chr6	737060	SNV	G	A	1	ISSFT-021	Afu6g03440:c.1215C>T		No	Cluster_18_fumisoquin
Chr6	737743	SNV	C	T	1	CEA10, ISSFT-021, IF1SW-F4	Afu6g03440:c.532G>A	Afu6g03440:p.Val178Ile	Yes	Cluster_18_fumisoquin
Chr6	737782	SNV	C	G	1	ISSFT-021	Afu6g03440:c.493G>C	Afu6g03440:p.Val165Leu	Yes	Cluster_18_fumisoquin
Chr6	738438	SNV	C	T	1	IF1SW-F4			-	Cluster_18_fumisoquin
Chr6	738523	SNV	C	G	1	IF1SW-F4			-	Cluster_18_fumisoquin
Chr6	738545	SNV	C	T	1	CEA10			-	Cluster_18_fumisoquin
Chr6	738906	SNV	C	T	1	ISSFT-021	Afu6g03450:c.-13C>T		-	Cluster_18_fumisoquin
Chr6	738943	SNV	C	T	1	CEA10, ISSFT-021, IF1SW-F4	Afu6g03450:c.25C>T	Afu6g03450:p.Arg9Cys	Yes	Cluster_18_fumisoquin
Chr6	739224	SNV	C	T	1	IF1SW-F4	Afu6g03450:c.306C>T		No	Cluster_18_fumisoquin
Chr6	740047	SNV	T	C	1	ISSFT-021, IF1SW-F4	Afu6g03450:c.1055T>C	Afu6g03450:p.Leu352Pro	Yes	Cluster_18_fumisoquin
Chr6	740452	SNV	A	C	1	ISSFT-021, IF1SW-F4			-	Cluster_18_fumisoquin
Chr6	740554	SNV	G	A	1	CEA10	Afu6g03460:c.-617G>A		-	Cluster_18_fumisoquin
Chr6	740717	Deletion	T	-	1	ISSFT-021	Afu6g03460:c.-454delT		-	Cluster_18_fumisoquin
Chr6	741060	SNV	T	C	1	CEA10, ISSFT-021, IF1SW-F4	Afu6g03460:c.-111T>C		-	Cluster_18_fumisoquin
Chr6	742050	SNV	G	A	1	CEA10, ISSFT-021, IF1SW-F4	Afu6g03460:c.880G>A	Afu6g03460:p.Val294Ile	Yes	Cluster_18_fumisoquin
Chr6	742190	SNV	C	T	1	ISSFT-021, IF1SW-F4	Afu6g03460:c.1020C>T		No	Cluster_18_fumisoquin
Chr6	742435	SNV	G	C	1	CEA10, ISSFT-021, IF1SW-F4	Afu6g03460:c.1265G>C	Afu6g03460:p.Arg422Pro	Yes	Cluster_18_fumisoquin
Chr6	743418	SNV	G	T	1	ISSFT-021, IF1SW-F4	Afu6g03470:c.-149G>T		-	Cluster_18_fumisoquin
Chr6	744213	SNV	T	C	1	CEA10	Afu6g03470:c.581T>C	Afu6g03470:p.Phe194Ser	Yes	Cluster_18_fumisoquin
Chr6	744904	SNV	G	A	1	CEA10, ISSFT-021, IF1SW-F4	Afu6g03470:c.1272G>A		No	Cluster_18_fumisoquin
Chr6	745673	SNV	G	A	1	CEA10	Afu6g03470:c.1991G>A	Afu6g03470:p.Arg664Gln	Yes	Cluster_18_fumisoquin
Chr6	747543	SNV	A	G	1	IF1SW-F4	Afu6g03470:c.3730A>G	Afu6g03470:p.Thr1244Ala	Yes	Cluster_18_fumisoquin
Chr6	748197..748202	Deletion	CCCCCC	-	6	ISSFT-021			-	Cluster_18_fumisoquin
Chr6	749030	SNV	T	G	1	IF1SW-F4	Afu6g03480:c.636T>G	Afu6g03480:p.Asn212Lys	Yes	Cluster_18_fumisoquin
Chr6	749303	SNV	C	T	1	ISSFT-021	Afu6g03480:c.909C>T		No	Cluster_18_fumisoquin
Chr6	750005	SNV	G	A	1	ISSFT-021	Afu6g03480:c.1611G>A		No	Cluster_18_fumisoquin
Chr6	750221	SNV	T	A	1	CEA10, ISSFT-021	Afu6g03480:c.1827T>A		No	Cluster_18_fumisoquin
Chr6	750467	SNV	T	G	1	CEA10, ISSFT-021, IF1SW-F4	Afu6g03480:c.2073T>G		No	Cluster_18_fumisoquin
Chr6	750593	SNV	C	T	1	CEA10, ISSFT-021, IF1SW-F4	Afu6g03480:c.2199C>T		No	Cluster_18_fumisoquin

Chr6	751343	SNV	G	A	1	IF1SW-F4	Afu6g03480:c.2949G>A		No	Cluster_18_fumisoquin
Chr6	752162	SNV	G	T	1	ISSFT-021, IF1SW-F4	Afu6g03480:c.3768G>T		No	Cluster_18_fumisoquin
Chr6	752977	SNV	T	C	1	CEA10, ISSFT-021, IF1SW-F4	Afu6g03480:c.4494T>C		No	Cluster_18_fumisoquin
Chr6	753580	SNV	A	C	1	CEA10, ISSFT-021, IF1SW-F4	Afu6g03480:c.*69A>C		-	Cluster_18_fumisoquin
Chr6	754123	SNV	T	G	1	ISSFT-021, IF1SW-F4			-	Cluster_18_fumisoquin
Chr6	754651	SNV	T	C	1	CEA10, ISSFT-021, IF1SW-F4	Afu6g03490:c.338-3T>C		-	Cluster_18_fumisoquin
Chr6	756244	SNV	C	G	1	CEA10, ISSFT-021, IF1SW-F4	Afu6g03490:c.*58C>G		-	Cluster_18_fumisoquin
Chr6	756264	SNV	A	C	1	ISSFT-021, IF1SW-F4	Afu6g03490:c.*78A>C		-	Cluster_18_fumisoquin
Chr6	756327	SNV	A	G	1	ISSFT-021, IF1SW-F4	Afu6g03490:c.*141A>G		-	Cluster_18_fumisoquin
Chr6	2334884	SNV	G	A	1	CEA10	Afu6g09580:c.186G>A		No	Cluster_19_NRPS
Chr6	2335507	SNV	T	C	1	CEA10, ISSFT-021, IF1SW-F4	Afu6g09580:c.809T>C	Afu6g09580:p.Leu270Ser	Yes	Cluster_19_NRPS
Chr6	2335799	SNV	G	T	1	ISSFT-021	Afu6g09580:c.1101G>T		No	Cluster_19_NRPS
Chr6	2336596	SNV	G	C	1	CEA10, ISSFT-021, IF1SW-F4	Afu6g09590:c.179G>C	Afu6g09590:p.Arg60Pro	Yes	Cluster_19_NRPS
Chr6	2337242	SNV	T	C	1	CEA10, ISSFT-021, IF1SW-F4	Afu6g09590:c.825T>C		No	Cluster_19_NRPS
Chr6	2337484..2337500	Deletion	GTTGCCGAGTC	-	17	CEA10, ISSFT-021			-	Cluster_19_NRPS
Chr6	2338307	SNV	C	T	1	CEA10	Afu6g09600:c.474G>A		No	Cluster_19_NRPS
Chr6	2338340	SNV	A	G	1	CEA10	Afu6g09600:c.441T>C		No	Cluster_19_NRPS
Chr6	2338923	SNV	A	G	1	CEA10			-	Cluster_19_NRPS
Chr6	2338941	SNV	G	A	1	CEA10, ISSFT-021, IF1SW-F4			-	Cluster_19_NRPS
Chr6	2339109	SNV	A	G	1	CEA10, ISSFT-021, IF1SW-F4			-	Cluster_19_NRPS
Chr6	2339250	SNV	T	G	1	CEA10			-	Cluster_19_NRPS
Chr6	2339315	SNV	T	G	1	CEA10			-	Cluster_19_NRPS
Chr6	2339491	SNV	C	T	1	ISSFT-021			-	Cluster_19_NRPS
Chr6	2339518	SNV	T	C	1	CEA10			-	Cluster_19_NRPS
Chr6	2339749	SNV	A	G	1	CEA10	Afu6g09610:c.3197T>C	Afu6g09610:p.Phe1066Ser	Yes	Cluster_19_NRPS
Chr6	2340761	SNV	G	C	1	IF1SW-F4	Afu6g09610:c.2235-50C>G		-	Cluster_19_NRPS
Chr6	2341177	SNV	T	C	1	CEA10, ISSFT-021, IF1SW-F4	Afu6g09610:c.1927A>G	Afu6g09610:p.Ile643Val	Yes	Cluster_19_NRPS
Chr6	2341225	SNV	C	A	1	CEA10, ISSFT-021, IF1SW-F4	Afu6g09610:c.1879G>T	Afu6g09610:p.Ala627Ser	Yes	Cluster_19_NRPS
Chr6	2341307..2341336	Deletion	GCCCTTGCAG	-	30	ISSFT-021	1768_1797delTGTGTCTGCTCTGCAAGGG	Afu6g09610:p.Cys590_Pro599del	Yes	Cluster_19_NRPS
Chr6	2341340	SNV	G	A	1	ISSFT-021	Afu6g09610:c.1764C>T		No	Cluster_19_NRPS
Chr6	2341388	SNV	C	T	1	ISSFT-021, IF1SW-F4	Afu6g09610:c.1716G>A		No	Cluster_19_NRPS
Chr6	2341571	Deletion	A	-	1	ISSFT-021, IF1SW-F4	Afu6g09610:c.1533delT	Afu6g09610:p.Tyr512fs	Yes	Cluster_19_NRPS
Chr6	2342880	SNV	G	T	1	CEA10, ISSFT-021, IF1SW-F4	Afu6g09610:c.255C>A		No	Cluster_19_NRPS
Chr6	2344088	Deletion	T	-	1	CEA10	Afu6g09620:c.604delT	Afu6g09620:p.Arg203fs	Yes	Cluster_19_NRPS
Chr6	2346528	SNV	G	A	1	IF1SW-F4			-	Cluster_20_gliotoxin
Chr6	2346935	SNV	G	T	1	CEA10, ISSFT-021, IF1SW-F4	Afu6g09630:c.-51G>T		-	Cluster_20_gliotoxin
Chr6	2347397	SNV	A	G	1	CEA10, ISSFT-021	Afu6g09630:c.412A>G	Afu6g09630:p.Thr138Ala	Yes	Cluster_20_gliotoxin
Chr6	2348850	SNV	G	A	1	CEA10, ISSFT-021, IF1SW-F4			-	Cluster_20_gliotoxin
Chr6	2350285	SNV	G	C	1	CEA10, ISSFT-021, IF1SW-F4	Afu6g09640:c.318C>G		No	Cluster_20_gliotoxin
Chr6	2350428	SNV	T	C	1	CEA10, ISSFT-021, IF1SW-F4	Afu6g09640:c.175A>G	Afu6g09640:p.Thr59Ala	Yes	Cluster_20_gliotoxin
Chr6	2351243	SNV	C	T	1	CEA10, ISSFT-021, IF1SW-F4	Afu6g09650:c.246C>T		No	Cluster_20_gliotoxin
Chr6	2351547	SNV	A	G	1	CEA10, IF1SW-F4	Afu6g09650:c.550A>G	Afu6g09650:p.Lys184Glu	Yes	Cluster_20_gliotoxin
Chr6	2352096	SNV	G	A	1	CEA10	Afu6g09650:c.998G>A	Afu6g09650:p.Gly333Glu	Yes	Cluster_20_gliotoxin
Chr6	2352508	SNV	G	A	1	ISSFT-021			-	Cluster_20_gliotoxin
Chr6	2352825	SNV	T	C	1	CEA10, ISSFT-021, IF1SW-F4	Afu6g09660:c.6203A>G	Afu6g09660:p.Gln2068Arg	Yes	Cluster_20_gliotoxin
Chr6	2353201	SNV	G	A	1	CEA10	Afu6g09660:c.5827C>T	Afu6g09660:p.Pro1943Ser	Yes	Cluster_20_gliotoxin
Chr6	2353370	SNV	G	T	1	ISSFT-021	Afu6g09660:c.5658C>A	Afu6g09660:p.His1886Gln	Yes	Cluster_20_gliotoxin
Chr6	2353555	SNV	C	T	1	CEA10, ISSFT-021, IF1SW-F4	Afu6g09660:c.5473G>A	Afu6g09660:p.Ala1825Thr	Yes	Cluster_20_gliotoxin
Chr6	2356441	SNV	G	A	1	CEA10, ISSFT-021, IF1SW-F4	Afu6g09660:c.2637C>T		No	Cluster_20_gliotoxin
Chr6	2356498	SNV	T	G	1	CEA10, ISSFT-021, IF1SW-F4	Afu6g09660:c.2580A>C		No	Cluster_20_gliotoxin
Chr6	2356570	SNV	C	T	1	ISSFT-021	Afu6g09660:c.2508G>A		No	Cluster_20_gliotoxin
Chr6	2356866	SNV	T	A	1	CEA10, ISSFT-021, IF1SW-F4	Afu6g09660:c.2212A>T	Afu6g09660:p.Met738Leu	Yes	Cluster_20_gliotoxin
Chr6	2357092	SNV	C	T	1	CEA10, ISSFT-021, IF1SW-F4	Afu6g09660:c.1986G>A		No	Cluster_20_gliotoxin
Chr6	2357213	SNV	T	G	1	CEA10, ISSFT-021, IF1SW-F4	Afu6g09660:c.1865A>C	Afu6g09660:p.His622Pro	Yes	Cluster_20_gliotoxin
Chr6	2357599	SNV	C	T	1	CEA10, ISSFT-021, IF1SW-F4	Afu6g09660:c.1479G>A		No	Cluster_20_gliotoxin
Chr6	2357998	SNV	T	G	1	ISSFT-021, IF1SW-F4	Afu6g09660:c.1080A>C		No	Cluster_20_gliotoxin
Chr6	2358334	SNV	T	G	1	ISSFT-021, IF1SW-F4	Afu6g09660:c.744A>C		No	Cluster_20_gliotoxin
Chr6	2359131	SNV	G	A	1	ISSFT-021			-	Cluster_20_gliotoxin
Chr6	2359192	SNV	A	G	1	ISSFT-021			-	Cluster_20_gliotoxin

Chr6	2359549	SNV	A	G	1	CEA10, ISSFT-021, IF1SW-F4	Afu6g09670:c.21A>G	Afu6g09670:p.Ile7Met	Yes	Cluster_20_gliotoxin
Chr6	2359624	SNV	C	T	1	CEA10, ISSFT-021, IF1SW-F4	Afu6g09670:c.96C>T		No	Cluster_20_gliotoxin
Chr6	2359637	SNV	T	C	1	ISSFT-021	Afu6g09670:c.96+13T>C		-	Cluster_20_gliotoxin
Chr6	2359666	SNV	G	A	1	IF1SW-F4	Afu6g09670:c.97-10G>A		-	Cluster_20_gliotoxin
Chr6	2359692	SNV	A	T	1	CEA10	Afu6g09670:c.113A>T	Afu6g09670:p.Lys38Ile	Yes	Cluster_20_gliotoxin
Chr6	2359923	SNV	G	A	1	CEA10	Afu6g09670:c.288G>A		No	Cluster_20_gliotoxin
Chr6	2360490	SNV	G	A	1	CEA10	Afu6g09670:c.855G>A		No	Cluster_20_gliotoxin
Chr6	2361009	SNV	T	C	1	ISSFT-021	Afu6g09670:c.1218T>C		No	Cluster_20_gliotoxin
Chr6	2361117	SNV	T	C	1	CEA10, ISSFT-021, IF1SW-F4	Afu6g09670:c.1311+15T>C		-	Cluster_20_gliotoxin
Chr6	2361764	SNV	C	T	1	ISSFT-021	Afu6g09680:c.1195G>A	Afu6g09680:p.Glu399Lys	Yes	Cluster_20_gliotoxin
Chr6	2362817	SNV	T	C	1	CEA10	Afu6g09680:c.198A>G		No	Cluster_20_gliotoxin
Chr6	2363368	Replacemen	A	TATAT	5	CEA10, ISSFT-021, IF1SW-F4			-	Cluster_20_gliotoxin
Chr6	2363865	SNV	G	A	1	ISSFT-021, IF1SW-F4	Afu6g09690:c.303C>T		No	Cluster_20_gliotoxin
Chr6	2363870	SNV	G	A	1	CEA10, ISSFT-021, IF1SW-F4	Afu6g09690:c.298C>T	Afu6g09690:p.Leu100Phe	Yes	Cluster_20_gliotoxin
Chr6	2365191	SNV	T	C	1	ISSFT-021, IF1SW-F4	Afu6g09700:c.672T>C		No	Cluster_20_gliotoxin
Chr6	2365430	SNV	C	T	1	IF1SW-F4			-	Cluster_20_gliotoxin
Chr6	2365967	SNV	T	C	1	IF1SW-F4	Afu6g09710:c.38T>C	Afu6g09710:p.Leu13Pro	Yes	Cluster_20_gliotoxin
Chr6	2367208	SNV	C	T	1	ISSFT-021	Afu6g09710:c.1279C>T	Afu6g09710:p.Arg427Cys	Yes	Cluster_20_gliotoxin
Chr6	2367885	SNV	A	G	1	ISSFT-021, IF1SW-F4			-	Cluster_20_gliotoxin
Chr6	2368168	SNV	C	G	1	ISSFT-021, IF1SW-F4	Afu6g09720:c.696G>C		No	Cluster_20_gliotoxin
Chr6	2368564	SNV	C	T	1	ISSFT-021, IF1SW-F4	Afu6g09720:c.300G>A		No	Cluster_20_gliotoxin
Chr6	2368576	SNV	G	C	1	ISSFT-021, IF1SW-F4	Afu6g09720:c.288C>G		No	Cluster_20_gliotoxin
Chr6	2369116	SNV	T	C	1	ISSFT-021, IF1SW-F4			-	Cluster_20_gliotoxin
Chr6	2369257	SNV	A	C	1	ISSFT-021, IF1SW-F4	Afu6g09730:c.*109T>G		-	Cluster_20_gliotoxin
Chr6	2369490	SNV	G	A	1	CEA10	Afu6g09730:c.1391C>T	Afu6g09730:p.Ala464Val	Yes	Cluster_20_gliotoxin
Chr6	2370329	SNV	A	G	1	ISSFT-021, IF1SW-F4	Afu6g09730:c.552T>C		No	Cluster_20_gliotoxin
Chr6	2371725	SNV	G	A	1	IF1SW-F4	Afu6g09740:c.486G>A		No	Cluster_20_gliotoxin
Chr6	3010002^3010003	Insertion	-	CGCC	4	IF1SW-F4	Afu6g12040:c.-492_-491insCGCC		-	Cluster_21_fumiquinazolines
Chr6	3010149..3010160	Deletion	CTTGTCTATTGT	-	12	IF1SW-F4	fu6g12040:c.-345_-334delCTTGTCTATTG		-	Cluster_21_fumiquinazolines
Chr6	3010172	SNV	A	C	1	ISSFT-021	Afu6g12040:c.-322A>C		-	Cluster_21_fumiquinazolines
Chr6	3010181..3010184	MNV	CGAA	TGAT	4	IF1SW-F4	Afu6g12040:c.-313_-310delCGAAinsTGAT		-	Cluster_21_fumiquinazolines
Chr6	3010272	SNV	G	A	1	IF1SW-F4	Afu6g12040:c.-222G>A		-	Cluster_21_fumiquinazolines
Chr6	3010278	SNV	T	C	1	CEA10, ISSFT-021, IF1SW-F4	Afu6g12040:c.-216T>C		-	Cluster_21_fumiquinazolines
Chr6	3010288	SNV	T	C	1	IF1SW-F4	Afu6g12040:c.-206T>C		-	Cluster_21_fumiquinazolines
Chr6	3010325	SNV	G	C	1	IF1SW-F4	Afu6g12040:c.-169G>C		-	Cluster_21_fumiquinazolines
Chr6	3010469	SNV	T	C	1	ISSFT-021	Afu6g12040:c.-25T>C		-	Cluster_21_fumiquinazolines
Chr6	3010540	SNV	G	A	1	IF1SW-F4	Afu6g12040:c.47G>A	Afu6g12040:p.Arg16His	Yes	Cluster_21_fumiquinazolines
Chr6	3010622	SNV	G	A	1	IF1SW-F4	Afu6g12040:c.129G>A		No	Cluster_21_fumiquinazolines
Chr6	3010651	SNV	A	G	1	IF1SW-F4	Afu6g12040:c.154+4A>G		-	Cluster_21_fumiquinazolines
Chr6	3010688	SNV	T	C	1	ISSFT-021	Afu6g12040:c.155-34T>C		-	Cluster_21_fumiquinazolines
Chr6	3010855	SNV	T	C	1	IF1SW-F4	Afu6g12040:c.288T>C		No	Cluster_21_fumiquinazolines
Chr6	3011044	SNV	G	A	1	CEA10	Afu6g12040:c.477G>A	Afu6g12040:p.Met159Ile	Yes	Cluster_21_fumiquinazolines
Chr6	3011176	SNV	T	C	1	IF1SW-F4	Afu6g12040:c.542T>C	Afu6g12040:p.Ile181Thr	Yes	Cluster_21_fumiquinazolines
Chr6	3011297	SNV	C	T	1	IF1SW-F4	Afu6g12040:c.663C>T		No	Cluster_21_fumiquinazolines
Chr6	3011521	SNV	G	A	1	IF1SW-F4	Afu6g12040:c.826G>A	Afu6g12040:p.Ala276Thr	Yes	Cluster_21_fumiquinazolines
Chr6	3011566..3011570	MNV	TCAGT	ACAGC	5	IF1SW-F4	\fu6g12040:c.871_875delTCAGTinsACAG	6g12040:p.Ser291_Val292delinsThr	Yes	Cluster_21_fumiquinazolines
Chr6	3011570	SNV	T	C	1	CEA10, ISSFT-021, IF1SW-F4	Afu6g12040:c.875T>C	Afu6g12040:p.Val292Ala	Yes	Cluster_21_fumiquinazolines
Chr6	3011578	SNV	G	C	1	CEA10, ISSFT-021, IF1SW-F4	Afu6g12040:c.883G>C	Afu6g12040:p.Ala295Pro	Yes	Cluster_21_fumiquinazolines
Chr6	3011619	SNV	T	C	1	IF1SW-F4	Afu6g12040:c.924T>C		No	Cluster_21_fumiquinazolines
Chr6	3011737	SNV	G	A	1	IF1SW-F4	Afu6g12040:c.1042G>A	Afu6g12040:p.Gly348Arg	Yes	Cluster_21_fumiquinazolines
Chr6	3011757	SNV	C	T	1	IF1SW-F4	Afu6g12040:c.1062C>T		No	Cluster_21_fumiquinazolines
Chr6	3011793	SNV	C	T	1	IF1SW-F4	Afu6g12040:c.1098C>T		No	Cluster_21_fumiquinazolines
Chr6	3011796	SNV	T	C	1	CEA10	Afu6g12040:c.1101T>C		No	Cluster_21_fumiquinazolines
Chr6	3011854	SNV	G	A	1	CEA10	Afu6g12040:c.1159G>A	Afu6g12040:p.Gly387Ser	Yes	Cluster_21_fumiquinazolines
Chr6	3011862	SNV	G	T	1	CEA10	Afu6g12040:c.1167G>T	Afu6g12040:p.Leu389Phe	Yes	Cluster_21_fumiquinazolines
Chr6	3012170	SNV	T	C	1	IF1SW-F4	Afu6g12040:c.1410T>C		No	Cluster_21_fumiquinazolines
Chr6	3012330	SNV	A	G	1	IF1SW-F4	Afu6g12040:c.1570A>G	Afu6g12040:p.Ser524Gly	Yes	Cluster_21_fumiquinazolines
Chr6	3012408	SNV	T	G	1	IF1SW-F4	Afu6g12040:c.*14-8T>G		-	Cluster_21_fumiquinazolines
Chr6	3012614	SNV	C	A	1	IF1SW-F4	Afu6g12040:c.*212C>A		-	Cluster_21_fumiquinazolines

Chr6	3012640..3012645	MNV	AATACA	GCTACG	6	IF1SW-F4	6g12040:c.*238_*243delAATACAinsGCTA		-	Cluster_21_fumiquinazolines
Chr6	3012645	SNV	A	G	1	CEA10, ISSFT-021, IF1SW-F4	Afu6g12040:c.*243A>G		-	Cluster_21_fumiquinazolines
Chr6	3012680..3012681	MNV	CC	TT	2	IF1SW-F4	Afu6g12040:c.*278_*279delCCinsTT		-	Cluster_21_fumiquinazolines
Chr6	3012732	SNV	G	A	1	IF1SW-F4	Afu6g12040:c.*330G>A		-	Cluster_21_fumiquinazolines
Chr6	3012774	SNV	A	G	1	IF1SW-F4	Afu6g12040:c.*372A>G		-	Cluster_21_fumiquinazolines
Chr6	3012842	SNV	C	A	1	IF1SW-F4	Afu6g12040:c.*440C>A		-	Cluster_21_fumiquinazolines
Chr6	3013010	SNV	T	C	1	IF1SW-F4			-	Cluster_21_fumiquinazolines
Chr6	3013045	SNV	A	C	1	IF1SW-F4			-	Cluster_21_fumiquinazolines
Chr6	3013130	SNV	C	T	1	IF1SW-F4			-	Cluster_21_fumiquinazolines
Chr6	3013186..3013187	MNV	GT	CG	2	IF1SW-F4			-	Cluster_21_fumiquinazolines
Chr6	3013307	SNV	C	A	1	CEA10, IF1SW-F4			-	Cluster_21_fumiquinazolines
Chr6	3013313..3013315	MNV	TCG	CCT	3	IF1SW-F4			-	Cluster_21_fumiquinazolines
Chr6	3013434	SNV	C	T	1	IF1SW-F4			-	Cluster_21_fumiquinazolines
Chr6	3013446	SNV	T	C	1	CEA10			-	Cluster_21_fumiquinazolines
Chr6	3013447	SNV	G	A	1	IF1SW-F4			-	Cluster_21_fumiquinazolines
Chr6	3013577	SNV	A	G	1	IF1SW-F4			-	Cluster_21_fumiquinazolines
Chr6	3013619	SNV	G	A	1	IF1SW-F4	Afu6g12050:c.27G>A		No	Cluster_21_fumiquinazolines
Chr6	3013704	SNV	A	C	1	IF1SW-F4	Afu6g12050:c.87+25A>C		-	Cluster_21_fumiquinazolines
Chr6	3013984	SNV	A	G	1	IF1SW-F4	Afu6g12050:c.88-128A>G		-	Cluster_21_fumiquinazolines
Chr6	3013994	SNV	T	G	1	ISSFT-021	Afu6g12050:c.88-118T>G		-	Cluster_21_fumiquinazolines
Chr6	3014026	Deletion	C	-	1	IF1SW-F4	Afu6g12050:c.88-86delC		-	Cluster_21_fumiquinazolines
Chr6	3014156	SNV	C	T	1	IF1SW-F4	Afu6g12050:c.132C>T		No	Cluster_21_fumiquinazolines
Chr6	3014257	SNV	G	C	1	CEA10	Afu6g12050:c.233G>C	Afu6g12050:p.Ser78Thr	Yes	Cluster_21_fumiquinazolines
Chr6	3014378	SNV	G	C	1	IF1SW-F4	Afu6g12050:c.354G>C		No	Cluster_21_fumiquinazolines
Chr6	3014453..3014456	MNV	TGCC	CGCG	4	IF1SW-F4	Afu6g12050:c.429_432delTGCCinsCGCG		No	Cluster_21_fumiquinazolines
Chr6	3014564	SNV	C	A	1	IF1SW-F4	Afu6g12050:c.540C>A		No	Cluster_21_fumiquinazolines
Chr6	3014690	SNV	T	C	1	IF1SW-F4	Afu6g12050:c.666T>C		No	Cluster_21_fumiquinazolines
Chr6	3014735	SNV	A	G	1	IF1SW-F4	Afu6g12050:c.711A>G		No	Cluster_21_fumiquinazolines
Chr6	3014762	SNV	G	A	1	IF1SW-F4	Afu6g12050:c.738G>A		No	Cluster_21_fumiquinazolines
Chr6	3014837	SNV	C	A	1	IF1SW-F4	Afu6g12050:c.813C>A	Afu6g12050:p.Phe271Leu	Yes	Cluster_21_fumiquinazolines
Chr6	3014884	SNV	C	A	1	IF1SW-F4	Afu6g12050:c.860C>A	Afu6g12050:p.Ala287Asp	Yes	Cluster_21_fumiquinazolines
Chr6	3015110	SNV	A	T	1	IF1SW-F4	Afu6g12050:c.1086A>T		No	Cluster_21_fumiquinazolines
Chr6	3015335	SNV	A	G	1	ISSFT-021	Afu6g12050:c.1311A>G		No	Cluster_21_fumiquinazolines
Chr6	3015369	SNV	G	A	1	IF1SW-F4	Afu6g12050:c.1345G>A	Afu6g12050:p.Gly449Arg	Yes	Cluster_21_fumiquinazolines
Chr6	3015705..3015706	MNV	CA	AG	2	IF1SW-F4	Afu6g12050:c.1681_1682delCAinsAG	Afu6g12050:p.His561Ser	Yes	Cluster_21_fumiquinazolines
Chr6	3015752	SNV	C	G	1	IF1SW-F4	Afu6g12050:c.1728C>G		No	Cluster_21_fumiquinazolines
Chr6	3016172	SNV	C	T	1	IF1SW-F4	Afu6g12050:c.2148C>T		No	Cluster_21_fumiquinazolines
Chr6	3016195	SNV	A	G	1	CEA10, ISSFT-021, IF1SW-F4	Afu6g12050:c.2171A>G	Afu6g12050:p.Glu724Gly	Yes	Cluster_21_fumiquinazolines
Chr6	3016269	SNV	T	C	1	ISSFT-021	Afu6g12050:c.2245T>C	Afu6g12050:p.Cys749Arg	Yes	Cluster_21_fumiquinazolines
Chr6	3016626	SNV	A	G	1	ISSFT-021	Afu6g12050:c.2602A>G	Afu6g12050:p.Ser868Gly	Yes	Cluster_21_fumiquinazolines
Chr6	3017106	SNV	T	C	1	ISSFT-021, IF1SW-F4	Afu6g12050:c.3082T>C	Afu6g12050:p.Ser1028Pro	Yes	Cluster_21_fumiquinazolines
Chr6	3017122	SNV	T	C	1	CEA10	Afu6g12050:c.3098T>C	Afu6g12050:p.Val1033Ala	Yes	Cluster_21_fumiquinazolines
Chr6	3017137	SNV	A	G	1	IF1SW-F4	Afu6g12050:c.3113A>G	Afu6g12050:p.Lys1038Arg	Yes	Cluster_21_fumiquinazolines
Chr6	3017435	SNV	T	C	1	ISSFT-021	Afu6g12050:c.3411T>C		No	Cluster_21_fumiquinazolines
Chr6	3017711	SNV	T	G	1	ISSFT-021			-	Cluster_21_fumiquinazolines
Chr6	3018148	SNV	C	T	1	IF1SW-F4	Afu6g12060:c.1017G>A		No	Cluster_21_fumiquinazolines
Chr6	3018412	SNV	G	T	1	IF1SW-F4	Afu6g12060:c.774C>A	Afu6g12060:p.Asp258Glu	Yes	Cluster_21_fumiquinazolines
Chr6	3018647	SNV	C	T	1	ISSFT-021	Afu6g12060:c.606+22G>A		-	Cluster_21_fumiquinazolines
Chr6	3018798	SNV	C	T	1	IF1SW-F4	Afu6g12060:c.477G>A		No	Cluster_21_fumiquinazolines
Chr6	3019473	SNV	G	A	1	CEA10			-	Cluster_21_fumiquinazolines
Chr6	3019583	SNV	G	A	1	IF1SW-F4			-	Cluster_21_fumiquinazolines
Chr6	3019825	Deletion	T	-	1	IF1SW-F4			-	Cluster_21_fumiquinazolines
Chr6	3019943	SNV	C	T	1	IF1SW-F4			-	Cluster_21_fumiquinazolines
Chr6	3019975	SNV	T	C	1	IF1SW-F4			-	Cluster_21_fumiquinazolines
Chr6	3020059	SNV	A	G	1	IF1SW-F4			-	Cluster_21_fumiquinazolines
Chr6	3020074	SNV	A	C	1	IF1SW-F4			-	Cluster_21_fumiquinazolines
Chr6	3020094	SNV	A	G	1	IF1SW-F4			-	Cluster_21_fumiquinazolines
Chr6	3020421	SNV	A	G	1	IF1SW-F4	Afu6g12070:c.269A>G	Afu6g12070:p.Tyr90Cys	Yes	Cluster_21_fumiquinazolines
Chr6	3021154	SNV	T	G	1	IF1SW-F4	Afu6g12070:c.880-33T>G		-	Cluster_21_fumiquinazolines

Chr6	3021171	SNV	T	C	1	IF1SW-F4	Afu6g12070:c.880-16T>C		-	Cluster_21_fumiquinazolines
Chr6	3021612	SNV	C	T	1	IF1SW-F4	Afu6g12070:c.1242-62C>T		-	Cluster_21_fumiquinazolines
Chr6	3021646	SNV	C	T	1	IF1SW-F4	Afu6g12070:c.1242-28C>T		-	Cluster_21_fumiquinazolines
Chr6	3021680	SNV	T	C	1	IF1SW-F4	Afu6g12070:c.1248T>C		No	Cluster_21_fumiquinazolines
Chr6	3021748	SNV	G	A	1	IF1SW-F4	Afu6g12070:c.1316G>A	Afu6g12070:p.Arg439Gln	Yes	Cluster_21_fumiquinazolines
Chr6	3021914	SNV	G	C	1	ISSFT-021	Afu6g12070:c.1482G>C		No	Cluster_21_fumiquinazolines
Chr6	3021986	SNV	C	T	1	IF1SW-F4			-	Cluster_21_fumiquinazolines
Chr6	3022081..3022083	MNV	TGT	CGG	3	IF1SW-F4			-	Cluster_21_fumiquinazolines
Chr6	3022221..3022222	MNV	AA	CG	2	IF1SW-F4			-	Cluster_21_fumiquinazolines
Chr6	3022318	SNV	T	G	1	IF1SW-F4			-	Cluster_21_fumiquinazolines
Chr6	3022442	SNV	A	C	1	IF1SW-F4			-	Cluster_21_fumiquinazolines
Chr6	3022725	Deletion	A	-	1	IF1SW-F4			-	Cluster_21_fumiquinazolines
Chr6	3022758	SNV	T	C	1	IF1SW-F4			-	Cluster_21_fumiquinazolines
Chr6	3022794	SNV	A	G	1	IF1SW-F4			-	Cluster_21_fumiquinazolines
Chr6	3022904	SNV	G	A	1	IF1SW-F4			-	Cluster_21_fumiquinazolines
Chr6	3023019..3023020	MNV	AG	TA	2	IF1SW-F4			-	Cluster_21_fumiquinazolines
Chr6	3023117	SNV	T	A	1	IF1SW-F4	Afu6g12080:c.*199A>T		-	Cluster_21_fumiquinazolines
Chr6	3023280	SNV	A	G	1	CEA10, ISSFT-021, IF1SW-F4	Afu6g12080:c.*36T>C		-	Cluster_21_fumiquinazolines
Chr6	3023357	SNV	C	G	1	IF1SW-F4	Afu6g12080:c.11827G>C	Afu6g12080:p.Asp3943His	Yes	Cluster_21_fumiquinazolines
Chr6	3023550	SNV	T	C	1	IF1SW-F4	Afu6g12080:c.11634A>G		No	Cluster_21_fumiquinazolines
Chr6	3023663	SNV	A	C	1	IF1SW-F4	Afu6g12080:c.11521T>G	Afu6g12080:p.Ser3841Ala	Yes	Cluster_21_fumiquinazolines
Chr6	3023679	SNV	C	G	1	IF1SW-F4	Afu6g12080:c.11505G>C		No	Cluster_21_fumiquinazolines
Chr6	3023688	SNV	G	T	1	IF1SW-F4	Afu6g12080:c.11496C>A		No	Cluster_21_fumiquinazolines
Chr6	3023885	SNV	G	C	1	CEA10	Afu6g12080:c.11299C>G	Afu6g12080:p.Leu3767Val	Yes	Cluster_21_fumiquinazolines
Chr6	3024158	SNV	C	T	1	IF1SW-F4	Afu6g12080:c.11026G>A	Afu6g12080:p.Ala3676Thr	Yes	Cluster_21_fumiquinazolines
Chr6	3024363	SNV	A	T	1	CEA10	Afu6g12080:c.10821T>A		No	Cluster_21_fumiquinazolines
Chr6	3024792	SNV	C	A	1	CEA10	Afu6g12080:c.10392G>T	Afu6g12080:p.Lys3464Asn	Yes	Cluster_21_fumiquinazolines
Chr6	3025176	SNV	A	G	1	IF1SW-F4	Afu6g12080:c.10008T>C		No	Cluster_21_fumiquinazolines
Chr6	3025758	SNV	A	G	1	IF1SW-F4	Afu6g12080:c.9426T>C		No	Cluster_21_fumiquinazolines
Chr6	3025947	SNV	G	A	1	CEA10, IF1SW-F4	Afu6g12080:c.9237C>T		No	Cluster_21_fumiquinazolines
Chr6	3026792	SNV	G	C	1	IF1SW-F4	Afu6g12080:c.8401-9C>G		-	Cluster_21_fumiquinazolines
Chr6	3027112	SNV	A	G	1	IF1SW-F4	Afu6g12080:c.8127T>C		No	Cluster_21_fumiquinazolines
Chr6	3027307	SNV	C	G	1	IF1SW-F4	Afu6g12080:c.7932G>C	Afu6g12080:p.Lys2644Asn	Yes	Cluster_21_fumiquinazolines
Chr6	3027346	SNV	T	C	1	IF1SW-F4	Afu6g12080:c.7893A>G		No	Cluster_21_fumiquinazolines
Chr6	3027481	SNV	T	A	1	IF1SW-F4	Afu6g12080:c.7758A>T		No	Cluster_21_fumiquinazolines
Chr6	3027665	SNV	C	G	1	CEA10, ISSFT-021, IF1SW-F4	Afu6g12080:c.7574G>C	Afu6g12080:p.Cys2525Ser	Yes	Cluster_21_fumiquinazolines
Chr6	3027766	SNV	T	C	1	IF1SW-F4	Afu6g12080:c.7473A>G		No	Cluster_21_fumiquinazolines
Chr6	3027836	SNV	C	A	1	IF1SW-F4	Afu6g12080:c.7403G>T	Afu6g12080:p.Ser2468Ile	Yes	Cluster_21_fumiquinazolines
Chr6	3027895	SNV	G	T	1	IF1SW-F4	Afu6g12080:c.7344C>A		No	Cluster_21_fumiquinazolines
Chr6	3028061	SNV	C	T	1	IF1SW-F4	Afu6g12080:c.7178G>A	Afu6g12080:p.Gly2393Glu	Yes	Cluster_21_fumiquinazolines
Chr6	3028367	SNV	A	G	1	IF1SW-F4	Afu6g12080:c.6872T>C	Afu6g12080:p.Val2291Ala	Yes	Cluster_21_fumiquinazolines
Chr6	3028621	SNV	G	A	1	CEA10, ISSFT-021	Afu6g12080:c.6618C>T		No	Cluster_21_fumiquinazolines
Chr6	3029113	SNV	C	T	1	IF1SW-F4	Afu6g12080:c.6126G>A		No	Cluster_21_fumiquinazolines
Chr6	3029550	SNV	A	G	1	IF1SW-F4	Afu6g12080:c.5689T>C		No	Cluster_21_fumiquinazolines
Chr6	3029899	SNV	G	A	1	IF1SW-F4	Afu6g12080:c.5340C>T		No	Cluster_21_fumiquinazolines
Chr6	3030079	SNV	C	G	1	IF1SW-F4	Afu6g12080:c.5160G>C		No	Cluster_21_fumiquinazolines
Chr6	3030211	SNV	G	T	1	IF1SW-F4	Afu6g12080:c.5028C>A		No	Cluster_21_fumiquinazolines
Chr6	3031048	SNV	A	T	1	IF1SW-F4	Afu6g12080:c.4191T>A		No	Cluster_21_fumiquinazolines
Chr6	3031217	SNV	G	C	1	IF1SW-F4	Afu6g12080:c.4022C>G	Afu6g12080:p.Ala1341Gly	Yes	Cluster_21_fumiquinazolines
Chr6	3031567	SNV	C	T	1	IF1SW-F4	Afu6g12080:c.3672G>A		No	Cluster_21_fumiquinazolines
Chr6	3032110	SNV	G	A	1	IF1SW-F4	Afu6g12080:c.3129C>T		No	Cluster_21_fumiquinazolines
Chr6	3032269	SNV	C	T	1	ISSFT-021	Afu6g12080:c.2970G>A		No	Cluster_21_fumiquinazolines
Chr6	3032366	SNV	C	G	1	CEA10, ISSFT-021	Afu6g12080:c.2873G>C	Afu6g12080:p.Arg958Thr	Yes	Cluster_21_fumiquinazolines
Chr6	3032811	SNV	T	C	1	IF1SW-F4	Afu6g12080:c.2428A>G	Afu6g12080:p.Thr810Ala	Yes	Cluster_21_fumiquinazolines
Chr6	3032962	SNV	G	C	1	IF1SW-F4	Afu6g12080:c.2277C>G		No	Cluster_21_fumiquinazolines
Chr6	3033636	SNV	A	G	1	CEA10	Afu6g12080:c.1603T>C	Afu6g12080:p.Phe535Leu	Yes	Cluster_21_fumiquinazolines
Chr6	3033682	SNV	A	G	1	IF1SW-F4	Afu6g12080:c.1557T>C		No	Cluster_21_fumiquinazolines
Chr6	3033739	SNV	G	A	1	IF1SW-F4	Afu6g12080:c.1500C>T		No	Cluster_21_fumiquinazolines
Chr6	3034027	SNV	C	A	1	IF1SW-F4	Afu6g12080:c.1212G>T		No	Cluster_21_fumiquinazolines

Chr6	3034114	SNV	A	G	1	IF1SW-F4	Afu6g12080:c.1125T>C		No	Cluster_21_fumiquinazolines
Chr6	3034210	SNV	A	G	1	CEA10, ISSFT-021, IF1SW-F4	Afu6g12080:c.1029T>C		No	Cluster_21_fumiquinazolines
Chr6	3034294	SNV	G	A	1	IF1SW-F4	Afu6g12080:c.945C>T		No	Cluster_21_fumiquinazolines
Chr6	3034367	SNV	G	A	1	IF1SW-F4	Afu6g12080:c.872C>T	Afu6g12080:p.Ala291Val	Yes	Cluster_21_fumiquinazolines
Chr6	3034584	SNV	G	A	1	IF1SW-F4	Afu6g12080:c.655C>T		No	Cluster_21_fumiquinazolines
Chr6	3034639	SNV	C	T	1	IF1SW-F4	Afu6g12080:c.600G>A		No	Cluster_21_fumiquinazolines
Chr6	3034801	SNV	C	T	1	IF1SW-F4	Afu6g12080:c.505G>A	Afu6g12080:p.Ala169Thr	Yes	Cluster_21_fumiquinazolines
Chr6	3034819	SNV	T	A	1	IF1SW-F4	Afu6g12080:c.487A>T	Afu6g12080:p.Asn163Tyr	Yes	Cluster_21_fumiquinazolines
Chr6	3034877	SNV	C	T	1	IF1SW-F4	Afu6g12080:c.429G>A		No	Cluster_21_fumiquinazolines
Chr6	3035096	SNV	T	C	1	IF1SW-F4	Afu6g12080:c.210A>G		No	Cluster_21_fumiquinazolines
Chr6	3035276	SNV	A	G	1	IF1SW-F4	Afu6g12080:c.30T>C		No	Cluster_21_fumiquinazolines
Chr6	3548131	SNV	G	A	1	ISSFT-021			-	Cluster_22_pyripyropene_A
Chr6	3548155..3548157	MNV	CGT	TGA	3	IF1SW-F4			-	Cluster_22_pyripyropene_A
Chr6	3548295	SNV	C	T	1	IF1SW-F4			-	Cluster_22_pyripyropene_A
Chr6	3548937	SNV	T	C	1	CEA10, ISSFT-021, IF1SW-F4	Afu6g13920:c.*126A>G		-	Cluster_22_pyripyropene_A
Chr6	3550154	SNV	T	G	1	CEA10, ISSFT-021, IF1SW-F4	Afu6g13920:c.655A>C	Afu6g13920:p.Ser219Arg	Yes	Cluster_22_pyripyropene_A
Chr6	3550337	SNV	C	G	1	ISSFT-021	Afu6g13920:c.472G>C	Afu6g13920:p.Gly158Arg	Yes	Cluster_22_pyripyropene_A
Chr6	3550993	SNV	G	A	1	CEA10, ISSFT-021, IF1SW-F4			-	Cluster_22_pyripyropene_A
Chr6	3551007^3551008	Insertion	-	C	1	ISSFT-021			-	Cluster_22_pyripyropene_A
Chr6	3551069	SNV	A	G	1	CEA10, ISSFT-021, IF1SW-F4	Afu6g13930:c.*35T>C		-	Cluster_22_pyripyropene_A
Chr6	3553132	SNV	C	T	1	CEA10, ISSFT-021, IF1SW-F4	Afu6g13930:c.5364G>A		No	Cluster_22_pyripyropene_A
Chr6	3553709	SNV	T	C	1	ISSFT-021	Afu6g13930:c.4787A>G	Afu6g13930:p.Glu1596Gly	Yes	Cluster_22_pyripyropene_A
Chr6	3554511	SNV	C	G	1	IF1SW-F4	Afu6g13930:c.3985G>C	Afu6g13930:p.Val1329Leu	Yes	Cluster_22_pyripyropene_A
Chr6	3554730	SNV	T	C	1	ISSFT-021, IF1SW-F4	Afu6g13930:c.3766A>G	Afu6g13930:p.Asn1256Asp	Yes	Cluster_22_pyripyropene_A
Chr6	3554980	SNV	G	A	1	IF1SW-F4	Afu6g13930:c.3516C>T		No	Cluster_22_pyripyropene_A
Chr6	3555254	SNV	A	G	1	ISSFT-021, IF1SW-F4	Afu6g13930:c.3242T>C	Afu6g13930:p.Val1081Ala	Yes	Cluster_22_pyripyropene_A
Chr6	3555709	SNV	A	G	1	ISSFT-021, IF1SW-F4	Afu6g13930:c.2787T>C		No	Cluster_22_pyripyropene_A
Chr6	3555939	SNV	T	C	1	ISSFT-021, IF1SW-F4	Afu6g13930:c.2557A>G	Afu6g13930:p.Thr853Ala	Yes	Cluster_22_pyripyropene_A
Chr6	3556326	SNV	G	T	1	ISSFT-021	Afu6g13930:c.2170C>A	Afu6g13930:p.Leu724Met	Yes	Cluster_22_pyripyropene_A
Chr6	3556835	SNV	A	C	1	IF1SW-F4	Afu6g13930:c.1661T>G	Afu6g13930:p.Val554Gly	Yes	Cluster_22_pyripyropene_A
Chr6	3557032	SNV	C	T	1	IF1SW-F4	Afu6g13930:c.1464G>A		No	Cluster_22_pyripyropene_A
Chr6	3557368	SNV	C	G	1	IF1SW-F4	Afu6g13930:c.1128G>C		No	Cluster_22_pyripyropene_A
Chr6	3557478	SNV	T	C	1	ISSFT-021, IF1SW-F4	Afu6g13930:c.1018A>G	Afu6g13930:p.Lys340Glu	Yes	Cluster_22_pyripyropene_A
Chr6	3557480	SNV	G	T	1	CEA10	Afu6g13930:c.1016C>A	Afu6g13930:p.Pro339His	Yes	Cluster_22_pyripyropene_A
Chr6	3558646	SNV	A	G	1	ISSFT-021	Afu6g13930:c.-151T>C		-	Cluster_22_pyripyropene_A
Chr6	3558909	SNV	T	C	1	ISSFT-021	Afu6g13940:c.-38T>C		-	Cluster_22_pyripyropene_A
Chr6	3559035	SNV	G	A	1	CEA10, ISSFT-021	Afu6g13940:c.89G>A	Afu6g13940:p.Ser30Asn	Yes	Cluster_22_pyripyropene_A
Chr6	3559276	SNV	C	G	1	ISSFT-021, IF1SW-F4	Afu6g13940:c.295-33C>G		-	Cluster_22_pyripyropene_A
Chr6	3560302	SNV	A	G	1	ISSFT-021	Afu6g13940:c.1097A>G	Afu6g13940:p.Asp366Gly	Yes	Cluster_22_pyripyropene_A
Chr6	3560561	SNV	C	G	1	CEA10, ISSFT-021, IF1SW-F4	Afu6g13940:c.1291C>G	Afu6g13940:p.Gln431Glu	Yes	Cluster_22_pyripyropene_A
Chr6	3561273	SNV	A	G	1	IF1SW-F4			-	Cluster_22_pyripyropene_A
Chr6	3561633	SNV	A	G	1	CEA10, ISSFT-021, IF1SW-F4	Afu6g13945:c.-77A>G		-	Cluster_22_pyripyropene_A
Chr6	3561941	SNV	A	G	1	CEA10, ISSFT-021, IF1SW-F4	Afu6g13945:c.213+19A>G		-	Cluster_22_pyripyropene_A
Chr6	3561954	SNV	C	T	1	ISSFT-021	Afu6g13945:c.214-28C>T		-	Cluster_22_pyripyropene_A
Chr6	3562855	SNV	G	A	1	ISSFT-021	Afu6g13945:c.937G>A	Afu6g13945:p.Gly313Ser	Yes	Cluster_22_pyripyropene_A
Chr6	3563010	SNV	T	C	1	CEA10, ISSFT-021, IF1SW-F4	Afu6g13945:c.1072+20T>C		-	Cluster_22_pyripyropene_A
Chr6	3563498	SNV	T	C	1	CEA10, ISSFT-021, IF1SW-F4	Afu6g13945:c.*12T>C		-	Cluster_22_pyripyropene_A
Chr6	3563749	SNV	C	G	1	IF1SW-F4			-	Cluster_22_pyripyropene_A
Chr6	3565451	SNV	G	A	1	CEA10, ISSFT-021	Afu6g13970:c.279-7G>A		-	Cluster_22_pyripyropene_A
Chr6	3565767	SNV	C	T	1	CEA10	Afu6g13970:c.547+41C>T		-	Cluster_22_pyripyropene_A
Chr6	3566335	SNV	C	A	1	CEA10, ISSFT-021, IF1SW-F4	Afu6g13970:c.981C>A		No	Cluster_22_pyripyropene_A
Chr6	3567085	SNV	G	A	1	CEA10			-	Cluster_22_pyripyropene_A
Chr6	3567228..3567234	Replacemen	CCCCCCC	T	7	CEA10	u6g13980:c.97+25_97+31delCCCCCCCin		-	Cluster_22_pyripyropene_A
Chr6	3567399	SNV	G	A	1	CEA10, ISSFT-021, IF1SW-F4	Afu6g13980:c.219G>A		No	Cluster_22_pyripyropene_A
Chr6	3568692..3568693	MNV	TC	AG	2	CEA10, ISSFT-021, IF1SW-F4	Afu6g13990:c.1326_1327delGAinsCT	6g13990:p.Met442_Ile443delinsIle	Yes	Cluster_22_pyripyropene_A
Chr6	3569784	SNV	C	T	1	ISSFT-021, IF1SW-F4	Afu6g13990:c.235G>A	Afu6g13990:p.Ala79Thr	Yes	Cluster_22_pyripyropene_A
Chr6	3570142	SNV	C	T	1	CEA10			-	Cluster_22_pyripyropene_A
Chr6	3570730	SNV	T	G	1	CEA10	Afu6g14000:c.-39T>G		-	Cluster_22_pyripyropene_A
Chr6	3571601	SNV	T	G	1	CEA10, ISSFT-021	Afu6g14000:c.789-39T>G		-	Cluster_22_pyripyropene_A

Chr6	3571978	SNV	A	G	1	CEA10, ISSFT-021, IF1SW-F4	Afu6g14000:c.1127A>G	Afu6g14000:p.Tyr376Cys	Yes	Cluster_22_pyripyropene_A
Chr6	3572138	SNV	T	C	1	CEA10, ISSFT-021, IF1SW-F4	Afu6g14000:c.1287T>C		No	Cluster_22_pyripyropene_A
Chr6	3572149	SNV	A	C	1	CEA10, ISSFT-021, IF1SW-F4	Afu6g14000:c.*8A>C		-	Cluster_22_pyripyropene_A
Chr6	3572384	SNV	A	G	1	CEA10, ISSFT-021, IF1SW-F4	Afu6g14000:c.*243A>G		-	Cluster_22_pyripyropene_A
Chr6	3572512^3572513	Insertion	-	T	1	CEA10, ISSFT-021, IF1SW-F4	Afu6g14000:c.*371_*372insT		-	Cluster_22_pyripyropene_A
Chr6	3572597	SNV	C	G	1	CEA10, ISSFT-021, IF1SW-F4	Afu6g14000:c.*456C>G		-	Cluster_22_pyripyropene_A
Chr7	29739	SNV	A	G	1	CEA10, ISSFT-021, IF1SW-F4	Afu7g00130:c.2007T>C		No	Cluster_23_neosartorycin
Chr7	32521	SNV	G	A	1	ISSFT-021			-	Cluster_23_neosartorycin
Chr7	33195	SNV	G	T	1	CEA10, IF1SW-F4	Afu7g00150:c.*494C>A		-	Cluster_23_neosartorycin
Chr7	33372	SNV	C	T	1	CEA10, ISSFT-021, IF1SW-F4	Afu7g00150:c.*317G>A		-	Cluster_23_neosartorycin
Chr7	34643	SNV	A	T	1	CEA10, ISSFT-021, IF1SW-F4	Afu7g00150:c.326T>A	Afu7g00150:p.Leu109Gln	Yes	Cluster_23_neosartorycin
Chr7	34950	SNV	T	C	1	ISSFT-021	Afu7g00150:c.19A>G	Afu7g00150:p.Ile7Val	Yes	Cluster_23_neosartorycin
Chr7	35470	SNV	T	C	1	ISSFT-021			-	Cluster_23_neosartorycin
Chr7	36305	SNV	A	C	1	CEA10, ISSFT-021, IF1SW-F4	Afu7g00160:c.5097T>G	Afu7g00160:p.Asp1699Glu	Yes	Cluster_23_neosartorycin
Chr7	37848	SNV	T	C	1	CEA10, ISSFT-021, IF1SW-F4	Afu7g00160:c.3631A>G	Afu7g00160:p.Ser1211Gly	Yes	Cluster_23_neosartorycin
Chr7	38619	SNV	A	G	1	CEA10, ISSFT-021, IF1SW-F4	Afu7g00160:c.2860T>C	Afu7g00160:p.Phe954Leu	Yes	Cluster_23_neosartorycin
Chr7	39426..39427	MNV	CC	AA	2	ISSFT-021	Afu7g00160:c.2052_2053delGGinsTT	Afu7g00160:p.Gly685Cys	Yes	Cluster_23_neosartorycin
Chr7	39537	SNV	A	G	1	CEA10, ISSFT-021, IF1SW-F4	Afu7g00160:c.1942T>C		No	Cluster_23_neosartorycin
Chr7	40573	SNV	G	T	1	CEA10, ISSFT-021, IF1SW-F4	Afu7g00160:c.962C>A	Afu7g00160:p.Thr321Asn	Yes	Cluster_23_neosartorycin
Chr7	41470	SNV	T	C	1	CEA10, ISSFT-021, IF1SW-F4	Afu7g00160:c.120A>G		No	Cluster_23_neosartorycin
Chr7	42157	SNV	C	G	1	ISSFT-021	Afu7g00170:c.87C>G		No	Cluster_23_neosartorycin
Chr7	43867	SNV	A	G	1	CEA10, ISSFT-021, IF1SW-F4			-	Cluster_23_neosartorycin
Chr7	44095	SNV	C	A	1	ISSFT-021	Afu7g00180:c.882G>T	Afu7g00180:p.Leu294Phe	Yes	Cluster_23_neosartorycin
Chr7	44777	SNV	G	A	1	CEA10, IF1SW-F4	Afu7g00180:c.200C>T	Afu7g00180:p.Ala67Val	Yes	Cluster_23_neosartorycin
Chr7	44956	SNV	A	G	1	ISSFT-021	Afu7g00180:c.21T>C		No	Cluster_23_neosartorycin
Chr8	21198	SNV	A	G	1	CEA10, ISSFT-021, IF1SW-F4	Afu8g00170:c.6292T>C	Afu8g00170:p.Phe2098Leu	Yes	Cluster_24_fumitremorgin
Chr8	21523	SNV	C	T	1	CEA10, ISSFT-021, IF1SW-F4	Afu8g00170:c.5967G>A		No	Cluster_24_fumitremorgin
Chr8	21666	SNV	G	T	1	CEA10, ISSFT-021, IF1SW-F4	Afu8g00170:c.5824C>A	Afu8g00170:p.Leu1942Ile	Yes	Cluster_24_fumitremorgin
Chr8	22187	SNV	G	A	1	CEA10, ISSFT-021, IF1SW-F4	Afu8g00170:c.5303C>T	Afu8g00170:p.Thr1768Ile	Yes	Cluster_24_fumitremorgin
Chr8	23038	SNV	G	C	1	CEA10, ISSFT-021, IF1SW-F4	Afu8g00170:c.4452C>G		No	Cluster_24_fumitremorgin
Chr8	23709	SNV	C	T	1	CEA10, ISSFT-021, IF1SW-F4	Afu8g00170:c.3781G>A	Afu8g00170:p.Val1261Ile	Yes	Cluster_24_fumitremorgin
Chr8	23947	SNV	C	G	1	CEA10, ISSFT-021, IF1SW-F4	Afu8g00170:c.3543G>C		No	Cluster_24_fumitremorgin
Chr8	24526	SNV	G	A	1	CEA10, ISSFT-021, IF1SW-F4	Afu8g00170:c.2964C>T		No	Cluster_24_fumitremorgin
Chr8	24618	SNV	C	T	1	CEA10, ISSFT-021, IF1SW-F4	Afu8g00170:c.2872G>A	Afu8g00170:p.Val958Ile	Yes	Cluster_24_fumitremorgin
Chr8	24808	SNV	A	G	1	CEA10, ISSFT-021, IF1SW-F4	Afu8g00170:c.2682T>C		No	Cluster_24_fumitremorgin
Chr8	24881	SNV	T	G	1	CEA10, ISSFT-021, IF1SW-F4	Afu8g00170:c.2609A>C	Afu8g00170:p.Gln870Pro	Yes	Cluster_24_fumitremorgin
Chr8	24955	SNV	A	C	1	CEA10, ISSFT-021, IF1SW-F4	Afu8g00170:c.2535T>G	Afu8g00170:p.His845Gln	Yes	Cluster_24_fumitremorgin
Chr8	25238	SNV	C	T	1	CEA10, ISSFT-021, IF1SW-F4	Afu8g00170:c.2252G>A	Afu8g00170:p.Arg751Lys	Yes	Cluster_24_fumitremorgin
Chr8	25543	SNV	T	G	1	CEA10, ISSFT-021, IF1SW-F4	Afu8g00170:c.1947A>C	Afu8g00170:p.Gln649His	Yes	Cluster_24_fumitremorgin
Chr8	25646	SNV	A	G	1	CEA10	Afu8g00170:c.1844T>C	Afu8g00170:p.Ile615Thr	Yes	Cluster_24_fumitremorgin
Chr8	25871	SNV	T	C	1	CEA10, ISSFT-021, IF1SW-F4	Afu8g00170:c.1619A>G	Afu8g00170:p.His540Arg	Yes	Cluster_24_fumitremorgin
Chr8	26281	SNV	C	G	1	CEA10, ISSFT-021, IF1SW-F4	Afu8g00170:c.1209G>C		No	Cluster_24_fumitremorgin
Chr8	26680	SNV	G	A	1	CEA10, ISSFT-021, IF1SW-F4	Afu8g00170:c.810C>T		No	Cluster_24_fumitremorgin
Chr8	27801	SNV	T	C	1	CEA10, ISSFT-021, IF1SW-F4			-	Cluster_24_fumitremorgin
Chr8	28034	SNV	G	T	1	CEA10, ISSFT-021, IF1SW-F4			-	Cluster_24_fumitremorgin
Chr8	31502^31503	Insertion	-	A	1	CEA10, ISSFT-021			-	Cluster_24_fumitremorgin
Chr8	32217	SNV	T	C	1	CEA10, ISSFT-021, IF1SW-F4	Afu8g00200:c.480T>C		No	Cluster_24_fumitremorgin
Chr8	32427	SNV	T	G	1	CEA10, ISSFT-021, IF1SW-F4	Afu8g00200:c.605T>G	Afu8g00200:p.Leu202Arg	Yes	Cluster_24_fumitremorgin
Chr8	32568	SNV	C	T	1	IF1SW-F4	Afu8g00200:c.746C>T	Afu8g00200:p.Ala249Val	Yes	Cluster_24_fumitremorgin
Chr8	32912	SNV	C	T	1	CEA10, ISSFT-021, IF1SW-F4			-	Cluster_24_fumitremorgin
Chr8	32973	SNV	G	A	1	IF1SW-F4			-	Cluster_24_fumitremorgin
Chr8	33624	SNV	G	T	1	CEA10, ISSFT-021, IF1SW-F4	Afu8g00210:c.258G>T		No	Cluster_24_fumitremorgin
Chr8	34470	SNV	A	G	1	CEA10, ISSFT-021, IF1SW-F4	Afu8g00210:c.1104A>G		No	Cluster_24_fumitremorgin
Chr8	35077	SNV	T	G	1	CEA10, ISSFT-021, IF1SW-F4			-	Cluster_24_fumitremorgin
Chr8	37235	Deletion	G	-	1	ISSFT-021, IF1SW-F4			-	Cluster_24_fumitremorgin
Chr8	37593..37594	MNV	GG	AA	2	CEA10	Afu8g00230:c.*78_*79delCCinsTT		-	Cluster_24_fumitremorgin
Chr8	37885	SNV	A	G	1	CEA10, ISSFT-021, IF1SW-F4	Afu8g00230:c.663T>C		No	Cluster_24_fumitremorgin
Chr8	38106	SNV	C	T	1	CEA10, ISSFT-021, IF1SW-F4	Afu8g00230:c.442G>A	Afu8g00230:p.Val148Ile	Yes	Cluster_24_fumitremorgin
Chr8	38283	SNV	C	T	1	CEA10, ISSFT-021, IF1SW-F4	Afu8g00230:c.265G>A	Afu8g00230:p.Val89Ile	Yes	Cluster_24_fumitremorgin

Chr8	38602	SNV	C	T	1	CEA10, ISSFT-021, IF1SW-F4	Afu8g00230:c.-55G>A		-	Cluster_24_fumitremorgin
Chr8	38646	SNV	G	C	1	CEA10, ISSFT-021, IF1SW-F4	Afu8g00230:c.-99C>G		-	Cluster_24_fumitremorgin
Chr8	38756	SNV	C	A	1	CEA10, ISSFT-021, IF1SW-F4	Afu8g00230:c.-209G>T		-	Cluster_24_fumitremorgin
Chr8	38756..38759	MNV	CTCA	ATTT	4	IF1SW-F4	Afu8g00230:c.-212_-209delTGAGinsAAAT		-	Cluster_24_fumitremorgin
Chr8	38765	SNV	C	T	1	CEA10, ISSFT-021, IF1SW-F4	Afu8g00230:c.-218G>A		-	Cluster_24_fumitremorgin
Chr8	39110	SNV	T	C	1	CEA10, ISSFT-021, IF1SW-F4			-	Cluster_24_fumitremorgin
Chr8	39726	SNV	C	T	1	CEA10, ISSFT-021	Afu8g00240:c.1426+5G>A		-	Cluster_24_fumitremorgin
Chr8	40228	SNV	T	C	1	CEA10, ISSFT-021, IF1SW-F4	Afu8g00240:c.998A>G	Afu8g00240:p.His333Arg	Yes	Cluster_24_fumitremorgin
Chr8	40972	SNV	G	A	1	CEA10, ISSFT-021, IF1SW-F4	Afu8g00240:c.357C>T		No	Cluster_24_fumitremorgin
Chr8	41738	SNV	G	A	1	CEA10, ISSFT-021, IF1SW-F4			-	Cluster_24_fumitremorgin
Chr8	41957	SNV	G	C	1	CEA10			-	Cluster_24_fumitremorgin
Chr8	42172	SNV	G	C	1	CEA10, ISSFT-021, IF1SW-F4	Afu8g00250:c.-10G>C		-	Cluster_24_fumitremorgin
Chr8	42675	SNV	A	G	1	CEA10	Afu8g00250:c.494A>G	Afu8g00250:p.Lys165Arg	Yes	Cluster_24_fumitremorgin
Chr8	42692	SNV	G	C	1	CEA10, ISSFT-021, IF1SW-F4	Afu8g00250:c.511G>C	Afu8g00250:p.Ala171Pro	Yes	Cluster_24_fumitremorgin
Chr8	42909	SNV	C	T	1	ISSFT-021	Afu8g00250:c.728C>T	Afu8g00250:p.Pro243Leu	Yes	Cluster_24_fumitremorgin
Chr8	43247	SNV	C	T	1	ISSFT-021, IF1SW-F4	Afu8g00250:c.1066C>T	Afu8g00250:p.Leu356Phe	Yes	Cluster_24_fumitremorgin
Chr8	43590	SNV	C	G	1	IF1SW-F4			-	Cluster_24_fumitremorgin
Chr8	43750	SNV	A	G	1	CEA10			-	Cluster_24_fumitremorgin
Chr8	43769..43772	Replacemen	AAGC	GCCCTTGAC	16	CEA10			-	Cluster_24_fumitremorgin
Chr8	43853	SNV	A	G	1	CEA10	Afu8g00260:c.2025T>C		No	Cluster_24_fumitremorgin
Chr8	44080	SNV	G	T	1	CEA10, ISSFT-021, IF1SW-F4	Afu8g00260:c.1798C>A	Afu8g00260:p.His600Asn	Yes	Cluster_24_fumitremorgin
Chr8	44188	SNV	G	A	1	ISSFT-021	Afu8g00260:c.1690C>T	Afu8g00260:p.Leu564Phe	Yes	Cluster_24_fumitremorgin
Chr8	44195	SNV	A	G	1	CEA10, ISSFT-021, IF1SW-F4	Afu8g00260:c.1683T>C		No	Cluster_24_fumitremorgin
Chr8	44449	SNV	C	T	1	ISSFT-021	Afu8g00260:c.1429G>A	Afu8g00260:p.Val477Ile	Yes	Cluster_24_fumitremorgin
Chr8	44514	SNV	C	T	1	CEA10	Afu8g00260:c.1364G>A	Afu8g00260:p.Cys455Tyr	Yes	Cluster_24_fumitremorgin
Chr8	44609	SNV	G	A	1	CEA10	Afu8g00260:c.1269C>T		No	Cluster_24_fumitremorgin
Chr8	44655	SNV	C	A	1	CEA10	Afu8g00260:c.1223G>T	Afu8g00260:p.Arg408Leu	Yes	Cluster_24_fumitremorgin
Chr8	44663	SNV	G	C	1	CEA10	Afu8g00260:c.1215C>G		No	Cluster_24_fumitremorgin
Chr8	44800	SNV	T	A	1	ISSFT-021, IF1SW-F4	Afu8g00260:c.1078A>T	Afu8g00260:p.Lys360*	Yes	Cluster_24_fumitremorgin
Chr8	44897	SNV	G	A	1	CEA10	Afu8g00260:c.981C>T		No	Cluster_24_fumitremorgin
Chr8	45089	SNV	G	A	1	CEA10	Afu8g00260:c.789C>T		No	Cluster_24_fumitremorgin
Chr8	45123	SNV	G	T	1	CEA10	Afu8g00260:c.755C>A	Afu8g00260:p.Thr252Asn	Yes	Cluster_24_fumitremorgin
Chr8	45727	SNV	T	C	1	CEA10	Afu8g00260:c.151A>G	Afu8g00260:p.Lys51Glu	Yes	Cluster_24_fumitremorgin
Chr8	79291	SNV	G	C	1	ISSFT-021, IF1SW-F4	Afu8g00370:c.7383C>G		No	Cluster_25_fumagillin/pseurotin
Chr8	79653	SNV	A	T	1	CEA10, ISSFT-021, IF1SW-F4	Afu8g00370:c.7021T>A	Afu8g00370:p.Cys2341Ser	Yes	Cluster_25_fumagillin/pseurotin
Chr8	80449	SNV	C	A	1	CEA10	Afu8g00370:c.6288G>T		No	Cluster_25_fumagillin/pseurotin
Chr8	80497	SNV	T	G	1	CEA10, ISSFT-021, IF1SW-F4	Afu8g00370:c.6240A>C		No	Cluster_25_fumagillin/pseurotin
Chr8	81408	SNV	G	T	1	CEA10, ISSFT-021, IF1SW-F4	Afu8g00370:c.5421C>A		No	Cluster_25_fumagillin/pseurotin
Chr8	81614	SNV	C	T	1	ISSFT-021, IF1SW-F4	Afu8g00370:c.5215G>A	Afu8g00370:p.Ala1739Thr	Yes	Cluster_25_fumagillin/pseurotin
Chr8	81638	SNV	A	C	1	ISSFT-021, IF1SW-F4	Afu8g00370:c.5191T>G	Afu8g00370:p.Ser1731Ala	Yes	Cluster_25_fumagillin/pseurotin
Chr8	81788	SNV	C	T	1	ISSFT-021, IF1SW-F4	Afu8g00370:c.5041G>A	Afu8g00370:p.Asp1681Asn	Yes	Cluster_25_fumagillin/pseurotin
Chr8	81849	SNV	A	T	1	ISSFT-021, IF1SW-F4	Afu8g00370:c.4980T>A		No	Cluster_25_fumagillin/pseurotin
Chr8	81959	SNV	C	T	1	CEA10	Afu8g00370:c.4870G>A	Afu8g00370:p.Val1624Ile	Yes	Cluster_25_fumagillin/pseurotin
Chr8	82017	SNV	C	A	1	CEA10	Afu8g00370:c.4812G>T		No	Cluster_25_fumagillin/pseurotin
Chr8	82041	SNV	G	T	1	CEA10	Afu8g00370:c.4788C>A		No	Cluster_25_fumagillin/pseurotin
Chr8	82140	SNV	C	T	1	CEA10	Afu8g00370:c.4689G>A		No	Cluster_25_fumagillin/pseurotin
Chr8	82342	SNV	G	T	1	ISSFT-021, IF1SW-F4	Afu8g00370:c.4487C>A	Afu8g00370:p.Pro1496His	Yes	Cluster_25_fumagillin/pseurotin
Chr8	82384	SNV	A	G	1	ISSFT-021, IF1SW-F4	Afu8g00370:c.4445T>C	Afu8g00370:p.Leu1482Pro	Yes	Cluster_25_fumagillin/pseurotin
Chr8	82493	SNV	G	A	1	ISSFT-021, IF1SW-F4	Afu8g00370:c.4336C>T	Afu8g00370:p.Arg1446Trp	Yes	Cluster_25_fumagillin/pseurotin
Chr8	83034	SNV	A	T	1	ISSFT-021, IF1SW-F4	Afu8g00370:c.3795T>A		No	Cluster_25_fumagillin/pseurotin
Chr8	83046	SNV	G	A	1	ISSFT-021, IF1SW-F4	Afu8g00370:c.3783C>T		No	Cluster_25_fumagillin/pseurotin
Chr8	83123	SNV	T	C	1	ISSFT-021, IF1SW-F4	Afu8g00370:c.3706A>G	Afu8g00370:p.Asn1236Asp	Yes	Cluster_25_fumagillin/pseurotin
Chr8	83133	SNV	A	G	1	ISSFT-021, IF1SW-F4	Afu8g00370:c.3696T>C		No	Cluster_25_fumagillin/pseurotin
Chr8	83363..83364	MNV	AA	GG	2	IF1SW-F4	Afu8g00370:c.3465_3466delTTinsCC		No	Cluster_25_fumagillin/pseurotin
Chr8	83382	SNV	C	T	1	CEA10, ISSFT-021	Afu8g00370:c.3447G>A		No	Cluster_25_fumagillin/pseurotin
Chr8	83536	SNV	C	T	1	ISSFT-021	Afu8g00370:c.3293G>A	Afu8g00370:p.Gly1098Glu	Yes	Cluster_25_fumagillin/pseurotin
Chr8	83672	SNV	C	T	1	IF1SW-F4	Afu8g00370:c.3157G>A	Afu8g00370:p.Ala1053Thr	Yes	Cluster_25_fumagillin/pseurotin
Chr8	83820	SNV	C	A	1	IF1SW-F4	Afu8g00370:c.3009G>T		No	Cluster_25_fumagillin/pseurotin
Chr8	83847	SNV	C	T	1	IF1SW-F4	Afu8g00370:c.2982G>A		No	Cluster_25_fumagillin/pseurotin

Chr8	83943	SNV	G	A	1	CEA10, ISSFT-021, IF1SW-F4	Afu8g00370:c.2886C>T		No	Cluster_25_fumagillin/pseurotin
Chr8	84114	SNV	G	A	1	IF1SW-F4	Afu8g00370:c.2715C>T		No	Cluster_25_fumagillin/pseurotin
Chr8	84747	SNV	T	A	1	ISSFT-021	Afu8g00370:c.2082A>T		No	Cluster_25_fumagillin/pseurotin
Chr8	84789	SNV	T	C	1	CEA10, ISSFT-021, IF1SW-F4	Afu8g00370:c.2040A>G		No	Cluster_25_fumagillin/pseurotin
Chr8	84908	SNV	A	G	1	ISSFT-021	Afu8g00370:c.1921T>C	Afu8g00370:p.Ser641Pro	Yes	Cluster_25_fumagillin/pseurotin
Chr8	85006	SNV	T	A	1	ISSFT-021	Afu8g00370:c.1823A>T	Afu8g00370:p.Gln608Leu	Yes	Cluster_25_fumagillin/pseurotin
Chr8	85874	SNV	G	C	1	CEA10	Afu8g00370:c.955C>G	Afu8g00370:p.Gln319Glu	Yes	Cluster_25_fumagillin/pseurotin
Chr8	85881	SNV	A	G	1	ISSFT-021	Afu8g00370:c.948T>C		No	Cluster_25_fumagillin/pseurotin
Chr8	86312	SNV	A	G	1	ISSFT-021	Afu8g00370:c.576T>C		No	Cluster_25_fumagillin/pseurotin
Chr8	86819	SNV	T	C	1	CEA10, ISSFT-021, IF1SW-F4	Afu8g00370:c.69A>G		No	Cluster_25_fumagillin/pseurotin
Chr8	86899	SNV	A	G	1	CEA10, ISSFT-021, IF1SW-F4			-	Cluster_25_fumagillin/pseurotin
Chr8	86948	SNV	T	C	1	CEA10, ISSFT-021, IF1SW-F4			-	Cluster_25_fumagillin/pseurotin
Chr8	86963	SNV	C	A	1	CEA10, ISSFT-021, IF1SW-F4			-	Cluster_25_fumagillin/pseurotin
Chr8	87498	SNV	G	A	1	IF1SW-F4	Afu8g00380:c.105+24G>A		-	Cluster_25_fumagillin/pseurotin
Chr8	87989	SNV	A	G	1	CEA10, ISSFT-021, IF1SW-F4	Afu8g00380:c.573A>G		No	Cluster_25_fumagillin/pseurotin
Chr8	88148	SNV	A	G	1	CEA10, ISSFT-021, IF1SW-F4	Afu8g00380:c.732A>G		No	Cluster_25_fumagillin/pseurotin
Chr8	88306	SNV	T	C	1	CEA10, ISSFT-021, IF1SW-F4			-	Cluster_25_fumagillin/pseurotin
Chr8	88351..88353	MNV	TAG	CAT	3	CEA10, ISSFT-021, IF1SW-F4			-	Cluster_25_fumagillin/pseurotin
Chr8	88358	SNV	G	A	1	CEA10, ISSFT-021, IF1SW-F4			-	Cluster_25_fumagillin/pseurotin
Chr8	88391	SNV	G	C	1	CEA10, ISSFT-021, IF1SW-F4			-	Cluster_25_fumagillin/pseurotin
Chr8	88436	SNV	A	C	1	CEA10, ISSFT-021, IF1SW-F4			-	Cluster_25_fumagillin/pseurotin
Chr8	88841	SNV	C	T	1	ISSFT-021	Afu8g00390:c.436+20G>A		-	Cluster_25_fumagillin/pseurotin
Chr8	89178	SNV	T	C	1	ISSFT-021	Afu8g00390:c.119A>G	Afu8g00390:p.Asn40Ser	Yes	Cluster_25_fumagillin/pseurotin
Chr8	89637	SNV	C	G	1	ISSFT-021	Afu8g00400:c.234G>C		No	Cluster_25_fumagillin/pseurotin
Chr8	89853	SNV	T	G	1	ISSFT-021	Afu8g00400:c.18A>C	Afu8g00400:p.Glu6Asp	Yes	Cluster_25_fumagillin/pseurotin
Chr8	90153	SNV	T	G	1	CEA10, ISSFT-021			-	Cluster_25_fumagillin/pseurotin
Chr8	90367	SNV	A	G	1	CEA10, ISSFT-021	Afu8g00410:c.1630-12T>C		-	Cluster_25_fumagillin/pseurotin
Chr8	90532..90533	MNV	GT	TA	2	CEA10, ISSFT-021	fu8g00410:c.1542+15_1542+16delACinsT		-	Cluster_25_fumagillin/pseurotin
Chr8	91741	SNV	T	C	1	ISSFT-021, IF1SW-F4	Afu8g00410:c.391+25A>G		-	Cluster_25_fumagillin/pseurotin
Chr8	91919	SNV	C	T	1	CEA10, ISSFT-021, IF1SW-F4	Afu8g00410:c.317G>A	Afu8g00410:p.Ser106Asn	Yes	Cluster_25_fumagillin/pseurotin
Chr8	92300	SNV	G	T	1	ISSFT-021, IF1SW-F4			-	Cluster_25_fumagillin/pseurotin
Chr8	92569	SNV	C	A	1	ISSFT-021, IF1SW-F4			-	Cluster_25_fumagillin/pseurotin
Chr8	92607	SNV	G	A	1	ISSFT-021, IF1SW-F4			-	Cluster_25_fumagillin/pseurotin
Chr8	92662	SNV	T	C	1	CEA10			-	Cluster_25_fumagillin/pseurotin
Chr8	92689	SNV	A	G	1	CEA10			-	Cluster_25_fumagillin/pseurotin
Chr8	92747	SNV	A	G	1	CEA10			-	Cluster_25_fumagillin/pseurotin
Chr8	92792	SNV	C	T	1	ISSFT-021, IF1SW-F4			-	Cluster_25_fumagillin/pseurotin
Chr8	93213^93214	Insertion	-	G	1	CEA10	Afu8g00420:c.101_102insG	Afu8g00420:p.Gly35fs	Yes	Cluster_25_fumagillin/pseurotin
Chr8	93610	SNV	C	A	1	CEA10, ISSFT-021, IF1SW-F4	Afu8g00420:c.443C>A	Afu8g00420:p.Pro148His	Yes	Cluster_25_fumagillin/pseurotin
Chr8	94117	SNV	G	A	1	IF1SW-F4	Afu8g00420:c.885G>A	Afu8g00420:p.Met295Ile	Yes	Cluster_25_fumagillin/pseurotin
Chr8	94155	SNV	G	A	1	ISSFT-021, IF1SW-F4	Afu8g00420:c.923G>A	Afu8g00420:p.Gly308Glu	Yes	Cluster_25_fumagillin/pseurotin
Chr8	94967	SNV	G	A	1	ISSFT-021	Afu8g00420:c.1735G>A	Afu8g00420:p.Gly579Arg	Yes	Cluster_25_fumagillin/pseurotin
Chr8	96706	SNV	T	C	1	ISSFT-021, IF1SW-F4	Afu8g00440:c.2264A>G	Afu8g00440:p.Asn755Ser	Yes	Cluster_25_fumagillin/pseurotin
Chr8	96750	SNV	G	A	1	ISSFT-021, IF1SW-F4	Afu8g00440:c.2220C>T		No	Cluster_25_fumagillin/pseurotin
Chr8	96855	SNV	A	G	1	ISSFT-021, IF1SW-F4	Afu8g00440:c.2115T>C		No	Cluster_25_fumagillin/pseurotin
Chr8	97065	SNV	G	T	1	IF1SW-F4	Afu8g00440:c.1905C>A		No	Cluster_25_fumagillin/pseurotin
Chr8	97145	SNV	C	G	1	IF1SW-F4	Afu8g00440:c.1825G>C	Afu8g00440:p.Ala609Pro	Yes	Cluster_25_fumagillin/pseurotin
Chr8	97329	SNV	C	T	1	IF1SW-F4	Afu8g00440:c.1641G>A		No	Cluster_25_fumagillin/pseurotin
Chr8	97416	SNV	C	T	1	IF1SW-F4	Afu8g00440:c.1554G>A		No	Cluster_25_fumagillin/pseurotin
Chr8	97431	SNV	G	A	1	IF1SW-F4	Afu8g00440:c.1539C>T		No	Cluster_25_fumagillin/pseurotin
Chr8	97470	SNV	G	A	1	IF1SW-F4	Afu8g00440:c.1500C>T		No	Cluster_25_fumagillin/pseurotin
Chr8	97524	SNV	A	C	1	ISSFT-021	Afu8g00440:c.1446T>G		No	Cluster_25_fumagillin/pseurotin
Chr8	97596	SNV	C	A	1	IF1SW-F4	Afu8g00440:c.1374G>T		No	Cluster_25_fumagillin/pseurotin
Chr8	97704	SNV	G	A	1	IF1SW-F4	Afu8g00440:c.1266C>T		No	Cluster_25_fumagillin/pseurotin
Chr8	97775	SNV	T	C	1	CEA10	Afu8g00440:c.1195A>G	Afu8g00440:p.Arg399Gly	Yes	Cluster_25_fumagillin/pseurotin
Chr8	98166	SNV	T	C	1	IF1SW-F4	Afu8g00440:c.804A>G		No	Cluster_25_fumagillin/pseurotin
Chr8	98187	SNV	C	G	1	IF1SW-F4	Afu8g00440:c.783G>C		No	Cluster_25_fumagillin/pseurotin
Chr8	98291	SNV	C	T	1	IF1SW-F4	Afu8g00440:c.679G>A	Afu8g00440:p.Glu227Lys	Yes	Cluster_25_fumagillin/pseurotin
Chr8	98394	SNV	G	A	1	CEA10	Afu8g00440:c.576C>T		No	Cluster_25_fumagillin/pseurotin

Chr8	98872	SNV	G	A	1	IF1SW-F4	Afu8g00440:c.98C>T	Afu8g00440:p.Ala33Val	Yes	Cluster_25_fumagillin/pseurotin
Chr8	99138	SNV	A	G	1	IF1SW-F4	Afu8g00440:c.-169T>C		-	Cluster_25_fumagillin/pseurotin
Chr8	99197	SNV	A	C	1	ISSFT-021	Afu8g00440:c.-228T>G		-	Cluster_25_fumagillin/pseurotin
Chr8	99205	SNV	A	G	1	IF1SW-F4	Afu8g00440:c.-236T>C		-	Cluster_25_fumagillin/pseurotin
Chr8	99353	SNV	G	A	1	IF1SW-F4	Afu8g00440:c.-384C>T		-	Cluster_25_fumagillin/pseurotin
Chr8	99383	SNV	T	C	1	IF1SW-F4	Afu8g00440:c.-414A>G		-	Cluster_25_fumagillin/pseurotin
Chr8	99388	SNV	G	A	1	CEA10, IF1SW-F4	Afu8g00440:c.-419C>T		-	Cluster_25_fumagillin/pseurotin
Chr8	99399	SNV	T	G	1	IF1SW-F4	Afu8g00440:c.-430A>C		-	Cluster_25_fumagillin/pseurotin
Chr8	99469	SNV	G	A	1	IF1SW-F4	Afu8g00440:c.-500C>T		-	Cluster_25_fumagillin/pseurotin
Chr8	99521	SNV	T	A	1	IF1SW-F4	Afu8g00440:c.-552A>T		-	Cluster_25_fumagillin/pseurotin
Chr8	99536	SNV	G	A	1	ISSFT-021	Afu8g00440:c.-567C>T		-	Cluster_25_fumagillin/pseurotin
Chr8	99568..99572	MNV	GATTG	CATTA	5	IF1SW-F4	fu8g00440:c.-603_-599delCAATCinsTAAT		-	Cluster_25_fumagillin/pseurotin
Chr8	99998	SNV	G	A	1	CEA10, ISSFT-021, IF1SW-F4	Afu8g00440:c.-1029C>T		-	Cluster_25_fumagillin/pseurotin
Chr8	100200	SNV	T	C	1	IF1SW-F4			-	Cluster_25_fumagillin/pseurotin
Chr8	100234	SNV	C	T	1	ISSFT-021, IF1SW-F4			-	Cluster_25_fumagillin/pseurotin
Chr8	100264	SNV	A	G	1	IF1SW-F4			-	Cluster_25_fumagillin/pseurotin
Chr8	100378	SNV	A	C	1	IF1SW-F4			-	Cluster_25_fumagillin/pseurotin
Chr8	100407	SNV	C	G	1	IF1SW-F4			-	Cluster_25_fumagillin/pseurotin
Chr8	100446	SNV	C	A	1	IF1SW-F4			-	Cluster_25_fumagillin/pseurotin
Chr8	100489	SNV	G	A	1	IF1SW-F4	Afu8g00460:c.-428G>A		-	Cluster_25_fumagillin/pseurotin
Chr8	100542	SNV	G	C	1	IF1SW-F4	Afu8g00460:c.-375G>C		-	Cluster_25_fumagillin/pseurotin
Chr8	100572	SNV	C	G	1	IF1SW-F4	Afu8g00460:c.-345C>G		-	Cluster_25_fumagillin/pseurotin
Chr8	100606	SNV	T	G	1	ISSFT-021	Afu8g00460:c.-311T>G		-	Cluster_25_fumagillin/pseurotin
Chr8	100675	SNV	A	C	1	CEA10	Afu8g00460:c.-242A>C		-	Cluster_25_fumagillin/pseurotin
Chr8	100713	SNV	G	C	1	ISSFT-021	Afu8g00460:c.-204G>C		-	Cluster_25_fumagillin/pseurotin
Chr8	100779^100780	Insertion	-	AACAT	5	ISSFT-021	Afu8g00460:c.-138_-137insAACAT		-	Cluster_25_fumagillin/pseurotin
Chr8	101470	SNV	C	T	1	CEA10	Afu8g00460:c.435C>T		No	Cluster_25_fumagillin/pseurotin
Chr8	103003	SNV	T	G	1	IF1SW-F4	Afu8g00470:c.299A>C	Afu8g00470:p.Lys100Thr	Yes	Cluster_25_fumagillin/pseurotin
Chr8	103020	SNV	G	T	1	IF1SW-F4	Afu8g00470:c.282C>A		No	Cluster_25_fumagillin/pseurotin
Chr8	103206	SNV	G	A	1	CEA10, ISSFT-021	Afu8g00470:c.96C>T		No	Cluster_25_fumagillin/pseurotin
Chr8	103469	SNV	G	A	1	IF1SW-F4			-	Cluster_25_fumagillin/pseurotin
Chr8	103598	SNV	G	A	1	CEA10, ISSFT-021, IF1SW-F4			-	Cluster_25_fumagillin/pseurotin
Chr8	103609	SNV	G	T	1	IF1SW-F4			-	Cluster_25_fumagillin/pseurotin
Chr8	103626	SNV	C	T	1	CEA10, ISSFT-021, IF1SW-F4			-	Cluster_25_fumagillin/pseurotin
Chr8	103631	SNV	C	T	1	IF1SW-F4			-	Cluster_25_fumagillin/pseurotin
Chr8	103689	SNV	G	C	1	IF1SW-F4			-	Cluster_25_fumagillin/pseurotin
Chr8	103771	SNV	G	A	1	IF1SW-F4	Afu8g00480:c.7G>A	Afu8g00480:p.Gly3Ser	Yes	Cluster_25_fumagillin/pseurotin
Chr8	103847	SNV	T	C	1	IF1SW-F4	Afu8g00480:c.83T>C	Afu8g00480:p.Val28Ala	Yes	Cluster_25_fumagillin/pseurotin
Chr8	103905	SNV	A	G	1	IF1SW-F4	Afu8g00480:c.141A>G		No	Cluster_25_fumagillin/pseurotin
Chr8	103995	SNV	G	A	1	IF1SW-F4	Afu8g00480:c.231G>A		No	Cluster_25_fumagillin/pseurotin
Chr8	104712	SNV	G	A	1	IF1SW-F4			-	Cluster_25_fumagillin/pseurotin
Chr8	104786	SNV	T	G	1	IF1SW-F4			-	Cluster_25_fumagillin/pseurotin
Chr8	104874	SNV	T	C	1	IF1SW-F4			-	Cluster_25_fumagillin/pseurotin
Chr8	104906	SNV	T	C	1	IF1SW-F4			-	Cluster_25_fumagillin/pseurotin
Chr8	104996	SNV	C	T	1	ISSFT-021			-	Cluster_25_fumagillin/pseurotin
Chr8	105150	SNV	A	G	1	CEA10, ISSFT-021, IF1SW-F4	Afu8g00490:c.56A>G	Afu8g00490:p.Tyr19Cys	Yes	Cluster_25_fumagillin/pseurotin
Chr8	105158	SNV	G	A	1	CEA10, ISSFT-021, IF1SW-F4	Afu8g00490:c.64G>A	Afu8g00490:p.Gly22Arg	Yes	Cluster_25_fumagillin/pseurotin
Chr8	105380	SNV	G	A	1	IF1SW-F4	Afu8g00490:c.286G>A	Afu8g00490:p.Ala96Thr	Yes	Cluster_25_fumagillin/pseurotin
Chr8	105498	SNV	T	G	1	IF1SW-F4	Afu8g00490:c.404T>G	Afu8g00490:p.Phe135Cys	Yes	Cluster_25_fumagillin/pseurotin
Chr8	106419	SNV	A	G	1	IF1SW-F4	Afu8g00490:c.1325A>G	Afu8g00490:p.Asp442Gly	Yes	Cluster_25_fumagillin/pseurotin
Chr8	106455	SNV	C	T	1	IF1SW-F4	Afu8g00490:c.1361C>T	Afu8g00490:p.Ala454Val	Yes	Cluster_25_fumagillin/pseurotin
Chr8	106576	SNV	A	C	1	IF1SW-F4	Afu8g00490:c.1482A>C		No	Cluster_25_fumagillin/pseurotin
Chr8	106711	SNV	G	A	1	IF1SW-F4	Afu8g00490:c.1617G>A		No	Cluster_25_fumagillin/pseurotin
Chr8	106975	SNV	C	A	1	CEA10, ISSFT-021	Afu8g00490:c.1881C>A		No	Cluster_25_fumagillin/pseurotin
Chr8	109144	SNV	T	C	1	CEA10, ISSFT-021, IF1SW-F4	Afu8g00500:c.1380A>G		No	Cluster_25_fumagillin/pseurotin
Chr8	110125	SNV	A	G	1	CEA10	Afu8g00500:c.399T>C		No	Cluster_25_fumagillin/pseurotin
Chr8	110178	SNV	T	C	1	CEA10, ISSFT-021, IF1SW-F4	Afu8g00500:c.346A>G	Afu8g00500:p.Thr116Ala	Yes	Cluster_25_fumagillin/pseurotin
Chr8	110559	SNV	G	T	1	ISSFT-021			-	Cluster_25_fumagillin/pseurotin
Chr8	110634	SNV	A	G	1	ISSFT-021, IF1SW-F4			-	Cluster_25_fumagillin/pseurotin

Chr8	111223	SNV	A	T	1	IF1SW-F4	Afu8g00510:c.1320T>A		No	Cluster_25_fumagillin/pseurotin
Chr8	112309	SNV	T	C	1	CEA10, ISSFT-021, IF1SW-F4	Afu8g00510:c.288A>G		No	Cluster_25_fumagillin/pseurotin
Chr8	113449	SNV	T	C	1	CEA10, ISSFT-021, IF1SW-F4			-	Cluster_25_fumagillin/pseurotin
Chr8	113902	SNV	C	T	1	IF1SW-F4	Afu8g00520:c.159C>T		No	Cluster_25_fumagillin/pseurotin
Chr8	114289	SNV	C	G	1	IF1SW-F4	Afu8g00520:c.426C>G		No	Cluster_25_fumagillin/pseurotin
Chr8	114377^114378	Insertion	-	T	1	IF1SW-F4	Afu8g00520:c.495+19_495+20insT		-	Cluster_25_fumagillin/pseurotin
Chr8	114596	SNV	T	C	1	IF1SW-F4	Afu8g00520:c.*115T>C		-	Cluster_25_fumagillin/pseurotin
Chr8	114672	SNV	T	C	1	IF1SW-F4			-	Cluster_25_fumagillin/pseurotin
Chr8	114752	SNV	G	C	1	IF1SW-F4			-	Cluster_25_fumagillin/pseurotin
Chr8	114795	SNV	C	G	1	IF1SW-F4			-	Cluster_25_fumagillin/pseurotin
Chr8	114839..114840	MNV	CT	TC	2	IF1SW-F4			-	Cluster_25_fumagillin/pseurotin
Chr8	114938^114939	Insertion	-	TGCATAACA	15	IF1SW-F4	8g00530:c.*279_*280insTGTTGTTATGCA		-	Cluster_25_fumagillin/pseurotin
Chr8	115051	SNV	C	A	1	IF1SW-F4	Afu8g00530:c.*167G>T		-	Cluster_25_fumagillin/pseurotin
Chr8	115165	SNV	T	C	1	ISSFT-021	Afu8g00530:c.*53A>G		-	Cluster_25_fumagillin/pseurotin
Chr8	116714	SNV	C	A	1	CEA10	Afu8g00530:c.-159G>T		-	Cluster_25_fumagillin/pseurotin
Chr8	116829	SNV	T	A	1	CEA10, ISSFT-021, IF1SW-F4			-	Cluster_25_fumagillin/pseurotin
Chr8	116862	SNV	T	A	1	CEA10, ISSFT-021, IF1SW-F4			-	Cluster_25_fumagillin/pseurotin
Chr8	116885..116886	Deletion	AG	-	2	CEA10, ISSFT-021, IF1SW-F4			-	Cluster_25_fumagillin/pseurotin
Chr8	117016	SNV	T	C	1	ISSFT-021, IF1SW-F4	Afu8g00540:c.-2T>C		-	Cluster_25_fumagillin/pseurotin
Chr8	118521	SNV	C	G	1	CEA10, ISSFT-021, IF1SW-F4	Afu8g00540:c.1385C>G	Afu8g00540:p.Thr462Arg	Yes	Cluster_25_fumagillin/pseurotin
Chr8	118630	SNV	A	G	1	CEA10, ISSFT-021, IF1SW-F4	Afu8g00540:c.1494A>G		No	Cluster_25_fumagillin/pseurotin
Chr8	118831	SNV	T	C	1	CEA10, ISSFT-021, IF1SW-F4	Afu8g00540:c.1695T>C		No	Cluster_25_fumagillin/pseurotin
Chr8	118901	SNV	G	C	1	CEA10, ISSFT-021, IF1SW-F4	Afu8g00540:c.1765G>C	Afu8g00540:p.Gly589Arg	Yes	Cluster_25_fumagillin/pseurotin
Chr8	118906	SNV	G	T	1	CEA10, ISSFT-021, IF1SW-F4	Afu8g00540:c.1770G>T		No	Cluster_25_fumagillin/pseurotin
Chr8	120350	SNV	G	C	1	CEA10, ISSFT-021, IF1SW-F4	Afu8g00540:c.3214G>C	Afu8g00540:p.Asp1072His	Yes	Cluster_25_fumagillin/pseurotin
Chr8	121708	SNV	A	G	1	CEA10	Afu8g00540:c.4572A>G		No	Cluster_25_fumagillin/pseurotin
Chr8	121940	SNV	G	A	1	CEA10	Afu8g00540:c.4804G>A	Afu8g00540:p.Val1602Met	Yes	Cluster_25_fumagillin/pseurotin
Chr8	122876	SNV	G	A	1	ISSFT-021, IF1SW-F4	Afu8g00540:c.5740G>A	Afu8g00540:p.Val1914Met	Yes	Cluster_25_fumagillin/pseurotin
Chr8	123173	SNV	G	T	1	ISSFT-021, IF1SW-F4	Afu8g00540:c.6037G>T	Afu8g00540:p.Val2013Leu	Yes	Cluster_25_fumagillin/pseurotin
Chr8	123345	SNV	G	A	1	CEA10, ISSFT-021, IF1SW-F4	Afu8g00540:c.6209G>A	Afu8g00540:p.Arg2070Gln	Yes	Cluster_25_fumagillin/pseurotin
Chr8	123797	SNV	A	G	1	ISSFT-021	Afu8g00540:c.6604A>G	Afu8g00540:p.Ile2202Val	Yes	Cluster_25_fumagillin/pseurotin
Chr8	125658	SNV	T	C	1	CEA10, ISSFT-021, IF1SW-F4	Afu8g00540:c.8359T>C	Afu8g00540:p.Ser2787Pro	Yes	Cluster_25_fumagillin/pseurotin
Chr8	126143	SNV	G	A	1	ISSFT-021, IF1SW-F4	Afu8g00540:c.8844G>A		No	Cluster_25_fumagillin/pseurotin
Chr8	126512	SNV	C	T	1	CEA10, ISSFT-021, IF1SW-F4	Afu8g00540:c.9213C>T		No	Cluster_25_fumagillin/pseurotin
Chr8	126517	SNV	A	G	1	CEA10, ISSFT-021, IF1SW-F4	Afu8g00540:c.9218A>G	Afu8g00540:p.Lys3073Arg	Yes	Cluster_25_fumagillin/pseurotin
Chr8	126620	SNV	T	G	1	CEA10, ISSFT-021, IF1SW-F4	Afu8g00540:c.9321T>G	Afu8g00540:p.Asp3107Glu	Yes	Cluster_25_fumagillin/pseurotin
Chr8	126800	SNV	C	T	1	IF1SW-F4	Afu8g00540:c.9501C>T		No	Cluster_25_fumagillin/pseurotin
Chr8	127442	SNV	G	A	1	CEA10, ISSFT-021, IF1SW-F4	Afu8g00540:c.10143G>A		No	Cluster_25_fumagillin/pseurotin
Chr8	127505	SNV	T	C	1	CEA10, ISSFT-021, IF1SW-F4	Afu8g00540:c.10206T>C		No	Cluster_25_fumagillin/pseurotin
Chr8	127517	SNV	C	G	1	CEA10, ISSFT-021, IF1SW-F4	Afu8g00540:c.10218C>G		No	Cluster_25_fumagillin/pseurotin
Chr8	127534	SNV	A	G	1	IF1SW-F4	Afu8g00540:c.10235A>G	Afu8g00540:p.Glu3412Gly	Yes	Cluster_25_fumagillin/pseurotin
Chr8	127560	SNV	G	A	1	CEA10, ISSFT-021	Afu8g00540:c.10261G>A	Afu8g00540:p.Ala3421Thr	Yes	Cluster_25_fumagillin/pseurotin
Chr8	127581..127583	MNV	TTT	CTG	3	CEA10, ISSFT-021, IF1SW-F4	Afu8g00540:c.10282_10284delTTTinsCTG	Afu8g00540:p.Phe3428Leu	Yes	Cluster_25_fumagillin/pseurotin
Chr8	127985	SNV	A	T	1	CEA10, ISSFT-021	Afu8g00540:c.10686A>T		No	Cluster_25_fumagillin/pseurotin
Chr8	128017	SNV	A	G	1	IF1SW-F4	Afu8g00540:c.10718A>G	Afu8g00540:p.Glu3573Gly	Yes	Cluster_25_fumagillin/pseurotin
Chr8	128069	SNV	A	G	1	CEA10, ISSFT-021	Afu8g00540:c.10770A>G		No	Cluster_25_fumagillin/pseurotin
Chr8	128273	SNV	C	A	1	IF1SW-F4	Afu8g00540:c.10974C>A	Afu8g00540:p.Asn3658Lys	Yes	Cluster_25_fumagillin/pseurotin
Chr8	128285	SNV	A	G	1	CEA10, ISSFT-021	Afu8g00540:c.10986A>G		No	Cluster_25_fumagillin/pseurotin
Chr8	128313	SNV	C	A	1	CEA10, ISSFT-021	Afu8g00540:c.11014C>A	Afu8g00540:p.Leu3672Ile	Yes	Cluster_25_fumagillin/pseurotin
Chr8	128418	SNV	A	G	1	CEA10, ISSFT-021	Afu8g00540:c.11119A>G	Afu8g00540:p.Ile3707Val	Yes	Cluster_25_fumagillin/pseurotin
Chr8	128448	SNV	G	A	1	CEA10, ISSFT-021	Afu8g00540:c.11149G>A	Afu8g00540:p.Val3717Ile	Yes	Cluster_25_fumagillin/pseurotin
Chr8	128482	SNV	A	G	1	CEA10, ISSFT-021	Afu8g00540:c.11183A>G	Afu8g00540:p.Asn3728Ser	Yes	Cluster_25_fumagillin/pseurotin
Chr8	129269	SNV	G	C	1	CEA10, ISSFT-021, IF1SW-F4	Afu8g00540:c.11970G>C		No	Cluster_25_fumagillin/pseurotin
Chr8	129282	SNV	A	G	1	IF1SW-F4	Afu8g00540:c.11983A>G	Afu8g00540:p.Ile3995Val	Yes	Cluster_25_fumagillin/pseurotin
Chr8	129357	SNV	A	G	1	IF1SW-F4			-	Cluster_25_fumagillin/pseurotin
Chr8	130803	SNV	G	A	1	CEA10, ISSFT-021, IF1SW-F4			-	Cluster_25_fumagillin/pseurotin
Chr8	130832	SNV	G	A	1	IF1SW-F4			-	Cluster_25_fumagillin/pseurotin
Chr8	130850	SNV	G	A	1	CEA10, ISSFT-021, IF1SW-F4			-	Cluster_25_fumagillin/pseurotin
Chr8	131633	SNV	G	C	1	CEA10, ISSFT-021, IF1SW-F4	Afu8g00560:c.537G>C		No	Cluster_25_fumagillin/pseurotin

Chr8	131932	SNV	A	G	1	CEA10, ISSFT-021, IF1SW-F4	Afu8g00560:c.836A>G	Afu8g00560:p.Asn279Ser	Yes	Cluster_25_fumagillin/pseurotin
Chr8	132038	SNV	T	C	1	CEA10, ISSFT-021, IF1SW-F4	Afu8g00560:c.942T>C		No	Cluster_25_fumagillin/pseurotin
Chr8	132077	SNV	A	G	1	CEA10, ISSFT-021, IF1SW-F4	Afu8g00560:c.981A>G		No	Cluster_25_fumagillin/pseurotin
Chr8	132089	SNV	G	A	1	CEA10, ISSFT-021, IF1SW-F4	Afu8g00560:c.993G>A		No	Cluster_25_fumagillin/pseurotin
Chr8	132357	SNV	C	T	1	CEA10, ISSFT-021, IF1SW-F4	Afu8g00560:c.1200C>T		No	Cluster_25_fumagillin/pseurotin
Chr8	132777	SNV	A	G	1	CEA10, ISSFT-021, IF1SW-F4			-	Cluster_25_fumagillin/pseurotin
Chr8	133855	SNV	T	A	1	CEA10, ISSFT-021, IF1SW-F4	Afu8g00570:c.162A>T		No	Cluster_25_fumagillin/pseurotin
Chr8	134161	SNV	G	A	1	ISSFT-021, IF1SW-F4			-	Cluster_25_fumagillin/pseurotin
Chr8	135434	SNV	T	C	1	CEA10, ISSFT-021, IF1SW-F4	Afu8g00580:c.172A>G	Afu8g00580:p.Ser58Gly	Yes	Cluster_25_fumagillin/pseurotin
Chr8	631505	SNV	T	C	1	ISSFT-021, IF1SW-F4	Afu8g02350:c.46T>C	Afu8g02350:p.Ser16Pro	Yes	Cluster_26_TC/PKS
Chr8	631822	SNV	T	G	1	ISSFT-021, IF1SW-F4	Afu8g02350:c.363T>G	Afu8g02350:p.His121Gln	Yes	Cluster_26_TC/PKS
Chr8	632120	SNV	A	G	1	CEA10	Afu8g02350:c.630+31A>G		-	Cluster_26_TC/PKS
Chr8	632126	SNV	A	G	1	ISSFT-021, IF1SW-F4	Afu8g02350:c.631-26A>G		-	Cluster_26_TC/PKS
Chr8	632610	SNV	C	A	1	CEA10	Afu8g02350:c.1089C>A	Afu8g02350:p.Phe363Leu	Yes	Cluster_26_TC/PKS
Chr8	633125	SNV	G	T	1	ISSFT-021	Afu8g02350:c.1604G>T	Afu8g02350:p.Ser535Ile	Yes	Cluster_26_TC/PKS
Chr8	634622	SNV	T	C	1	ISSFT-021, IF1SW-F4	Afu8g02350:c.3101T>C	Afu8g02350:p.Val1034Ala	Yes	Cluster_26_TC/PKS
Chr8	635137	SNV	A	G	1	IF1SW-F4	Afu8g02350:c.3616A>G	Afu8g02350:p.Thr1206Ala	Yes	Cluster_26_TC/PKS
Chr8	637468	SNV	G	A	1	ISSFT-021	Afu8g02360:c.1691C>T	Afu8g02360:p.Pro564Leu	Yes	Cluster_26_TC/PKS
Chr8	637525	SNV	G	A	1	ISSFT-021	Afu8g02360:c.1634C>T	Afu8g02360:p.Ala545Val	Yes	Cluster_26_TC/PKS
Chr8	638784	SNV	T	A	1	ISSFT-021	Afu8g02360:c.430A>T	Afu8g02360:p.Arg144*	Yes	Cluster_26_TC/PKS
Chr8	640833	SNV	T	G	1	ISSFT-021	Afu8g02380:c.296+26A>C		-	Cluster_26_TC/PKS
Chr8	641382	SNV	C	T	1	ISSFT-021			-	Cluster_26_TC/PKS
Chr8	641537	SNV	T	C	1	IF1SW-F4			-	Cluster_26_TC/PKS
Chr8	642650	SNV	C	G	1	ISSFT-021	Afu8g02390:c.189G>C		No	Cluster_26_TC/PKS
Chr8	644752	SNV	A	C	1	ISSFT-021	Afu8g02400:c.856A>C	Afu8g02400:p.Asn286His	Yes	Cluster_26_TC/PKS
Chr8	645336	SNV	A	G	1	ISSFT-021			-	Cluster_26_TC/PKS
Chr8	645481	SNV	C	G	1	ISSFT-021			-	Cluster_26_TC/PKS
Chr8	646890	SNV	A	G	1	ISSFT-021	Afu8g02410:c.606A>G		No	Cluster_26_TC/PKS
Chr8	648111	SNV	G	A	1	CEA10, IF1SW-F4			-	Cluster_26_TC/PKS
Chr8	648116	SNV	A	G	1	ISSFT-021			-	Cluster_26_TC/PKS
Chr8	648407	SNV	G	A	1	ISSFT-021	Afu8g02420:c.181G>A	Afu8g02420:p.Ala61Thr	Yes	Cluster_26_TC/PKS
Chr8	648683..648691	Deletion	AGGGGCCGG	-	9	CEA10, IF1SW-F4	Afu8g02420:c.404_412delAGGGGCCGG	Afu8g02420:p.Glu135_Pro137del	Yes	Cluster_26_TC/PKS
Chr8	648829	SNV	T	G	1	ISSFT-021	Afu8g02420:c.550T>G	Afu8g02420:p.Ser184Ala	Yes	Cluster_26_TC/PKS
Chr8	649063^649064	Insertion	-	GAAT	4	ISSFT-021	Afu8g02420:c.784_785insGAAT	Afu8g02420:p.Tyr262fs	Yes	Cluster_26_TC/PKS
Chr8	650325	SNV	A	G	1	CEA10, ISSFT-021, IF1SW-F4	Afu8g02430:c.-21T>C		-	Cluster_26_TC/PKS
Chr8	651022	SNV	A	G	1	CEA10, ISSFT-021, IF1SW-F4			-	Cluster_26_TC/PKS
Chr8	651488	SNV	T	C	1	CEA10, ISSFT-021, IF1SW-F4			-	Cluster_26_TC/PKS
Chr8	651769	SNV	T	C	1	CEA10, ISSFT-021, IF1SW-F4			-	Cluster_26_TC/PKS
Chr8	653669	SNV	T	C	1	IF1SW-F4			-	Cluster_26_TC/PKS
Chr8	654179	SNV	A	C	1	CEA10, ISSFT-021	Afu8g02440:c.835+9T>G		-	Cluster_26_TC/PKS
Chr8	656891	SNV	C	T	1	IF1SW-F4			-	Cluster_26_TC/PKS
Chr8	657180	SNV	T	C	1	CEA10, ISSFT-021			-	Cluster_26_TC/PKS
Chr8	657894	SNV	T	C	1	CEA10, ISSFT-021, IF1SW-F4	Afu8g02460:c.440+24T>C		-	Cluster_26_TC/PKS
Chr8	658778	SNV	A	G	1	CEA10, ISSFT-021	Afu8g02460:c.1072A>G	Afu8g02460:p.Thr358Ala	Yes	Cluster_26_TC/PKS
Chr8	660273	SNV	C	T	1	ISSFT-021	Afu8g02470:c.376-13C>T		-	Cluster_26_TC/PKS
Chr8	660562	SNV	G	C	1	ISSFT-021	Afu8g02470:c.652G>C	Afu8g02470:p.Gly218Arg	Yes	Cluster_26_TC/PKS
