# Supplementary material for: Characterization of Aspergillus fumigatus Isolates from Air and Surfaces of the International Space Station
Source: mSphere. 2016 Oct 26;1(5):e00227-16. doi: 10.1128/mSphere.00227-16 (PMC5082629; doi:10.1128/mSphere.00227-16)
Supplement: Figure S1 [file sph005162172sf1.pdf]

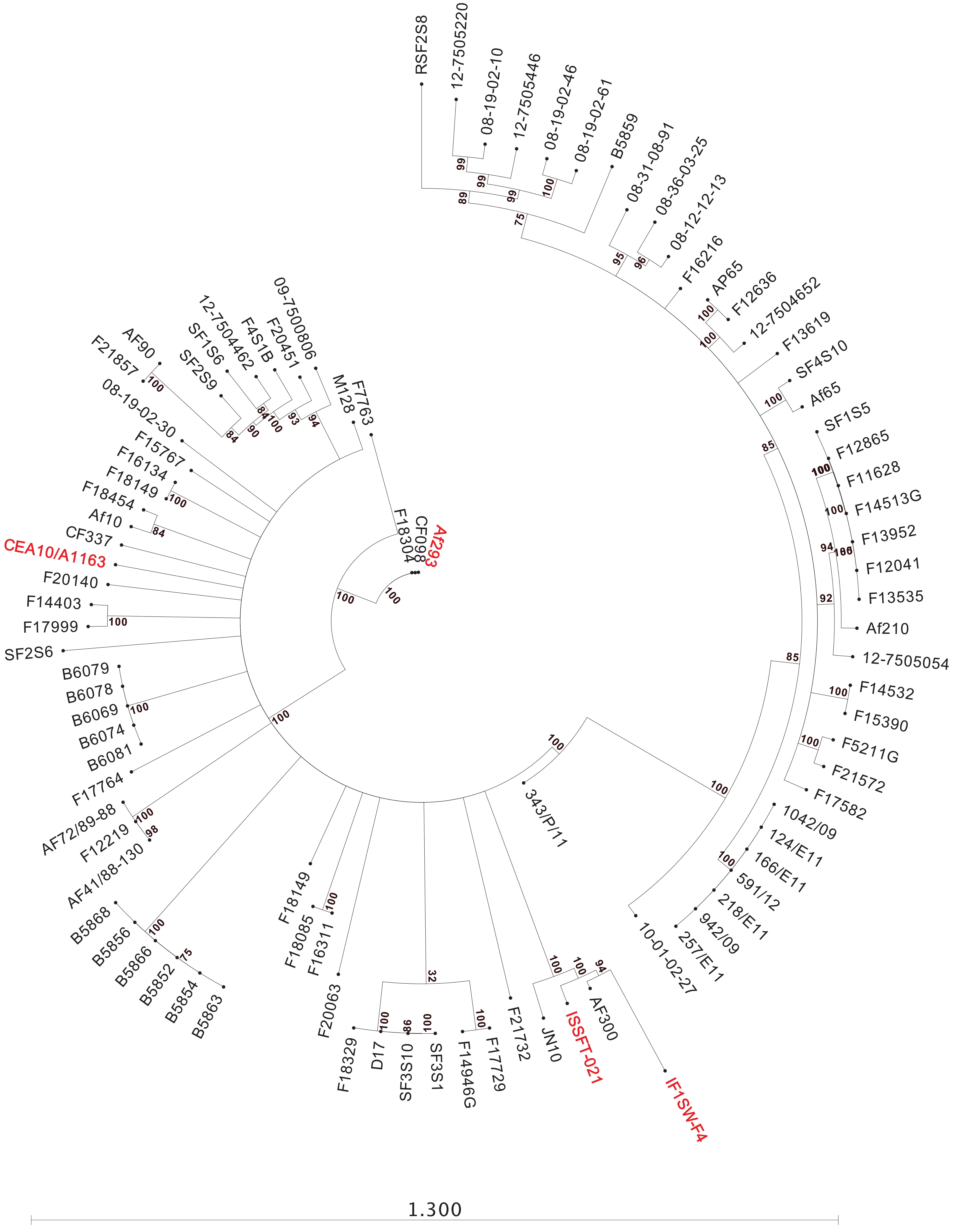
RSF2S8
12-7505220
08-19-02-10
12-7505446
08-19-02-46
08-19-02-61
B5859
08-31-08-91
08-36-03-25
08-12-12-13
F16216
AP65
F12636
12-7504652
F13619
SF4S10
Af65
SF1S5
F12865
F11628
F14513G
F13952
F12041
F13535
Af210
12-7505054
F14532
F15390
F5211G
F21572
F17582
1042/09
124/E11
166/E11
591/12
218/E11
942/09
257/E11
10-01-02-27
343/P/11
IF1SW-F4
AF300
ISSFT-021
JN10
F21732
F17729
F14946G
SF3S1
SF3S10
D17
F18329
F20063
F16311
F18085
F18149
B5863
B5854
B5852
B5866
B5856
B5868
AF41/88-130
F12219
AF72/89-88
F17764
B6081
B6074
B6069
B6078
B6079
SF2S6
F17999
F14403
F20140
CEA10/A1163
CF337
Af10
F18454
F18149
F16134
F15767
08-19-02-30
F21857
AF90
SF2S9
SF1S6
12-7504462
F4S1B
F20451
09-7500806
M128
F7763
F18304
CF098
Af293
1.300
